# Phase I metabolites of mephedrone display biological activity as substrates at monoamine transporters

**DOI:** 10.1111/bph.13547

**Published:** 2016-07-31

**Authors:** F P Mayer, L Wimmer, O Dillon‐Carter, J S Partilla, N V Burchardt, M D Mihovilovic, M H Baumann, H H Sitte

**Affiliations:** ^1^ Medical University of ViennaCenter for Physiology and Pharmacology, Institute of PharmacologyViennaAustria; ^2^ Institute of Applied Synthetic ChemistryVienna University of TechnologyViennaAustria; ^3^ Designer Drug Research Unit (DDRU)Intramural Research Program (IRP), NIDA, NIHBaltimoreMDUSA; ^4^ Center for Addiction Research and ScienceMedical University ViennaViennaAustria

## Abstract

**Background and Purpose:**

4‐Methyl‐*N‐*methylcathinone (mephedrone) is a synthetic stimulant that acts as a substrate‐type releaser at transporters for dopamine (DAT), noradrenaline (NET) and 5‐HT (SERT). Upon systemic administration, mephedrone is metabolized to several phase I compounds: the *N‐*demethylated metabolite, 4‐methylcathinone (nor‐mephedrone); the ring‐hydroxylated metabolite, 4‐hydroxytolylmephedrone (4‐OH‐mephedrone); and the reduced keto‐metabolite, dihydromephedrone.

**Experimental Approach:**

We used *in vitro* assays to compare the effects of mephedrone and synthetically prepared metabolites on transporter‐mediated uptake and release in HEK293 cells expressing human monoamine transporters and in rat brain synaptosomes. *In vivo* microdialysis was employed to examine the effects of i.v. metabolite injection (1 and 3 mg·kg^−1^) on extracellular dopamine and 5‐HT levels in rat nucleus accumbens.

**Key Results:**

In cells expressing transporters, mephedrone and its metabolites inhibited uptake, although dihydromephedrone was weak overall. In cells and synaptosomes, nor‐mephedrone and 4‐OH‐mephedrone served as transportable substrates, inducing release via monoamine transporters. When administered to rats, mephedrone and nor‐mephedrone produced elevations in extracellular dopamine and 5‐HT, whereas 4‐OH‐mephedrone did not. Mephedrone and nor‐mephedrone, but not 4‐OH‐mephedrone, induced locomotor activity.

**Conclusions and Implications:**

Our results demonstrate that phase I metabolites of mephedrone are transporter substrates (i.e. releasers) at DAT, NET and SERT, but dihydromephedrone is weak in this regard. When administered *in vivo*, nor‐mephedrone increases extracellular dopamine and 5‐HT in the brain whereas 4‐OH‐mephedrone does not, suggesting the latter metabolite does not penetrate the blood–brain barrier. Future studies should examine the pharmacokinetics of nor‐mephedrone to determine its possible contribution to the *in vivo* effects produced by mephedrone.

**Abbreviations**
4‐OH‐mephedrone: 4‐hydroxytolylmephedrone
DAT: dopamine transporter
GBR12935: 1‐(2‐diphenylmethoxyethyl)‐4‐(3‐phenylpropyl)piperazine dihydrochloride
MPP^+^: 1‐methyl‐4‐phenylpyridinium
NET: noradrenaline transporter
nor‐mephedrone: 4‐methylcathinone
PDL: poly‐d‐lysine
SERT: 5‐HT transporter

## Tables of Links

**Table:** 

**TARGETS**	
**Transporters** ^a^	**Enzymes** ^b^
DAT, SLC6A3	CYP2D6
NET, SLC6A2	
SERT, SLC6A4	
VMAT2, SLC18A2	

**LIGANDS**	
Amphetamine	MDMA
Citalopram	MPP^+^
Cocaine	Nomifensine
Desipramine	Noradrenaline
Dopamine	5‐HT
GBR12935	

These Tables list key protein targets and ligands in this article that are hyperlinked to corresponding entries in http://www.guidetopharmacology.org, the common portal for data from the IUPHAR/BPS Guide to PHARMACOLOGY (Southan *et al*., 2016), and are permanently archived in the Concise Guide to PHARMACOLOGY 2015/16 (^*a,b*^Alexander *et al*., 2015b, 2015a).

## Introduction

During the past decade, a variety of man‐made ‘designer drugs’ or ‘new psychoactive substances’ (NPS) have appeared in the recreational drug market as legal alternatives to more traditional drugs of abuse (Baumann *et al*., 2014; Sitte and Freissmuth, 2015). Frequently, the chemical structures of NPS are based on known illicit substances and mimic their psychoactive effects, but subtle structural modifications to the drug molecules render them legal (Baumann and Volkow, 2016). In particular, a number of NPS have been marketed as replacements for illicit stimulants like cocaine and 3,4‐methylenedioxymethamphetamine (MDMA, ‘ecstasy’) (Green *et al*., 2014). One of the most popular synthetic stimulants is the cathinone analogue, 4‐methyl‐*N‐*methylcathinone or mephedrone. Mephedrone first appeared in Israel as a ‘party drug’ during the early 2000s, and its recreational use spread to Europe, Australia and other parts of the world (Kelly, 2011). In the United States, mephedrone was a constituent of so‐called bath salts products, which became popular during 2010–2011 (Spiller *et al*., 2011). Low doses of mephedrone produce typical stimulant effects in humans, like increased energy and mood elevation (Vardakou *et al*., 2011; Winstock *et al*., 2011), while high doses or chronic use can produce life‐threatening side effects including tachycardia, hypertension, agitation and seizures (James *et al*., 2011; Wood *et al*., 2011). Deaths from mephedrone are rare but have been reported (Loi *et al*., 2015). In the interest of public health and safety, legislation was passed in many countries to ban the sale, possession and use of mephedrone (Drug Enforcement Administration, 2011; Green *et al*., 2014). Despite such bans, mephedrone continues to be abused in European countries (Archer *et al*., 2014; Hondebrink *et al*., 2015; EMCDDA, 2014).

Similar to other stimulant drugs, mephedrone exerts its effects by interacting with plasma membrane monoamine transporter proteins of the solute carrier 6 family (SLC6) (Hadlock *et al*., 2011; Baumann *et al*., 2012; Martinez‐Clemente *et al*., 2012), namely the dopamine transporter (DAT, SLC6A3), noradrenaline transporter (NET, SLC6A2) and 5‐HT transporter (SERT, SLC6A4). The normal role of monoamine transporters is to capture previously released neurotransmitter molecules from the extracellular space and move them back into the neuronal cytoplasm (i.e. uptake), thus terminating monoamine signalling (Kristensen *et al*., 2011; Reith *et al*., 2015). Drugs that interact with DAT, NET and SERT can be classified as either cocaine‐like ‘blockers’ or amphetamine‐like ‘substrates’ (Rothman and Baumann, 2003; Sitte and Freissmuth, 2015). Both types of compounds disrupt transporter function and produce elevations in extracellular monoamine concentrations, but their precise modes of action are different. On a molecular level, cocaine‐like blockers act as non‐transported inhibitors of monoamine transporters. Consequently, blockers prevent the transporter‐mediated uptake of released neurotransmitters from the extracellular medium. In addition, cocaine is known to mobilize the intracellular reserve pool of dopamine and stimulate its exocytotic release (Venton *et al*., 2006). In contrast, amphetamine‐like compounds are transported substrates that not only act as competitive uptake inhibitors but also trigger neurotransmitter efflux by a complex process involving reversal of transporter flux (Chen and Reith, 2004; Reith *et al*., 2015; Sitte and Freissmuth, 2015). Consequently, drugs that act as transporter substrates are often referred to as ‘releasers’ as they induce a transporter‐mediated efflux of neurotransmitters.

Studies using *in vitro* transporter assays in cells and rat brain synaptosomes have shown that mephedrone acts as a non‐selective substrate at DAT, NET and SERT, thereby leading to efflux of dopamine, noradrenaline and 5‐HT (Baumann *et al*., 2012; Eshleman *et al*., 2013; Simmler *et al*., 2013). Systemic administration of mephedrone to rats increases the extracellular concentrations of dopamine and 5‐HT in the brain, with the effects on 5‐HT being somewhat greater in magnitude (Kehr *et al*., 2011; Baumann *et al*., 2012; Wright *et al*., 2012). Overall, the available preclinical data indicate that mephedrone displays neurochemical effects that mimic MDMA, but mephedrone has a number of physiological and toxicological properties that render it unique (Baumann *et al*., 2012; Miller *et al*., 2013; Shortall *et al*., 2013). For example, high‐dose administration of mephedrone is less apt to produce robust hyperthermia and long‐term depletions of brain tissue 5‐HT (Baumann *et al*., 2012; den Hollander *et al*., 2013; Motbey *et al*., 2012), effects that are well established for MDMA. Importantly, mephedrone has greatly reduced potency at the vesicular monoamine transporter 2 (VMAT2, SLC18A2) when compared with MDMA and other ring‐substituted amphetamines (Eshleman *et al*., 2013; Pifl *et al*., 2015), suggesting mephedrone is less likely to disrupt intracellular stores of monoamine transmitters.

**Figure 1:**
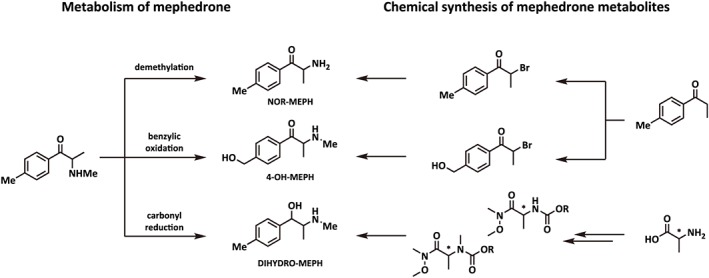
Proposed pathways for the metabolism of mephedrone to its phase I metabolites. (i) demethylation forms 4‐methylcathinone (NOR‐MEPH); (ii) benzylic oxidation forms 4‐hydroxytolylmephedrone (4‐OH‐MEPH); (iii) carbonyl reduction forms dihydromephedrone (DIHYDRO‐MEPH). Chemical synthesis started from non‐chiral precursors for the generation of racemic NOR‐MEPH and 4‐OH‐MEPH and from chiral precursors for DIHYDRO‐MEPH (racemic diastereomers obtained by mixing of enantiomers).

One possible explanation for the distinct effects of mephedrone is that metabolites of the drug contribute to its *in vivo* profile of actions. Meyer *et al*. (2010) first reported that mephedrone is metabolized by three main hepatic mechanisms (Figure 1): (i) *N‐*demethylation to form 4‐methylcathinone or nor‐mephedrone; (ii) hydroxylation of the 4‐methyl ring‐substitution to form 4‐hydroxytolylmephedrone (4‐OH‐mephedrone); and (iii) reduction of the β‐keto‐oxygen group, which forms dihydromephedrone (Meyer *et al*., 2010). Pedersen and co‐workers (2013) identified cytochrome P450 2D6 (CYP2D6) as the main enzyme responsible for the phase 1 metabolism of mephedrone in humans and detected nor‐mephedrone, 4‐OH‐mephedrone and dihydromephedrone in human urine specimens (Pedersen *et al*., 2013). As pointed out by Green *et al*. (2014), no studies have examined the pharmacology of mephedrone metabolites. Therefore, in the present investigation, we used *in vitro* assays to compare the effects of mephedrone and its metabolites on transporter‐mediated uptake and release in cells expressing human DAT, NET and SERT and in rat brain synaptosomes. Additionally, the *in vivo* neurochemical effects of systemically administered mephedrone, nor‐mephedrone or 4‐OH‐mephedrone were examined using microdialysis in rat nucleus accumbens. Our data show that phase I metabolites of mephedrone are substrates at monoamine transporters when assessed *in vitro*, but only nor‐mephedrone displays substantial neurochemical actions *in vivo*, which could contribute to the behavioural effects of systemically administered mephedrone.

## Methods

### Animals and housing

Male Sprague Dawley rats from Harlan Laboratories (Frederick, MD, USA) weighing 250–300 g at arrival were housed three per cage for 2 weeks prior to being used in experiments. The rats were housed under standard conditions (lights on from 0700–1900 h) with food and water available *ad libitum*. Rats were maintained in facilities fully accredited by the Association for Assessment and Accreditation of Laboratory Animal Care (AAALAC), and experiments were performed in accordance with the Institutional Care and Use Committee of the NIDA Intramural Research Program. Rats used for brain tissue harvest to prepare synaptosomes were housed in pairs, whereas those used in microdialysis experiments were housed singly post‐operatively (see below).

Animal studies are reported in compliance with the ARRIVE guidelines (Kilkenny *et al*., 2010; McGrath & Lilley, 2015). A total of 16 rats was used for the *in vitro* synaptosome assays, and an additional 28 rats were used for *in vivo* microdialysis experiments.

### Cell culture

The generation of HEK293 cells stably expressing the human isoforms of DAT (hDAT) and NET (hNET) was carried out as described previously (Scholze *et al*., 2002). For SERT, the human isoform (hSERT) was cloned in frame with yellow fluorescent protein (Schmid *et al*., 2001). The generation of a stable cell line was performed as described by Hilber and colleagues (Hilber *et al*., 2005). HEK293 cells were maintained in humidified atmosphere (5% CO_2_, 37°C) in DMEM, supplemented with 10% heat‐inactivated FBS and penicillin (100 u 100 mL^−1^) and streptomycin (100 μg 100 mL^−1^). Selection pressure was maintained by adding geneticin (50 μg·mL^−1^) to the cell culture media.

### Transporter uptake assays in HEK293 cells

Uptake experiments were conducted as described previously (Sitte *et al*., 2001) with minor modifications. In brief, HEK293 cells expressing hDAT, hNET or hSERT were seeded into poly‐d‐lysine (PDL) coated 96‐well plates at a density of 40 000 cells per well. The next day, DMEM was aspirated and replaced with Krebs HEPES buffer (KHB, 25 mM HEPES, 120 mM NaCl, 5 mM KCl, 1.2 mM CaCl_2_, and 1.2 mM MgSO_4_, 5 mM D‐glucose, pH adjusted to 7.3 with NaOH) (200 μL per well), and cells were pre‐incubated with various concentrations of mephedrone or its metabolites for 5 min (50 μL per well). Subsequently, 0.1 μM of [^3^H]‐5‐HT or 0.02 μM of [^3^H]‐MPP^+^ were added, and uptake was terminated after 1 (hSERT) or 3 min (hDAT, hNET) by washing the cells with 200 μL of ice‐cold KHB. Cells were lysed with 1% SDS, and tritium uptake was determined by scintillation counting. Nonspecific uptake was determined in the presence of 10 μM paroxetine (hSERT) or 10 μM mazindol (hDAT and hNET).

### Transporter release assays in HEK293 cells

Superfusion experiments were performed as described previously (Scholze *et al*., 2002). Briefly, HEK293 cells expressing the desired transporter were seeded at a density of 40 000 cells per well onto poly‐D‐lysine‐coated 5 mm glass cover slips in 96‐well plates 24 h prior to the experiment. Cells were preloaded with [^3^H]‐MPP^+^ (0.1 μM, hDAT and hNET) or [^3^H]‐5‐HT (0.4 μM, hSERT) for 20 min at 37°C in a final volume of 100 μL per well. Subsequently, glass coverslips were transferred into small superfusion chambers (volume of 200 μL) and superfused with KHB at 25°C with a superfusion rate of 0.7 mL·min^−1^ for 40 min to establish a stable basal efflux. After washout, the collection of 2‐min fractions was initiated. After the first three basal fractions, monensin (10 μM) or solvent was added for four fractions. Consequently, the cells were challenged with test drugs (10 μM) for five fractions in the presence or absence of monensin. Finally, the cells were lysed in 1% SDS to determine the total radioactivity. Radioactivity per fraction was assessed by a liquid scintillation counter and expressed as fractional release, that is, the percentage of released ^3^H in relation to total ^3^H present at the beginning of the fraction (Sitte *et al*., 2000). For analysis, release was expressed as AUC. AUC was calculated for t = 6 to 26 min and normalized to basal efflux, that is, t = 0 to 4 min.

### Transporter release assays in rat brain synaptosomes

The ability of mephedrone and its metabolites to evoke release via DAT, NET and SERT was determined in rat brain synaptosomes as previously described (Baumann *et al*., 2012). Rats were killed with CO_2_, decapitated, and brains were rapidly removed and dissected on ice. Synaptosomes were prepared from striatum for DAT assays, whereas synaptosomes were prepared from whole brain minus striatum and cerebellum for the NET and SERT assays. [^3^H]‐MPP^+^ (9 nM) was used as the radiolabelled substrate for DAT and NET, whereas [^3^H]‐5‐HT (5 nM) was used as the radiolabelled substrate for SERT. All buffers used in the release assays contained 1 μM reserpine to block vesicular uptake of substrates. The selectivity of assays was optimized for a single transporter by including unlabelled compounds [nomifensine and 1‐(2‐diphenylmethoxyethyl)‐4‐(3‐phenylpropyl)piperazine dihydrochloride (GBR12935) for SERT; GBR12935 and citalopram for NET; citalopram and desipramine for DAT] to prevent the uptake of [^3^H]‐MPP^+^ or [^3^H]‐5‐HT by competing transporters. Synaptosomes were preloaded with radiolabelled substrate in Krebs‐phosphate buffer, which consisted of 126 mM NaCl, 2.4 mM KCl, 0.5 mM KH_2_PO_4_, 1.1 mM CaCl_2_, 0.83 mM MgCl_2_, 0.5 mM Na_2_SO_4_, 11.1 mM glucose, 13.7 mM Na_2_HPO_4_, 1 mg·mL^−1^ ascorbic acid and 50 μM pargyline (pH = 7.4) for 1 h (steady state). Assays were initiated by adding 850 μL of preloaded synaptosomes to 150 μL of test drug. Dose–response curves were generated using eight different concentrations of mephedrone, nor‐mephedrone or 4‐OH‐mephedrone. Assays were terminated by vacuum filtration, and retained radioactivity was quantified by liquid scintillation counting.

### Microdialysis in rat nucleus accumbens

*In vivo* microdialysis procedures were carried out as previously described with minor modifications (Baumann *et al*., 2012). Briefly, male rats anaesthetized with sodium pentobarbital (60 mg·kg^−1^, i.p.) received surgically implanted jugular catheters, and intracerebral guide cannulae aimed at the nucleus accumbens (AP +1.6 mm, ML −1.7 mm relative to bregma; −6.2 mm relative to dura) (Paxinos and Watson, 2007). After a 7–10 day recovery, each rat was placed into a chamber equipped with photobeams for the detection of motor parameters (TruScan, Harvard Apparatus, Holliston, MA, USA) and allowed to acclimatize overnight. Food and water were available *ad libitum* during the acclimatization period. On the following morning, catheters were attached to PE 50 extension tubes, and 0.5 × 2 mm microdialysis probes (CMA/12, Harvard Apparatus, Holliston, MA, USA) were inserted into the guide cannulae. Ringers' solution (150 mM NaCl, 2.8 mM KCl and 2.0 mM CaCl_2_) was perfused through the probes at 0.6 μL·min^−1^ for 3 h. To commence experiments, dialysate samples (20 μL) were collected at 20 min intervals, and drug or saline treatments were given after three baseline samples were obtained. Rats received two sequential i.v. injections of mephedrone or its metabolites, with 1 mg·kg^−1^ administered at time zero, followed by 3 mg·kg^−1^ 60 min later. Saline was administered using the same schedule in a separate group of rats. Dialysate concentrations of dopamine and 5‐HT were quantified using HPLC coupled to electrochemical detection (Baumann *et al*., 2012). Chromatographic data were exported to an Empower software system (Waters, Inc., Milford, MA, USA) for peak identification, integration and analysis.

Correct probe placements were assessed after the microdialysis experiments. Rats were killed by CO_2_ narcosis then decapitated. Brains were quickly removed and immersion fixed in 10% paraformaldehyde for 1 week. Subsequently, brains were sectioned on a cryostat, and the location of each probe tip was verified by inspection of photographic images of the brain taken with a digital camera using the macro lens setting.

### Analysis

Calculations were performed using Microsoft Excel® 2010 (Microsoft Corporation, Redmond, WA, USA) and graphpad prism 5.0. (GraphPad Software Inc., La Jolla, CA, USA). IC_50_ values for uptake inhibition and EC_50_ values for release were determined by nonlinear regression fits. Release data expressed as AUC were analysed by one‐way ANOVA followed by Bonferroni's multiple comparison test. Microdialysis and locomotor data were analysed by two‐way ANOVA (drug treatment × time) followed by Bonferroni's test. The effect of monensin treatment on basal efflux of tritiated substrate was analysed with the Mann–Whitney test. *P* values less than 0.05 (i.e. *P* < 0.05) were considered significant. The data and statistical analysis comply with the recommendations on experimental design and analysis in pharmacology (Curtis *et al*., 2015).

### Materials

2‐Methylamino‐1‐(*p*‐tolyl)propan‐1‐one hydrochloride (mephedrone, MW: 213.70), 2‐amino‐1‐(*p*‐tolyl)propan‐1‐one hydrochloride (nor‐mephedrone, MW: 199.68) and 1‐(4‐(hydroxymethyl)phenyl)‐2‐(methylamino)propan‐1‐one hydrochloride (4‐OH‐mephedrone, MW: 229.70) were synthesized as racemic mixtures. In the case of 2‐(methylamino)‐1‐(*p*‐tolyl)propan‐1‐ol hydrochloride (dihydromephedrone, MW: 215.72), all four stereoisomers [syn‐(1*R*,2*R*), syn‐(1*S*,2*S*), anti‐(1*R*,2*S*) and anti‐(1*S*,2*R*)] were synthesized in their enantiopure form (99%) and tested as 1:1:1:1 mixture. Synthetic procedures and chemical characterization data are given in detail in the Supporting Information. Reagents used in the experiments for uptake inhibition and release in HEK293 cells were used as mentioned in Hofmaier *et al*., 2014. Plasmids encoding human SERT were a generous gift of Dr Randy D. Blakely. For uptake and release experiments in HEK293‐cells and rat brain synaptosomes, [^3^H]‐1‐methyl‐4‐phenylpyridinium ([^3^H]‐MPP^+^; 80–85 μCi mmol^−1^) and [^3^H]‐5‐HT (28.3 μCi mmol^−1^) were purchased from American Radiolabeled Chemicals (St. Louis, MO, USA) and Perkin Elmer (Boston, MA, USA) respectively. All other chemicals and cell culture supplies were from Sigma‐Aldrich (St. Louis, MO, USA) with the exception of cell culture dishes, which were obtained from Sarstedt (Nuembrecht, Germany).

## Results

### Mephedrone metabolites inhibit transporter‐mediated uptake in HEK293 cells

**Figure 2:**
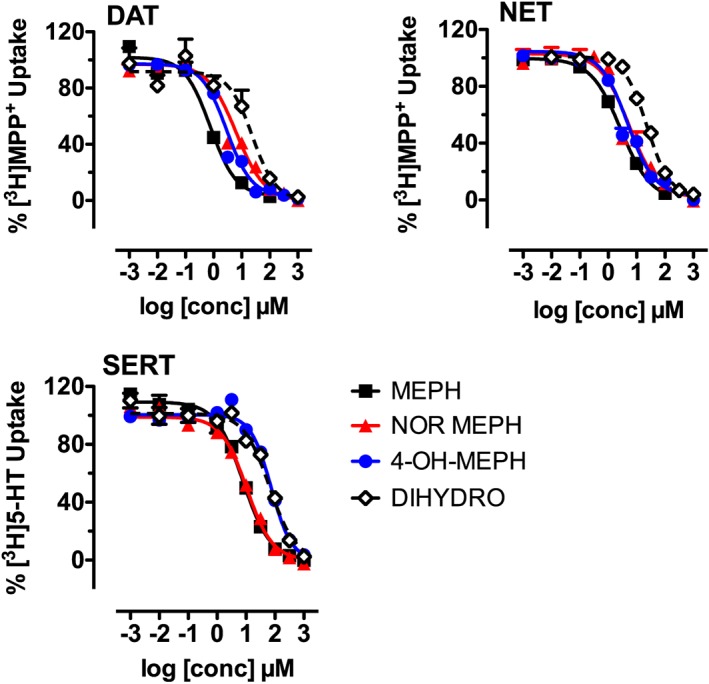
Effects of mephedrone (MEPH), nor‐mephedrone (NOR‐MEPH), 4‐OH‐mephedrone (4‐OH‐MEPH) and dihydromephedrone (DIHYDRO) on transporter‐mediated uptake in HEK293 cells expressing hDAT, hNET and hSERT. Uptake of [^3^H]‐MPP^+^ via hDAT and hNET, and uptake of [^3^H]‐5‐HT by hSERT, was performed as described in Methods; all symbols represent mean values ± SEM, and the numbers in parentheses indicate the number of individual experiments performed in triplicate: hDAT: MEPH (3), NOR‐MEPH (4), 4‐OH‐MEPH (4), DIHYDRO‐MEPH (3); hNET: MEPH (4), NOR‐MEPH (4), 4‐OH‐MEPH (3), DIHYDRO‐MEPH (4); hSERT: MEPH (3), NOR‐MEPH (3), 4‐OH‐MEPH (3), DIHYDRO‐MEPH (3).

We first tested the effects of mephedrone and its metabolites on transporter‐mediated uptake. Figure 2 shows that mephedrone, nor‐mephedrone, 4‐OH‐mephedrone and dihydromephedrone were fully efficacious inhibitors of uptake in HEK293 cells stably expressing hDAT, hNET and hSERT. The potency of nor‐mephedrone and 4‐OH‐mephedrone to inhibit [^3^H]‐MPP^+^ uptake via hDAT and hNET was comparable with mephedrone, with IC_50_ values in the low micromolar range, from 0.7 to 6 μM. The IC_50_ values for dihydromephedrone to inhibit uptake via hDAT and hNET were much weaker (i.e. 24 μM). Uptake inhibition experiments carried out with hSERT‐expressing cells revealed that nor‐mephedrone inhibited uptake in the low micromolar range with an IC_50_ value of 10.6 μM, whereas 4‐OH‐ and dihydromephedrone were much less active with IC_50_ values exceeding 60 μM. The obtained IC_50_ values are shown in Table 1.

**Table 1:** IC_50_ values of test drugs on uptake mediated by hDAT, hNET and hSERT, stably expressed in HEK293 cells

	IC_50_ (μM)		
	DAT	NET	SERT
Mephedrone	0.77 (0.53–1.08)	2.77 (1.92–3.97)	7.83 (6.32–9.75)
Nor‐mephedrone	6.35 (4.66–8.64)	5.46 (3.58–8.31)	10.61 (9.06–12.43)
4‐OH‐mephedrone	2.92 (2.35–3.6)	4.85 (3.28–7.17)	73.53 (62.5–86.51)
Dihydromephedrone	23.97 (8.65–66.46)	23.53 (19.8–27.97)	64.98 (50.66–83.37)

Data are represented as the mean with 95% confidence intervals in parentheses obtained from nonlinear regression fits as shown in Figure 2.

### Mephedrone metabolites induce transporter‐mediated release in HEK293 cells

Data from uptake inhibition assays cannot distinguish whether test drugs act as non‐transported inhibitors or transportable substrates, which evoke release (Scholze *et al*., 2000; Sitte *et al*., 2000; Baumann *et al*., 2013). Therefore, mephedrone and its metabolites were tested in release assays to further explore their interaction with transporters. The release assays were performed with the same transporter‐expressing HEK293 cell lines described above and used a superfusion system (Sitte *et al*., 2000). As described previously, efflux of preloaded [^3^H]‐MPP^+^ or [^3^H]‐5‐HT was monitored in the presence or absence of monensin (10 μM) (Scholze *et al*., 2000). Monensin acts as a selective H^+^/Na^+^ ionophore and dissipates the Na^+^ gradient across cell membranes (Mollenhauer *et al*., 1990). This compound increases the intracellular Na^+^‐concentration (Chen and Reith, 2004) and thus selectively enhances efflux triggered by transporter substrates. Importantly, only substrate‐induced release will be enhanced by the application of monensin, while the effects of non‐transported inhibitors will remain unchanged (Scholze *et al*., 2000; Baumann *et al*., 2013; Sandtner *et al*., 2016). The superfusion assays performed here are a decisive tool to discriminate between inhibitors and substrates (Scholze *et al*., 2000).

**Figure 3:**
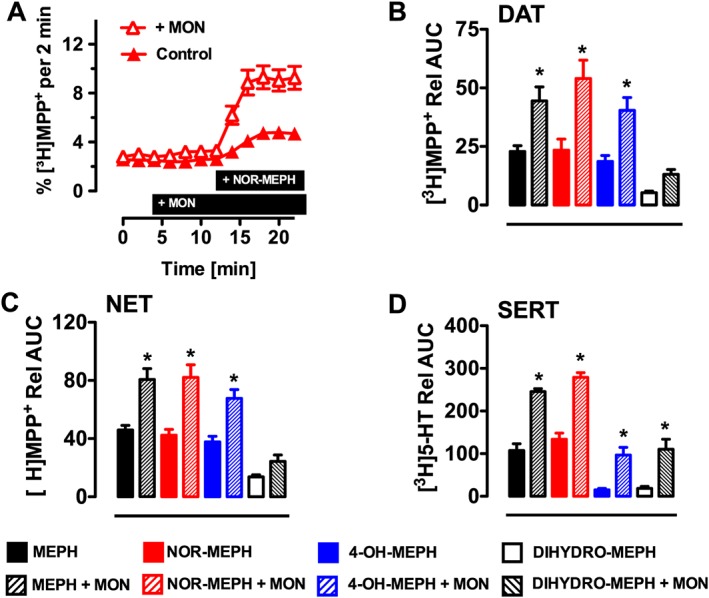
Effects of mephedrone (MEPH), nor‐mephedrone (NOR‐MEPH), 4‐OH‐mephedrone (4‐OH‐MEPH) and dihydromephedrone (DIHYDRO‐MEPH) on transporter‐mediated release of preloaded radiolabelled substrate in HEK293 cells expressing hNET, hDAT and hSERT. [^3^H]‐MPP^+^ was used as the radiolabelled substrate for hDAT and hNET while release by hSERT‐expressing cells was performed using [^3^H]‐5‐HT as the radiolabelled substrate. (A) Representative experiment showing the effect of nor‐mephedrone (10 μM) in the presence or absence of monensin (10 μM) on DAT‐mediated efflux of pre‐loaded [^3^H]‐MPP^+^ (presence of substances indicated by black bar; *n* = 5 independent experiments performed in triplicate). (B–D) For each transporter, AUC was calculated from nine fractions collected after drug treatment (10 μM) in the absence or presence of monensin (MON, 10 μM). Solid bars indicate vehicle + drug, whereas hatched bars indicate MON + drug. Bars represent mean values ± SEM, and the numbers in parentheses indicate the number of individual experiments performed in triplicate: hDAT: MEPH (6), NOR‐MEPH (5), 4‐OH‐MEPH (5), DIHYDRO‐MEPH (5); hNET: MEPH (5), NOR‐MEPH (5), 4‐OH‐MEPH (6), DIHYDRO‐MEPH (5); hSERT: MEPH (5), NOR‐MEPH (5), 4‐OH‐MEPH (5), DIHYDRO‐MEPH (5). *P < 0.05 (Bonferroni's) compared with corresponding vehicle + drug group.

Time‐course experiments with mephedrone and its metabolites (10 μM) demonstrated that all of the agents evoked significant release of preloaded [^3^H]‐MPP^+^ via hDAT and hNET, and release of preloaded [^3^H]‐5‐HT via hSERT. Figure 3A depicts a representative example of time course effects for DAT‐mediated release of [^3^H]‐MPP^+^ induced by nor‐mephedrone in the presence or absence of monensin. It is clear that monensin markedly enhanced the effects of nor‐mephedrone on [^3^H]‐MPP^+^ efflux. Additionally, monensin alone elicited a significant albeit modest increase in substrate release (*P* < 0.05, Mann–Whitney test), in agreement with our previous publications (Scholze *et al*., 2000). As a means to summarize the overall effect of test drugs on release, with and without monensin (10 μM), the data in Figure 3B–D are expressed as AUC for the nine fractions collected after drug treatment. One‐way ANOVA demonstrated that monensin significantly influenced the release of [^3^H]‐MPP^+^ evoked by mephedrone and its metabolites at DAT (*F*_7,91_ = 24.61, *P* < 0.001) and NET (*F*_7,85_ = 14.4, *P* < 0.001). *Post hoc* analysis revealed that enhancement by monensin was significant for mephedrone, nor‐mephedrone and 4‐OH‐mephedrone at hDAT and hNET, but not for dihydromephedrone. One‐way ANOVA demonstrated that monensin significantly augmented the release of [^3^H]‐5‐HT (*F*_7,71_ = 31.68, *P* < 0.001) via hSERT, and this effect was significant for mephedrone and all of its metabolites.

### Mephedrone metabolites induce transporter‐mediated release in synaptosomes

**Figure 4:**
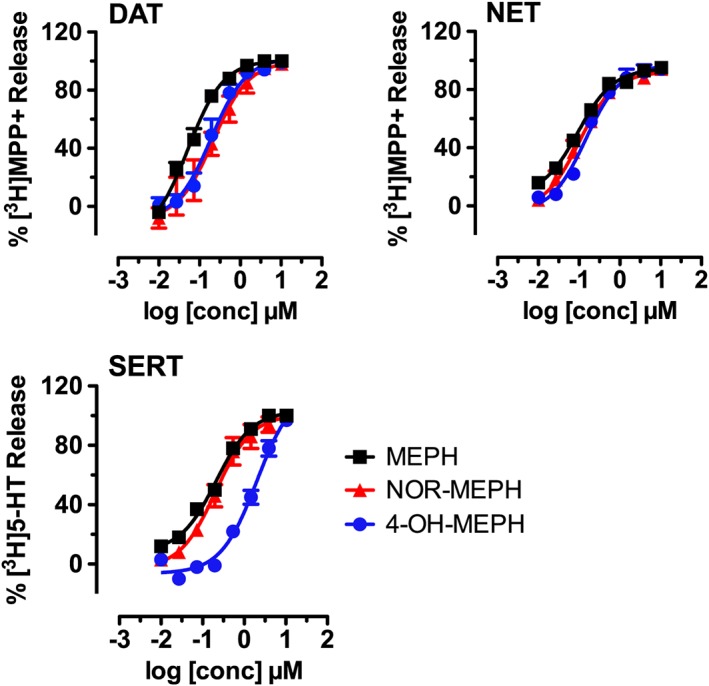
Effects of mephedrone (MEPH), nor‐mephedrone (NOR‐MEPH) and 4‐OH‐mephedrone (4‐OH‐MEPH) on transporter‐mediated release of preloaded radiolabelled substrate in rat brain synaptosomes. [^3^H]‐MPP^+^ was the radiolabelled substrate for DAT and NET assays while [^3^H]‐5‐HT was the radiolabelled substrate for SERT assays. Symbols represent mean values ± SEM obtained from three individual experiments performed in triplicate.

Next, we examined the effects of mephedrone and its metabolites in rat brain synaptosomes to (i) analyse effects of test compounds in a native tissue preparation that contains plasma membrane transporters *in situ* and (ii) compare data from the human and rat transporters. Mephedrone, nor‐ and 4‐OH‐mephedrone were tested in release assays in rat brain synaptosomes, under conditions, which were optimized for each transporter as described previously (Baumann *et al*., 2012). The dose‐effect release data are depicted in Figure 4, and the calculated EC_50_ values are shown in Table 2. In comparison with the parent compound mephedrone, nor‐ and 4‐OH‐mephedrone displayed only slightly reduced potencies as releasers of preloaded [^3^H]‐MPP^+^ at DAT and NET, with EC_50_s ranging from 0.05 μM to 0.22 μM (Figure 4 and Table 2). At SERT, nor‐mephedrone induced release of preloaded [^3^H]‐5‐HT in a manner comparable with mephedrone (EC_50_ = 0.2 μM), whereas a 10‐fold rightward shift was detected for 4‐OH‐mephedrone (EC_50_ = 2 μM).

**Table 2:** EC_50_ values of test drugs on transporter mediated efflux obtained in rat brain synaptosomes

	EC_50_ (μM)		
	DAT	NET	SERT
Mephedrone	0.052 (0.036–0.075)	0.09 (0.08–0.11)	0.21 (0.17–0.26)
Nor‐mephedrone	0.22 (0.14–0.32)	0.1 (0.08–0.13)	0.21 (0.13–0.32)
4‐OH‐mephedrone	0.19 (0.13–0.267)	0.15 (0.11–0.19)	2.01 (1.390–2.91)

Data are represented as the mean and 95% confidence intervals in brackets obtained from nonlinear regression fits as shown in Figure 4.

### Nor‐mephedrone, but not 4‐OH‐mephedrone, affects neurochemistry and behaviour *in vivo*

**Figure 5:**
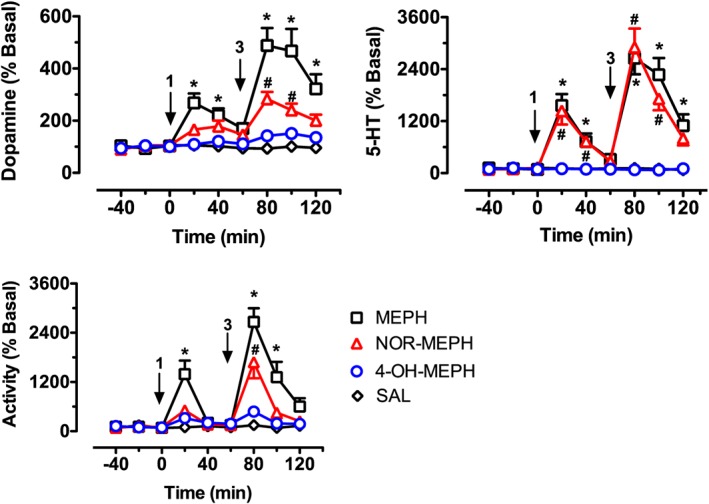
Effects of i.v. administration of mephedrone (MEPH), nor‐mephedrone (NOR‐MEPH) and 4‐OH‐mephedrone (4‐OH‐MEPH) or saline (SAL) on neurochemistry and behaviour in rats undergoing microdialysis in the nucleus accumbens. Drugs were administered i.v. at 1 mg·kg^−1^ at time zero, followed by 3 mg·kg^−1^ 60 min later. Dopamine and 5‐HT were detected by HPLC‐EC as described in Methods. Forward locomotion (activity) was determined by photo‐beam breaks. Data are presented as mean ± SEM, *n* = 6 rats in the control group (SAL) and *n* = 7 rats for all other groups (MEPH, NOR‐MEPH and 4‐OH‐MEPH), arrows indicate time of drug administration. Individual symbols represent significant differences from saline‐treated control at corresponding time points (*P* < 0.05; Bonferroni's): * denotes significance of MEPH compared to saline, and ^#^ denotes significance of NOR‐MEPH compared to saline.

The findings from human and rat transporters agreed that mephedrone, nor‐mephedrone and 4‐OH‐mephedrone were potent substrates at monoamine transporters. Thus, we sought to examine the neurochemical effects of these three compounds *in vivo*. Specifically, extracellular concentrations of dopamine and 5‐HT were assessed by microdialysis in the nucleus accumbens of freely‐moving rats. As depicted in Figure 5, application of two‐way ANOVA (drug treatment × time) demonstrated that drug treatments significantly influenced dialysate concentrations of dopamine (*F*_3,24_ = 63.22, *P* < 0.001) and 5‐HT (*F*_3,24_ = 83.83, *P* < 0.001). *Post hoc* tests revealed that mephedrone increased dopamine after 1 mg·kg^−1^, whereas mephedrone and nor‐mephedrone both elevated dopamine after 3 mg·kg^−1^. 4‐OH‐mephedrone had no significant impact on dopamine at either dose tested. Mephedrone and nor‐mephedrone elevated dialysate concentrations of 5‐HT in a nearly identical manner, with increases of 15‐fold and 25‐fold above baseline for the 1 and 3 mg·kg^−1^ doses respectively. Finally, drug treatments significantly affected motor behaviour (*F*_3,24_ = 36.05, *P* < 0.001) such that mephedrone and nor‐mephedrone increased activity whereas 4‐OH‐mephedrone did not. Mephedrone was more potent than nor‐mephedrone as a locomotor stimulant, but both compounds significantly stimulated motor activity at the 3 mg·kg^−1^ dose.

## Discussion

The aim of the present study was to determine the pharmacological effects of phase I metabolites of mephedrone and decipher their precise mode of action at monoamine transporters. The synthetic cathinone mephedrone has been shown to act as a non‐selective, amphetamine‐like substrate at monoamine transporters, thereby triggering release of dopamine, noradrenaline and 5‐HT into the extracellular space (Baumann *et al*., 2012; Eshleman *et al*., 2013; Simmler *et al*., 2013). The neurochemical effects of mephedrone mimic those of MDMA (Kehr *et al*., 2011; Baumann *et al*., 2012; Wright *et al*., 2012), but mephedrone has a number of distinct pharmacological effects when compared with MDMA and other ring‐substituted amphetamines (reviewed by Green *et al*., 2014). Many therapeutic and abused stimulant drugs – including diethylpropion, phendimetrazine and MDMA – are transformed by hepatic mechanisms into bioactive metabolites (Yu *et al*., 2000; Rothman *et al*., 2002; Green *et al*., 2003). To examine whether metabolites of mephedrone might be bioactive, we tested the known metabolites nor‐mephedrone, 4‐OH‐mephedrone and dihydromephedrone for their interactions with DAT, NET and SERT. It was found that all of the metabolites acted as substrate‐type releasers, but nor‐mephedrone and 4‐OH‐mephedrone were much more potent than dihydromephedrone in this regard. Importantly, only nor‐mephedrone influenced brain neurochemistry and behaviour upon systemic administration.

The present *in vitro* data from HEK293 cells show that mephedrone metabolites inhibit uptake in a concentration‐dependent manner at all three human plasma membrane monoamine transporters. Nor‐mephedrone and 4‐OH‐mephedrone inhibited uptake at hDAT and hNET with potency comparable with mephedrone, whereas dihydromephedrone was much weaker. Uptake inhibition assays can identify compounds that interact with monoamine transporters, but cannot discriminate whether such compounds act as inhibitors or substrates. Thus, we tested the effects of mephedrone metabolites using release assays in HEK293 cells and rat brain synaptosomes. Nor‐mephedrone and 4‐OH‐mephedrone evoked release of radiolabelled substrates from HEK293 cells stably expressing hDAT, hNET or hSERT. The releasing action of the drugs was augmented in the presence of monensin, an ionophore that dissipates Na^+^ gradients across plasma membranes. The enhancement of release by monensin provides crucial mechanistic evidence that mephedrone and its metabolites function mainly as transporter substrates, not merely as inhibitors and thus are capable of inducing release of monoamines via their cognate transporters. Consistent with the data in HEK293 cells, nor‐mephedrone and 4‐OH‐mephedrone induced release of [^3^H]‐MPP^+^ via DAT and NET, and release of [^3^H]‐5‐HT via SERT, in rat brain synaptosomes. Our findings with nor‐mephedrone in synaptosomes agree with the recent findings of Hutsell *et al*. (2016) who reported that stereoisomers of nor‐mephedrone (i.e. stereoisomers of 4‐methylcathinone) are non‐selective transporter substrates that evoke neurotransmitter release from synaptosomes *in vitro.*

Previous investigations have revealed that the corresponding IC_50_ and EC_50_ values for a given drug to inhibit uptake or induce release may differ several‐fold (Scholze *et al*., 2000; Sitte *et al*., 2001). The apparent differences in potency that we observed here for inhibition of uptake (IC_50_ values in the μM range) versus stimulation of release (EC_50_ values in the nM range) might be attributed to different assay systems and methods used in our studies. For example, uptake assays in HEK293 cells use static incubation conditions, while release assays in HEK293 cells use dynamic perfusion conditions. Comparing results from release assays with HEK293 cells versus rat brain synaptosomes is even more problematic because the latter preparation consists of homogenized tissue that maximizes the surface area for drug‐protein interactions. Additionally, HEK293 cells are non‐neuronal in origin and do not possess all critical components of the plasma membrane protein machinery that are present in neurons *in vivo*. Despite the different assay systems and methods employed here, all of the findings agree that mephedrone and its metabolites are substrates at monoamine transporters.

Even though the mephedrone metabolites tested acted as transporter substrates *in vitro*, only nor‐mephedrone significantly affected neurochemistry and behaviour *in vivo*. The neurochemical profile of nor‐mephedrone closely resembled that of mephedrone at the doses tested in our study, but nor‐mephedrone had weaker effects on extracellular dopamine and locomotion. Thus, it seems nor‐mephedrone displays a more serotonergic profile of activity than the parent compound mephedrone. The reduced locomotor response to nor‐mephedrone as compared with mephedrone is probably linked to blunted dopaminergic effects of the metabolite, because previous studies have shown that extracellular dopamine levels in the nucleus accumbens are tightly correlated with the extent of motor activation produced by stimulant drugs (Zolkowska *et al*., 2009; Baumann *et al*., 2011). Surprisingly, the systemic administration of 4‐OH‐mephedrone had no significant effect on extracellular neurotransmitters in the brain or on behaviour. Taken together with the *in vitro* findings, our *in vivo* data with 4‐OH‐mephedrone suggest this metabolite may not penetrate through the blood–brain barrier. The likelihood of substances to enter the brain is correlated with their size and lipid solubility (van Bree *et al*., 1988; Waterhouse, 2003). Distribution coefficients calculated for mephedrone and its metabolites indicate a clear‐cut separation of lipohilic mephedrone and nor‐mephedrone on the one hand (logD7.4 = 1.39 and 1.29, respectively) and hydrophilic 4‐OH‐mephedrone on the other hand (logD7.4 = 0.14). As a consequence, the increased hydrophilicity of 4‐OH‐mephedrone, as compared with mephedrone and nor‐mephedrone, likely precludes the hydroxylated metabolite from entering the brain. We have noted a similar situation to the hydroxylated metabolites of MDMA, which are devoid of central activity when administered systemically to rats (Schindler *et al*., 2014). Nevertheless, there is increasing evidence that points to the presence of various CYPs in brain tissue. Even though the expression levels of CYPs in the brain are low when compared with those in liver (Miksys and Tyndale, 2002), it is interesting to speculate that *in situ* metabolism of mephedrone and formation of phase 1 metabolites in brain could impact on mephedrone action *in vivo*. For instance, CYP2D6 has been detected in various regions of human brain, including substantia nigra and hippocampus (Siegle *et al*., 2001). As a consequence, mephedrone metabolites could be formed in close proximity to monoamine transporters and thereby contribute to the effects of mephedrone. At present, there is no evidence that formation of metabolites in the central nervous system is of any pharmacological relevance. Interestingly, the dihydroxy metabolite of MDMA, 3,4‐dihydroxymethamphetamine, displays potent stimulatory effects on heart rate and blood pressure upon systemic administration (Schindler *et al*., 2014). Our results suggest that future investigations should examine the possible cardiovascular effects of 4‐OH‐MEPH. The most abundant mephedrone metabolite detected in blood from forensic traffic cases was 4‐OH‐MEPH (Pedersen *et al*., 2013). In two cases, the blood concentrations of parent drug:metabolite were 28:2 and 29:9 μg/kg. In a number of cases, trace amounts of NOR‐MEPH and DIHYDRO‐MEPH were also detected in blood and urine samples, but 4‐OH‐MEPH was highest in urine, highlighting its hydrophilicity.

The present data alone cannot clarify whether nor‐mephedrone contributes to the psychoactive properties of systemically administered mephedrone in animals or humans. Further studies are needed to determine the blood and brain concentrations of nor‐mephedrone after mephedrone exposure. It is noteworthy that nor‐mephedrone is the most abundant metabolite of mephedrone identified in rats (Khreit *et al*., 2013; Martinez‐Clemente *et al*., 2013) whereas 4‐OH‐mephedrone is the major metabolite in humans (Pedersen *et al*., 2013; Pozo *et al*., 2015). Currently, no information is available on the pharmacokinetics and bioavailability of the metabolites after mephedrone administration in either species. The collective results presented here demonstrate that phase I metabolites of mephedrone are non‐selective transporter substrates at DAT, NET and SERT, similar to the parent compound. However, only nor‐mephedrone affects neurochemistry and behaviour when administered peripherally, suggesting this metabolite could contribute significantly to the unique profile of psychoactive effects produced by mephedrone. Further studies are warranted to examine this intriguing hypothesis.

## Author contributions

F.P.M., O.D‐C., J.S.P., L.W. and N.B. performed all experimental work. F.P.M., L.W., M.D.M., M.H.B. and H.H.S. designed the experiments. F.P.M., M.H.B. and H.H.S. wrote the manuscript and received significant input from all other authors.

## Conflict of interest

H.H.S. has received honoraria for lectures and consulting from AbbVie, Lundbeck, MSD, Pfizer, Ratiopharm, Roche, Sanofi‐Aventis and Serumwerk Bernburg (past 5 years). All other authors declare no conflicts of interest.

## Declaration of transparency and scientific rigour

This Declaration acknowledges that this paper adheres to the principles for transparent reporting and scientific rigour of preclinical research recommended by funding agencies, publishers and other organisations engaged with supporting research.

## Supporting information


**Figure S1** Unless noted otherwise, all reagents were purchased from commercial suppliers and used without further purification.
