# Supplementary material for: Phase I metabolites of mephedrone display biological activity as substrates at monoamine transporters
Source: Br J Pharmacol. 2016 Jul 31;173(17):2657–68. doi: 10.1111/bph.13547 (PMC4978154; doi:10.1111/bph.13547)
Supplement: Supplementary file 1 — Figure S1 Unless noted otherwise, all reagents were purchased from commercial suppliers and used without further purification. [file BPH-173-2657-s001.pdf]

Supporting Information

General

Unless noted otherwise, all reagents were purchased from commercial suppliers and used without further purification. DCM, Et_2O, dioxane, MeOH, THF and toluene intended for water-free reactions were pre-distilled and then desiccated on Al_2O_3 columns (PURESOLV, Innovative Technology). Chromatography solvents were distilled prior to use. For all other solvents quality grade is given in the reaction procedures. Column chromatography was performed on a Büchi Sepacore Flash System (2 x Büchi Pump Module C-605, Büchi Pump Manager C-615, Büchi UV Photometer C-635, Büchi Fraction Collector C-660) or standard manual glass columns using silica gel from Merck (40-63 µm) using LP and Et_2O or EtOAc mixtures. Desiccation of organic solvents after extraction in reaction workup was performed using anhydrous sodium sulfate or magnesium sulfate and subsequent filtration. NMR spectra were recorded from $CDCl_3$, D_2O or d_6-DMSO solutions on a Bruker AC 200 (200 MHz) or Bruker Avance UltraShield 400 (400 MHz) spectrometer and chemical shifts are reported in ppm using tetramethylsilane as an internal standard. Whenever possible calibration *via* residual solvent peaks was performed. Enantiomeric excess was determined *via* HPLC using a ChiralPak AS-H (250 mm x 4.6 mm ID), IA (250 mm x 4.6 mm ID) or IB (250 mm x 4.6 mm ID) column on a Thermo Scientific/Dionex Ulitmate 3000 HPLC using mixtures of n-heptane/*i*-PrOH 1 - 20% as mobile phase. Basic analytes were eluted using 0.1 vol% triethylamine, for aminoalcohols 0.1 vol% ethanolamine was used as a modifier. Specific rotation was measured on an Anton Paar MCP500 polarimeter at the specified conditions.

Analytical protocol for the determination of enantiomeric excess using chiral HPLC

For the analysis of the enantiomeric composition of methcathinone samples on normal-phase chiral HPLC, an analytical protocol from the literature[95] was adapted.

In a screw-cap vial 1.5 mg of the corresponding hydrochloride were dissolved in water (1 mL). Then 10 µl of 2M NaOH were added and the free base was extracted with dichloromethane (1 mL). The organic layer was dried over sodium sulfate, filtered and evaporated in a stream of air. The oily residue was taken up in an HPLC-grade mixture of n-heptane/*i*-PrOH (97:3), filtered and immediately subjected to HPLC analysis.

Synthetic procedures

Synthesis of *rac*-MEPH

2-Chloro-1-(p-tolyl)-1-propanone

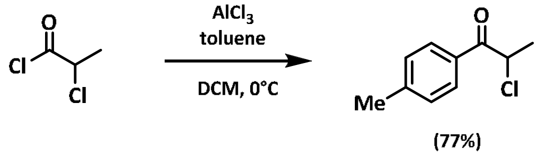

The title compound was prepared according to a modified literature procedure.[97]

Aluminum(III) chloride (9.6 g, 72 mmol, 1.05 equiv.) was suspended in dry DCM (50 mL) and cooled to 0 C. 2-Chloropropionyl chloride (8.7 g, 68 mmol, 1 equiv.) was added slowly via syringe, followed by dropwise addition of toluene (7 g, 76 mmol, 1.1 equiv.). Stirring was continued for 2 hours when TLC (LP/EtOAc 30:1) indicated full conversion.

The reaction mixture was poured onto 200mL of ice/water mixture and extracted with 3x100mL of DCM. The combined organic extracts were washed with $NaHCO_3$ satd. and brine and dried over anhydrous sodium sulfate. The crude product was distilled via Kugelrohr (b.p. 130 °C/0.12mbar) and purified via flash column chromatography (90 g SiO_2, eluent LP/EtOAc 3%).

Spectral data were in agreement with those reported in the literature. [97]

**Yield**	9.58 g (77%), colorless solid
**M.p.**	46 - 48 °C (lit.[145]: 50°C)
**Molecular formula, m.w.**	$C_{10}H_{11}ClO$, 182.65
**^{1}H-NMR (400 MHz, $CDCl_3$)**	δ = 1.74 (d, *J* = 6.7 Hz, 3H), 2.43 (s, 3H), 5.24 (q, *J* = 6.7 Hz, 1H), 7.10 – 7.36 (m, 2H), 7.86 – 7.99 (m, 2H)
**^{13}C-NMR (100 MHz, $CDCl_3$)**	δ = 20.2 (q), 21.8 (q), 52.9 (d), 129.2 (d), 129.6 (d), 131.6 (s), 144.9 (s), 193.4 (s)

*2-Bromo-1-(*p*-tolyl)-1-propanone*

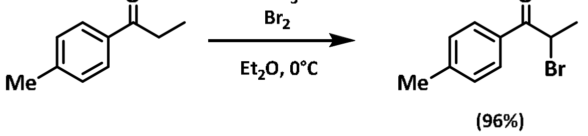

The title compound was prepared according to a modified literature procedure.[148]

Aluminum(III) chloride (500 mg, 3.75 mmol, 0.05 equiv.) was added to a solution of 1-(*p*-tolyl)-1-propanone (11.1 g, 75 mmol, 1 equiv.) in anhydrous Et_2O (110 mL) under argon at 0 °C . Bromine (3.84 mL, 75 mmol, 1 equiv.) was added as slowly as it was consumed as judged by the color of the

reaction mixture. After 10 minutes the reaction rate noticeably increased and the remaining bromine was added within minutes. Stirring continued at 0 °C for 2 hours. At this point, a second layer which had formed after the bromine addition had dissolved.

The reaction mixture was poured onto 300 mL of satd. $NaHCO_3$ and was extracted with 4 x 100 mL of Et_2O. The pure product was obtained after drying of the organic layer over anhydrous $MgSO_4$ and evaporation of the solvent.

Spectral data were in agreement with those reported in the literature. [149]

**Yield**	16.40 g (96%), colorless oil
**Molecular formula, m.w.**	$C_{10}H_{11}BrO$, 227.10
**^{1}H NMR (400 MHz, $CDCl_3$)**	δ = 1.88 (d, *J* = 6.6 Hz, 3H), 2.41 (s, 3H), 5.28 (q, *J* = 6.6 Hz, 1H), 7.24 – 7.31 (m, 2H), 7.88 – 7.95 (m, 2H)
**^{13}C NMR (101 MHz, $CDCl_3$)**	δ = 20.3 (q), 21.8 (q), 41.7 (d), 129.1 (d), 129.5 (d), 131.6 (s), 144.7 (s), 193.1 (s)

(rac)-2-Methylamino-1-(p-tolyl)propan-1-one hydrochloride

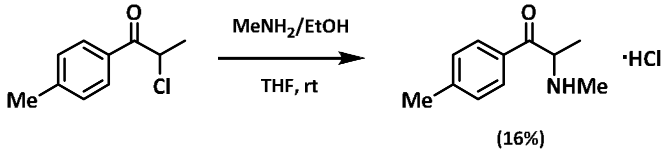

The title compound was prepared according to a modified literature procedure.[36]

To a solution of 2-chloro-1-(*p*-tolyl)propan-1-one (500 mg, 2.74 mmol, 1 equiv.) in THF (14 mL) $MeNH_2$ (33% in EtOH, 1.03 mL, 11 mmol, 4 equiv) was added and stirred at rt for 24h. A white precipitate was centrifuged off. Because TLC analysis showed residual starting material, another equivalent of $MeNH_2$ (0.25 mL, 2.75 mmol) was added and stirring continued for 24 hours. According to TLC conversion was still incomplete. A second fraction of precipitate was obtained by centrifuging.

In order to remove an impurity of methylamine hydrochloride, the combined precipitates were extracted three times with a small quantity of chloroform, filtered and reprecipitated by addition of acetone.

**Yield**	95 mg (16%), colorless solid
**Molecular formula, m.w.**	$C_{11}H_{16}ClNO$, 213.71
**M.p.**	219 - 224 °C
**^{1}H NMR (400 MHz, D_2O)**	δ 1.60 (d, *J* = 7.3 Hz, 3H), 2.44 (s, 3H), 2.80 (s, 3H), 5.07 (q, *J* = 7.3 Hz, 1H), 7.45 (d, *J* = 8.1 Hz, 2H), 7.92 (d, *J* = 8.2 Hz, 2H)
**^{13}C NMR (101 MHz, D_2O)**	δ 15.4 (q), 20.9 (q), 30.9 (q), 59.5 (d), 129.0 (d), 129.6 (s), 129.9 (d), 147.4 (s), 197.1 (s)
**HR-ESI-MS**	m/z 178.1236 $[M+H]^+$ (calcd 178.1233, diff –5.57ppm)

Synthesis of *rac*-NOR-MEPH

Di-tert-butyl-iminodicarboxylate potassium salt

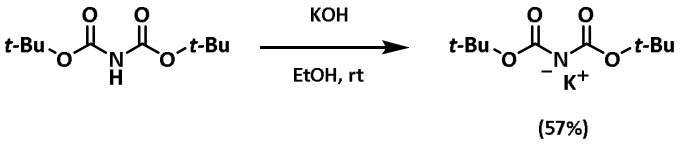

The title compound was prepared according to a modified literature procedure.[147]

A solution of potassium hydroxide (1.2 g, 20.7 mmol, 1 equiv.) in 10 mL of EtOH was added to a solution of di-*tert*-butyl-iminodicarboxylate (4.5 g, 20.7 mmol, 1 equiv.) in 10 mL of EtOH. The reaction mixture was stirred at rt for 20 minutes.

Then, Et_2O (20 mL) was added, the precipitate was collected by filtration and dried *in vacuo*.

**Yield**	3.02 g (57%), colorless solid
**M.p.**	> 185 °C (decomposition)
**Molecular formula, m.w.**	$C_{10}H_{18}KNO_4$, 255.36
**^{1}H NMR (400 MHz, $CDCl_3$)**	δ 1.29 (s, 18H)
**^{13}C NMR (101 MHz, $CDCl_3$)**	δ 28.5 (q), 74.0 (s), 160.4 (s)

(rac)-2-(N,N-di-Boc-imino)-1-(p-tolyl)propan-1-one

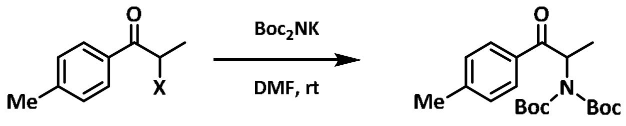

From the 2-chloropropiophenone precursor

Potassium bis(*tert*-butyloxycarbonyl)imide (613 mg, 2.4 mmol, 1.2 equiv.) was added to a solution of 2-chloro-1-(*p*-tolyl)-1-propanone (356 mg, 2.0 mmol, 1 equiv.) in DMF (4 mL) and stirred at rt. After 24 hours only traces of residual starting material could be detected by TLC.

The reaction was diluted with 50 mL of water and extracted with 3 x 30 mL of MTBE. The organic layer was washed with brine and dried over anhydrous $MgSO_4$. Column chromatography (50 g SiO_2, LP/EtOAc 6 - 12%) yielded the product as colorless solid.

Yield 306 mg (42%), colorless solid

From the 2-bromopropiophenone precursor

Potassium bis(*tert*-butyloxycarbonyl)imide (2.7 g, 10.57 mmol, 1.2 equiv.) was added to a solution of 2-bromo-1-(*p*-tolyl)-1-propanone (2.0 g, 8.82 mmol, 1 equiv.) in DMF (17.5 mL) and stirred at rt overnight.

The reaction was diluted with 100 mL of water and extracted with 4 x 50 mL of MTBE. The organic layer was washed with brine and dried over anhydrous $MgSO_4$. Column chromatography (90 g SiO_2, LP/EtOAc 8%) yielded the product as colorless solid.

Yield 1.78 g (56%), colorless solid

**Molecular formula, m.w.**	$C_{20}H_{29}NO_5$, 363.45
**M.p.**	86 - 88 °C
**^{1}H NMR (400 MHz, $CDCl_3$)**	δ = 1.41 (s, 18H), 1.52 (d, *J* = 6.6 Hz, 3H), 2.37 (s, 3H), 5.45 (q, *J* = 6.6 Hz, 1H), 7.15 – 7.22 (m, 2H), 7.61 – 7.68 (m, 2H)
**^{13}C NMR (101 MHz, $CDCl_3$)**	δ = 14.9 (q), 21.7 (q), 28.0 (q), 57.7 (d), 83.5 (s), 128.1 (d), 129.1 (d), 133.6 (s), 143.0 (s), 151.8 (s), 197.7 (s)
**HR-ESI-MS**	m/z 386.1959 $[M+Na]^+$ (calcd 386.1943, diff –5.55ppm)

*(*rac*)-2-Amino-1-(*p*-tolyl)propan-1-one hydrochloride*

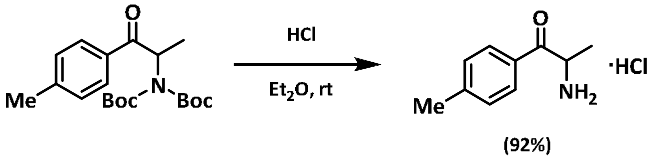

2-(*N,N*-di-Boc-imino)-1-(*p*-tolyl)propan-1-one (150 mg, 0.41 mmol, 1 equiv.) was added to anhydrous HCl/Et_2O (2 M, 4 mL) and stirred at rt for 16 hours.
The solvent was evaporated to dryness in a stream of air. The residue was taken up in 2 x 2 mL of water, filtered, evaporated and dried in high vacuum.

**Yield**	76 mg (92%), colorless solid
**Molecular formula, m.w.**	$C_{10}H_{14}ClNO$, 199.68
**M.p.**	195 - 210 °C (purity > 95% according to NMR), lit.[150]: 192 - 193 °C)
**^{1}H NMR (400 MHz, D_2O)**	δ = 1.59 (d, *J* = 7.3 Hz, 3H), 2.44 (s, 3H), 5.17 (q, *J* = 7.3 Hz, 1H), 7.44 (d, *J* = 8.1 Hz, 2H), 7.92 (d, *J* = 8.2 Hz, 2H)
**^{13}C NMR (101 MHz, D_2O)**	δ = 16.8 (q), 21.0 (q), 51.8 (d), 129.0 (d), 129.6 (s), 129.9 (d), 147.1 (s), 197.6 (s)
**HR-ESI-MS**	m/z 164.1078 $[M+H]^+$ (calcd 164.1077, diff –5.44 ppm)

Synthesis of *rac*-4-OH-MEPH

4'-(Bromomethyl)propiophenone

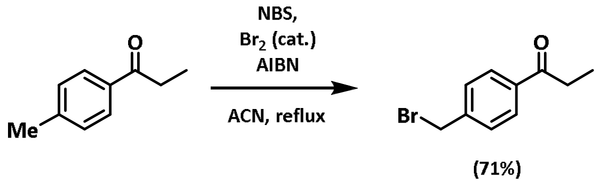

The title compound was prepared according to a modified literature procedure.[98]

A mixture of 4'-methylpropiophenone (11.0 g, 75 mmol, 1 equiv.), colorless *N*-bromosuccinimide (14.6 g, 82 mmol, 1.1 equiv.), AIBN (1.22 g, 10 mol%) and bromine (3 drops) in dry acetonitrile was heated to reflux. After 3 hours additional AIBN (0.6 g) was added. After 6 hours TLC analysis indicated full conversion.

The reaction mixture was diluted with EtOAc and washed successively with 5% sodium thiosulfate, 0.5 M HCl, $NaHCO_3$ satd. and brine, dried over anhydrous sodium sulfate and evaporated. The crude product was crystallized from methanol followed by Kugelrohr distillation (b.p. 150 °C/0.01 mbar) to give 5.86 g of pure product. A second fraction was obtained from the mother liquor via Kugelrohr distillation (6.09 g).

Spectral data were in agreement with those reported in the literature.[98]

**Yield**	11.95 g (71%), colorless solid
**Molecular formula, m.w.**	$C_{10}H_{11}BrO$, 227.10
**M.p.**	59 - 61 °C (lit.[98]: 57 - 58 °C)
**^{1}H-NMR (200 MHz, $CDCl_3$)**	δ = 1.21 (t, *J* = 7.3, 2.0 Hz, 3H), 2.98 (q, *J* = 7.2 Hz, 2H), 4.49 (s, 2H), 7.43 – 7.49 (m, 2H), 7.89 – 7.95 (m, 2H)
**^{13}C-NMR (50 MHz, $CDCl_3$)**	δ = 8.3 (q), 32.0 (t), 32.3 (t), 128.6 (d), 129.3 (d), 136.8 (s), 142.6 (s), 200.1 (s)

4-Propionylbenzyl acetate

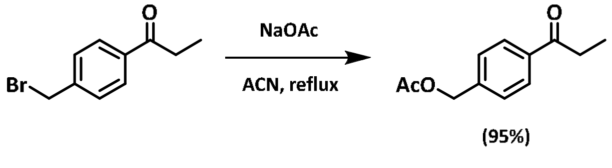

The title compound was prepared according to a modified literature procedure.[98]

A mixture of 4'-(bromomethyl)propiophenone (5.0 g, 22 mmol, 1 equiv.) and NaOAc·$3H_2O$ (6 g, 44 mmol, 2 equiv.) in acetonitrile was heated to reflux for 20 hours. Since conversion was still incomplete, more sodium acetate (3 g, 22 mmol, 1 equiv.) was added and the reaction was heated to reflux for an additional 24 hours. TLC indicated full consumption of the starting material (LP/EtOAc 4:1).

The reaction was diluted with EtOAc (100 mL) and washed with 2 x 50 mL of water, brine and dried over anhydrous sodium sulfate. Evaporation of the solvent provided the pure product.

Spectral data were in agreement with those reported in the literature. [98]

**Yield**	4.33 g (95%), colorless oil
**Molecular formula, m.w.**	$C_{12}H_{14}O_3$, 206.24
**^{1}H-NMR (400 MHz, CDCl$_3$)**	δ = 1.21 (t, *J* = 7.2 Hz, 3H), 2.12 (s, 3H), 2.99 (q, *J* = 7.2 Hz, 2H), 5.14 (s, 2H), 7.39 – 7.47 (m, 2H), 7.92 – 7.98 (m, 2H)
**^{13}C-NMR (100 MHz, CDCl$_3$)**	δ = 8.2 (q), 20.9 (q), 31.9 (t), 65.5 (t), 127.9 (d), 128.3 (d), 136.6 (s), 140.9 (s), 170.7 (s), 200.4 (s)

4'-(Hydroxymethyl)propiophenone

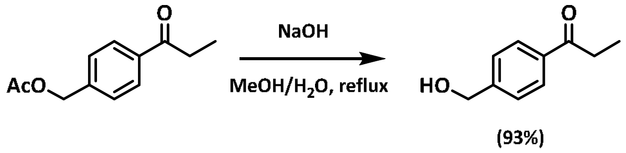

The title compound was prepared according to a modified literature procedure.[98]

4-Propionylbenzyl acetate (4.54 g, 22 mmol, 1 equiv.) was dissolved in methanol (5 mL). A solution of sodium hydroxide (1.76 g, 44 mmol, 2 equiv.) in 2 mL of water was added and the reaction mixture was heated to reflux. Upon addition of the base, the reaction mixture turned red color. After 15 min TLC (LP/EtOAc 4:1) indicated full conversion.

The reaction was cooled to rt, diluted with water (50 mL) and extracted with 5 x 20 mL of Et_2O. The combined organic extracts were dried over anhydrous sodium sulfate and evaporated to give the pure product.

Spectral data were in agreement with those reported in the literature. [98]

**Yield**	3.25 g (90%), colorless solid
**Molecular formula, m.w.**	$C_{10}H_{12}O_2$, 164.20
**M.p.**	51 - 53 °C (lit.[98]: 52 - 53 °C)
**^{1}H NMR (400 MHz, CDCl$_3$)**	δ = 1.20 (t, *J* = 7.3 Hz, 3H), 2.98 (q, *J* = 7.2 Hz, 2H), 4.74 (s, 2H), 7.39 – 7.46 (m, 2H), 7.89 – 7.96 (m, 2H)
**^{13}C NMR (101 MHz, CDCl$_3$)**	δ = 8.4 (q), 31.9 (t), 64.7 (t), 126.7 (d), 128.4 (d), 136.2 (s), 146.2 (s), 200.8 (s)
**HR-ESI-MS**	m/z 165.0921 $[M+H]^+$ (calcd 165.0917, diff –6.81ppm)

2-Bromo-4'-(hydroxymethyl)propiophenone

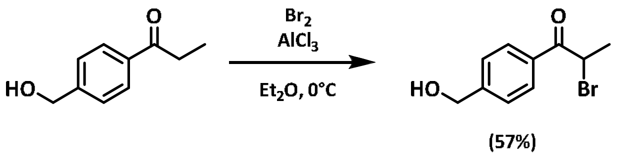

The title compound was prepared according to a modified literature procedure.[148]

4-(Hydroxymethyl)propiophenone (3.25 g, 19.8 mmol, 1 equiv.) was dissolved in dry Et_2O (35 mL) and cooled to 0 °C under argon. A catalytic amount of aluminum(III) chloride (132 mg, 1 mmol, 5 mol%) was added. Then bromine (1.01mL, 19.8mmol, 1 equiv.) was added in small portions (~ 0.2 mL). Before a fresh portion of bromine was added, the reaction was allowed to decolorize (from intensely colored orange to pale yellow). During the course of bromine addition a significant increase in reaction rate was observed, due to autocatalysis by HBr formed. After the addition of bromine was complete, the reaction was allowed to warm to rt and stirring continued for 10min.

The reaction mixture was diluted with $NaHCO_3$ satd. (100 mL) and $Na_2S_2O_3$ satd. (20 mL) and extracted with 5 x 50 mL Et_2O. The combined organic layers were dried over anhydrous sodium sulfate and evaporated. The crude product was purified by flash column chromatography (90g SiO_2, eluent LP/EtOAc 10-15%).

**Yield**	2.90 g (57%), colorless oil
**Molecular formula, m.w.**	$C_{10}H_{11}BrO_2$, 243.10
**^{1}H NMR (400 MHz, $CDCl_3$)**	δ = 1.90 (d, *J* = 6.6 Hz, 3H), 4.78 (s, 2H), 5.29 (q, *J* = 6.6 Hz, 1H), 7.44 – 7.51 (m, 2H), 7.97 – 8.05 (m, 2H)
**^{13}C NMR (101 MHz, $CDCl_3$)**	δ= 20.2 (q), 41.7 (d), 64.7 (t), 126.9 (d), 129.4 (d), 133.3 (s), 147.0 (s), 193.2 (s)

(rac)-1-(4-(Hydroxymethyl)phenyl)-2-(methylamino)propan-1-one hydrochloride

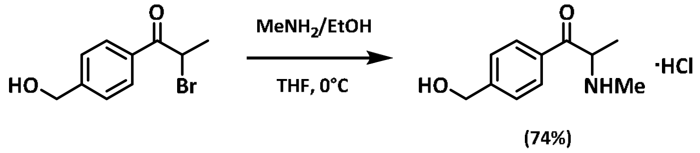

2-Bromo-4'-(hydroxymethyl)propiophenone (300 mg, 1.23 mmol, 1 equiv.) was dissolved in technical-grade THF (6 mL) and a solution of methylamine (33 % in EtOH, 0.46 mL, 4.92 mmol, 4 equiv.) was added. The reaction was stirred at rt for 3 hours when TLC (eluent LP/EtOAc 4:1) indicated full consumption of the starting material.

The reaction mixture was poured onto 2 M HCl (20 mL) and evaporated to dryness (50 °C, 10 mbar). To remove residual water, several portions of toluene were added and evaporated (azeotropic water removal) until a dry powdery residue was obtained. The solid residue was washed with 2 x 2 mL of $CHCl_3$. In order to separate it from remaining methylammonium chloride, the product was extracted with 3 x 5 mL of acetonitrile by heating to reflux, cooling to rt and filtration. Methylammonium chloride remained as insoluble fraction.

**Yield**	195 mg (74%), colorless solid

**Molecular formula, m.w.**	$C_{11}H_{16}ClNO_2$, 229.70
**M.p.**	130 - 136 °C
**^{1}H NMR (400 MHz, D_2O)**	δ = 1.61 (d, *J* = 7.3 Hz, 3H), 2.81 (s, 3H), 4.75 (s, 2H), 5.11 (q, *J* = 7.2 Hz, 1H), 7.58 (d, *J* = 8.1 Hz, 2H), 8.02 (d, *J* = 8.2 Hz, 2H)
**^{13}C NMR (101 MHz, D_2O)**	δ = 15.2 (q), 30.9 (q), 59.6 (d), 63.1 (t), 127.4 (d), 129.3 (d), 131.4 (s), 148.1 (s), 197.2 (s)
**HR-ESI-MS**	m/z 194.1186 $[M+H]^+$ (calcd 194.1182, diff – 4.92ppm)

Enantioselective synthesis of DIHYDRO-MEPH isomers

N-Boc-Alanine

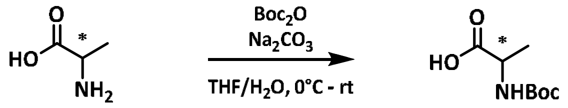

(*S*)- or (*R*)-alanine

(*R*) (74%)
(*S*) (82%)

The title compound was prepared according to a modified literature procedure.[141]

Alanine (4 g, 45 mmol, 1 equiv.) and sodium carbonate (9.5 g, 90 mmol, 2 equiv.) were dissolved in a H_2O/THF mixture (60 mL, 1 : 1). The reaction mixture was cooled to 0 °C and Boc_2O (10.8 g, 50 mmol, 1.1 equiv.) was added. The reaction was allowed to warm to rt and stirred for 48 h.
The reaction mixture was acidified by careful addition of 100 mL of 1M HCl until pH = 2 was reached and extracted with 5 x 80 mL of EtOAc. The combined organic layers were washed with brine, dried over anhydrous magnesium sulfate and evaporated.

N-Boc-L-alanine

**Yield**	6.96 g (82%) colorless oil

***N*-Boc-D-alanine**

**Yield**	6.27 g (74%) colorless oil

**Molecular formula, m.w.**	$C_8H_{15}NO_4$, 189.21
**^{1}H NMR (400 MHz, DMSO-*d*$_6$)**	Mixture of rotamers. δ 1.21 (d, *J* = 7.3 Hz, 3H), 1.30 – 1.44 (m, 9H), 3.77 – 3.97 (m, 1H), 6.66 – 6.74 (m, 1H), 7.06 (d, *J* = 7.5 Hz, 1H), 12.37 (s, 1H)
**^{13}C NMR (101 MHz, DMSO-*d*$_6$)**	δ 17.1 (q), 28.2 (q), 48.8 (d), 77.9 (s), 155.3 (s), 174.7 (s)

Spectral data were in agreement with those reported in the literature.[141]

N-Boc-N-methylalanine

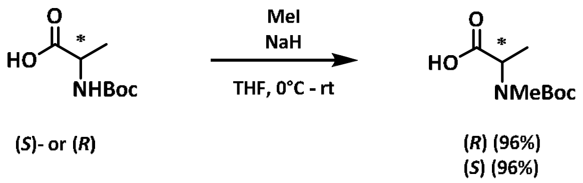

The title compound was prepared according to a modified literature procedure.[142]

(*L*)- or (*D*)-*N*-Boc-alanine (6 g, 31.7 mmol, 1 equiv.) was dissolved in anhydrous THF (100 mL) under argon and cooled to 0 °C. Sodium hydride (60% in mineral oil, 3.8 g, 95.2 mmol, 3 equiv.) was added in three portions. The reaction was stirred at 0 °C for one hour. Methyl iodide (16 mL, 254 mmol, 8 equiv.) was added via syringe. Then the reaction was allowed to warm to rt and stirring continued for 24 hours.

The reaction was quenched with 300 mL of water. The mixture was extracted with 100 mL Et_2O, the organic layer was separated and extracted with 30 mL of satd. $NaHCO_3$. The combined aqueous layers were acidified with 6M HCl to pH = 3 and extracted with 3 x 100 mL of EtOAc. The combined organic layers were washed with 2 x 50 mL of satd. $Na_2S_2O_3$, dried over anhydrous sodium sulfate and evaporated.

(*S*)-C18

**Yield**	6.19 g (96%) colorless oil
**Specific rotation**	α_D^{20} = – 41.81° (*c* 0.91, CH_2Cl_2)

(*R*)-C18

**Yield**	6.17 mg (96%) colorless oil
**Specific rotation**	α_D^{20} = + 48.24° (*c* 1.01, CH_2Cl_2)

**Molecular formula, m.w.**	$C_9H_{17}NO_4$, 203.24
**M.p.**	85 - 89 °C
**^{1}H-NMR (400 MHz, DMSO-*d6*)**	Mixture of rotamers. δ 1.24 – 1.33 (m, 3H), 1.32 – 1.45 (m, 9H), 2.73 (s, 3H), 4.17 – 4.39 (m, 1H), 4.44 – 4.68 (m, 1H)
**^{13}C-NMR (101 MHz, DMSO-*d6*)**	Mixture of rotamers. δ 14.6 (q), 15.2 (q), 27.9 (q), 28.0 (q), 30.6 (q), 31.5 (q), 53.2 (d), 54.8 (d), 79.0 (s), 154.6 (s), 155.0 (s), 173.3 (s), 173.4 (s)

tert-Butyl (1-(methoxy(methyl)amino)-1-oxopropan-2-yl)(methyl)carbamate

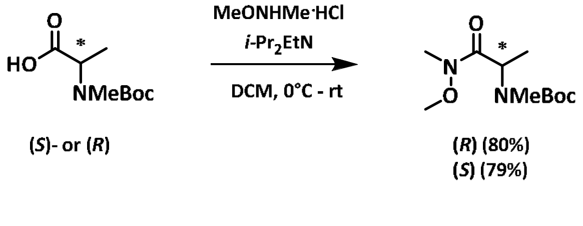

The title compound was prepared according to a modified literature procedure.[143]

(R)- or (S)-N-Boc-*N*-methylalanine (5 g, 24.6 mmol, 1 equiv.) was dissolved in anhydrous DCM (100 mL) under argon. 1-Hydroxybenzotriazole (4.3 g, 32.0 mmol, 1.3 equiv.), *N,O*-dimethylhydroxylamine hydrochloride (3.12 g, 32.0 mmol, 1.3 equiv.) and *i*-Pr_2EtN (9.5 g, 73.8 mmol, 3 equiv.) were added successively. The reaction mixture was cooled to 0 °C and EDCI hydrochloride (6.1 g, 32.0 mmol, 1.3 equiv.) was added. The reaction was allowed to warm to rt and was stirred overnight.

The reaction was quenched by adding 100 mL of water and extracted with 2 x 100 mL of DCM. The combined organic layers were washed with 0.5 M HCl (50 mL), satd. $NaHCO_3$ (50 mL) and brine (50 mL), dried over anhydrous magnesium sulfate and evaporated. The crude product was purified by flash column chromatography (90 g SiO_2, LP/EtOAc 12%).

(*S*)

**Yield**	4.78 g (79%) colorless oil
**Specific rotation**	α_D^{20} = – 55.49° (*c* 1.06, CH_2Cl_2)

(*R*)

**Yield**	4.84 g (80%) colorless oil
**Specific rotation**	α_D^{20} = + 56.34° (*c* 1.10, CH_2Cl_2)

**Molecular formula, m.w.**	$C_{11}H_{22}N_2O_4$, 246.31
**^{1}H-NMR (400 MHz, DMSO-*d*$_6$)**	Mixture of rotamers. δ 1.16 – 1.25 (m, 3H), 1.35 – 1.42 (m, 9H), 2.74 (s, 3H), 3.09 (s, 3H), 3.67 (s, 3H), 4.77 (d, *J* = 6.3 Hz, 1H), 4.87 – 5.15 (m, 1H)
**^{13}C-NMR (101 MHz, DMSO-*d*$_6$)**	Mixture of rotamers. δ = 14.4 (q), 14.7 (q), 28.0 (q), 29.7 (q), 49.7 (q), 51.2 (d), 61.1 (q), 79.0 (s), 154.4 (s), 154.8 (s), 171.9 (s)
**HR-ESI-MS**	m/z 247.1672 $[M+H]^+$ (calcd 247.1659, diff –1.91ppm)

General Procedure A: Synthesis of Grignard reagents

A three-necked flask equipped with condenser, septum and magnetic stirring bar was charged with magnesium turnings (243 mL, 10 mmol, 1 equiv.). The apparatus was evacuated, dried using a hot-air gun and set under argon. 1 - 2 mL of solvent were added, followed by dropwise addition of undiluted arylbromide. Once the reaction had started as indicated by a color change and the formation of an exotherm, the remaining solvent was added (typically 10 mL of solvent was used in total to give an approx. 1M solution of Grignard reagent). The remaining aryl bromide was slowly added and the reaction was stirred for the time indicated until the magnesium was fully, or almost fully, dissolved.

4-Tolylmagnesium bromide

**Concentration, solvent**	1M in THF
**Reaction time**	Drop-wise addition of ArBr over 15 min, 1h heated to 50 °C
**Appearance**	Brown solution

General Procedure B: Addition of Grignard reagents to alanine-derived Weinreb amides

In a Schlenk-tube equipped with septum and magnetic stirring bar, *(S)- or (R)-tert*-butyl (1-(methoxy(methyl)amino)-1-oxopropan-2-yl)(methyl)carbamate (253 mg, 1 mmol, 1 equiv.) was

dissolved in anhydrous THF (7 mL) and cooled to 0 °C. A solution of arylmagnesium bromide (1 M, 3 mL, 3 mmol, 3 euqiv.) was added dropwise. In some cases Grignard solutions of different concentrations were used. In these cases, the amount of solvent used to dissolve the starting material was adjusted to give a final concentration of 1 mmol/10 mL. The reaction was maintained at 0 °C for one hour, warmed to rt and stirred for 0.5 - 2 hours until full conversion was detected by TLC.

The reaction was cooled to 0 °C and quenched by addition of satd. NH_4Cl (5 mL) and water (10 mL). The mixture was extracted with 3 x 10 mL of Et_2O, dried over anhydrous magnesium sulfate and evaporated. The pure product was obtained after flash column chromatography on silica using LP/EtOAc mixtures.

General Procedure C: Boc-deprotection of mephedrone derivatives

In a 8-mL screw-cap vial Boc-protected mephedrone derivatives (1 equiv.) were treated with either 6 M HCl_{aq} or 6 M HCl in H_2O/dioxane 4:1 (this solution was prepared by mixing 50 mL of HCl conc. and 20 mL of dioxane and diluting the mixture to 100 mL with deionized water) to give a 0.2 M solution at 0 °C and stirred for 48 hours. After completion of the reaction a slightly opaque solution was obtained. The reaction mixture was extracted with 2 x 2 mL of Et_2O and the aqueous layer was evaporated in a stream of pressurized air. The residue was taken up in deionized water, filtered and lyophilized.

tert-Butyl methyl(1-oxo-1-(p-tolyl)propan-2-yl)carbamate

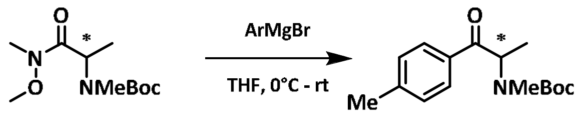

(*S*)

**General procedure**	B
**Reaction scale**	253 mg (1 mmol)
**Grignard reagent**	4-Tolylmagnesium bromide, 1M in THF
**Reaction time**	1 h at 0 °C, 0.5 h at rt
**Purification**	MPLC, 50 g SiO_2, LP/EtOAc 9:1
**Yield**	227 mg (82%) colorless oil
**Specific rotation**	α_D^{20} = – 142.0° (*c* 0.67, CH_2Cl_2)
**ee (HPLC)**	99%

(*R*)

**General procedure**	B
**Reaction scale**	253 mg (1 mmol)
**Grignard reagent**	4-Tolylmagnesium bromide, 1M in THF
**Reaction time**	1 h at 0 °C, 0.5 h at rt
**Purification**	MPLC, 50 g SiO_2, LP/EtOAc 9:1
**Yield**	241 mg (87%) colorless oil

**Specific rotation**	α_D^{20} = + 151.4° (*c* 0.79, CH_2Cl_2)
**ee (HPLC)**	99%

**Molecular formula, m.w.**	$C_{16}H_{23}NO_3$, 277.36
**^{1}H-NMR (400 MHz, DMSO-*d*$_6$)**	Mixture of rotamers. δ 1.18 – 1.43 (m, 12H), 2.36 (s, 3H), 2.62 (s, 1H), 2.79 (s, 1H), 3.34 (s, 1H), 5.08 (q, *J* = 6.6 Hz, 1H), 5.42 (q, *J* = 6.6 Hz, 1H), 7.22 – 7.37 (m, 2H), 7.70 – 7.84 (m, 2H)
**^{13}C-NMR (101 MHz, DMSO-*d*$_6$)**	Mixture of rotamers. δ 13.3 (q), 13.8 (q), 21.1 (q), 27.7 (q), 27.9 (q), 30.2 (q), 31.7 (q), 55.1 (d), 57.3 (d), 79.3 (s), 79.4 (s), 128.0 (d), 129.1 (d), 132.8 (s), 133.1 (s), 143.2 (s), 143.4 (s), 153.8 (s), 154.6 (s), 198.6 (s), 198.7 (s)
**HR-ESI-MS**	m/z 278.1758 $[M+H]^+$ (calcd 278.1757, diff 0.06 ppm)

tert-*Butyl ((*syn*)-1-hydroxy-1-(*p*-tolyl)propan-2-yl)carbamate*

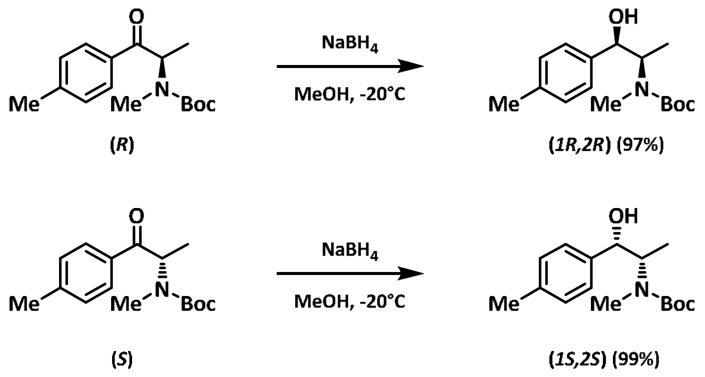

The title compounds were prepared according to a modified literature procedure.[153]

In a round-bottom flask equipped with septum an argon balloon, *tert*-butyl (*S*)-methyl-(1-oxo-1-(*p*-tolyl)propan-2-yl)carbamate (374 mg, 1.35 mmol, 1 equiv.) was dissolved in anhydrous methanol (18 mL) and cooled to - 20 °C. Then sodium borohydride (105 mg, 2.87 mmol, 2 equiv.) was added and the reaction was stirred for 2 hours at that temperature when TLC analysis showed full consumption of the starting material.

The reaction was quenched by addition of water (4.6 mL) and concentrated *in vacuo* and lyophilized. The solid residue was taken up in 2 mL of water and extracted with EtOAc (35 mL). The organic layer was washed with brine, dried over anhydrous magnesium sulfate and evaporated. The products were obtained as single syn diastereomers.

(1*S*, 2*S*)

**Yield**	372 mg (99%) colorless solid
**Specific rotation**	α_D^{20} = + 86.4° (*c* 0.67, CH_2Cl_2)
**ee (HPLC)**	98%

(1*R*, 2*R*)

The title compound was prepared using the analogous procedure from *tert*-butyl (*R*)-methyl-(1-oxo-1-(*p*-tolyl)propan-2-yl)carbamate

**Yield**	387 mg (97%) colorless solid
**Specific rotation**	α_D^{20} = – 84.9° (*c* 0.69, CH_2Cl_2)
**ee (HPLC)**	98%

**Molecular formula, m.w.**	$C_{16}H_{25}NO_3$, 279.38
**M.p.**	78 - 79 °C
**^{1}H-NMR (400 MHz, $CDCl_3$)**	δ 1.00 (s, 3H), 1.47 (s, 9H), 2.34 (s, 3H), 2.65 – 2.93 (m, 3H), 4.17 (p, *J* = 6.5 Hz, 1H), 4.43 – 4.58 (m, 1H), 7.15 (d, *J* = 7.8 Hz, 2H), 7.23 (d, *J* = 7.9 Hz, 2H)
**^{13}C-NMR (400 MHz, $CDCl_3$)**	δ 14.8 (q), 21.3 (q), 28.6 (q), 30.9 (q), 58.3 (d), 77.4 (d), 80.2 (s), 126.8 (d), 129.2 (d), 137.5 (s), 139.4 (s), 157.7 (s)
**HR-ESI-MS**	m/z 280.1920 $[M+H]^+$ (calcd 280.1940, diff – 4.43 ppm)

(syn)-2-(Methylamino)-1-(p-tolyl)propan-1-ol hydrochloride

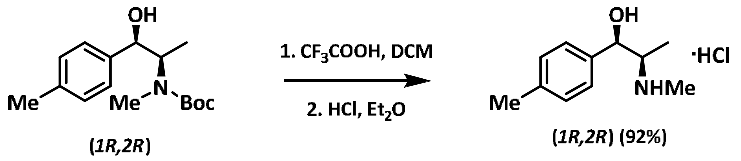

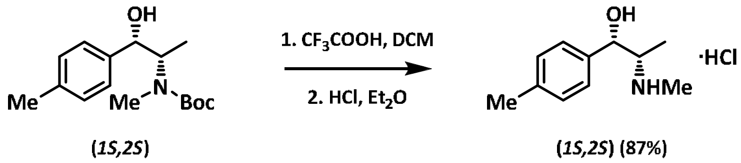

In a round-bottom flask with septum *tert*-butyl (*1S,2S*)-((*syn*)-1-hydroxy-1-(*p*-tolyl)propan-2-yl)carbamate (316 mg, 1.13 mmol, 1 equiv.) was dissolved in dichloromethane (11.5 mL) and cooled to 0 °C. Trifluoromethanesulfonic acid (2.09 g, 18.3 mmol, 15 equiv.) was added and the reaction was stirred at that temperature for 2.5 hours and monitored with TLC.

The solvent was evaporated in a stream of pressurized air. The oily residue was taken up in 14 mL of NaOH solution (1M, saturated with NaCl). The aqueous layer was extracted with 4 x 15 mL of Et_2O. The combined organic layers were dried over anhydrous magnesium sulfate. Then 2M HCl/Et_2O (1 mL) was added and the product precipitated as a colorless solid. The solvent was evaporated and the residue was washed with Et_2O (7 mL). The pure product was obtained after drying of the residue *in vacuo*.

(1*S*,2*S*)

**Yield**	213 mg (87% overall) colorless solid
**Specific rotation**	α_D^{20} = + 52.8° (*c* 0.90, H_2O)
**ee (HPLC)**	>99%

(1*R*,2*R*)

The title compound was prepared using the analogous procedure from *tert*-butyl (1*R*,2*R*)-((*syn*)-1-hydroxy-1-(*p*-tolyl)propan-2-yl)carbamate

**Yield**	239 mg (92% overall) colorless solid
**Specific rotation**	α_D^{20} = – 56.5° (*c* 1.07, H_2O)
**ee (HPLC)**	>99%

**Molecular formula, m.w.**	$C_{11}H_{18}ClNO$, 215.72
**M.p.**	194 - 195 °C
**^{1}H-NMR (400 MHz, D_2O)**	δ 1.10 (d, *J* = 6.7 Hz, 3H), 2.35 (s, 3H), 2.77 (s, 3H), 3.52 (p, *J* = 6.6 Hz, 1H), 4.64 (d, *J* = 9.2 Hz, 1H), 7.21 – 7.42 (m, 4H)
**^{13}C NMR (101 MHz, D_2O)**	δ 11.6 (q), 20.2 (q), 29.7 (q), 59.8 (d), 73.9 (d), 127.0 (d), 129.6 (d), 136.2 (s), 139.4 (s)
**HR-ESI-MS**	m/z 180.1393 $[M+H]^+$ (calcd 180.1390, diff – 5.68 ppm)

tert-*Butyl (1-(methoxy(methyl)amino)-1-oxopropan-2-yl)carbamate*

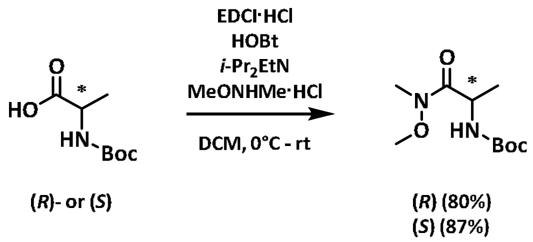

The title compound was prepared according to a modified literature procedure.[143]

N-Boc-alanine (3.0 g, 15.9 mmol, 1 equiv.), hydroxybenzotriazole (2.79 g, 20.6 mmol, 1.3 equiv.) and *N,O*-dimethylhydroxylamine hydrochloride (2.0 g, 20.6 mmol, 1.3 equiv.) were suspended in anhydrous dichloromethane (64 mL) and cooled to 0 °C. Then *i*-Pr_2EtN (6.15 g, 47.6 mmol, 3 equiv.) and EDCI hydrochloride (3.95 g, 20.6 mmol, 1.3 equiv.) were added successively, the reaction was maintained at 0 °C for one hour, allowed to warm to rt and stirred for 24h.

The reaction mixture was diluted with 300 mL of dichloromethane and washed with 2 x 150 mL 2M HCl, 2 x 150 mL satd. $NaHCO_3$, and 2 x 150 mL of brine. The organic layer was dried over anhydrous magnesium sulfate and evaporated.

(*S*)

**Yield**	3.41 g (87%) colorless oil
**Specific rotation**	α_D^{20} = + 1.39° (*c* 0.94, CH_2Cl_2)

(*R*)

**Yield**	3.14 mg (80%) colorless oil
**Specific rotation**	α_D^{20} = – 1.32° (*c* 1.01, CH_2Cl_2), lit.[151]: α_D^{28} = - 3.3° (*c* 0.5, CH_2Cl_2)

**Molecular formula, m.w.**	$C_{10}H_{20}N_2O_4$, 232.28

M.p. 147 - 150 °C (lit.[151]: 144 - 147 °C)
^{1}H-NMR (200 MHz, $CDCl_3$) δ 1.30 (d, *J* = 6.9 Hz, 3H), 1.42 (s, 9H), 3.19 (s, 3H), 3.75 (s, 3H), 4.58 – 4.75 (m, 1H), 5.04 – 5.46 (m, 1H)
^{13}C-NMR (50 MHz, $CDCl_3$) δ 18.7 (q), 28.4 (q), 32.2 (d), 46.5 (q), 61.6 (q), 79.5 (s), 155.2 (s), 173.6 (s)
HR-ESI-MS m/z 233.1502 $[M+H]^+$ (calcd 233.1502, diff –2.33ppm)
Spectral data were in agreement with those reported in the literature.[152]

tert-*Butyl (1-oxo-1-(*p*-tolyl)propan-2-yl)carbamate*

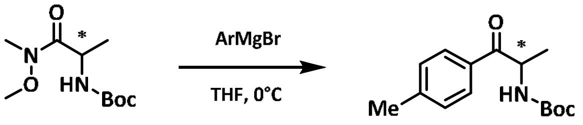

(*R*)- or (*S*) **(*R*) (70%)** **(*S*) (68%)**

tert-Butyl (1-(methoxy(methyl)amino)-1-oxopropan-2-yl)carbamate (2.5 g, 10.8 mmol, 1 equiv.) was dissolved in anhydrous THF (65 mL) under argon and cooled to 0 °C. Then a freshly prepared solution of 4-tolylmagnesium bromide in THF (1 M, 43 mL, 43 mmol, 4 equiv.) was added dropwise. The reaction was stirred at 0 °C for 1.5 hours when TLC analysis indicated full consumption of the starting material.

The reaction was quenched by addition of water (100 mL) and satd. NH_4Cl (100 mL), extracted with 3 x 100 mL Et_2O, dried over anhydrous magnesium sulfate and evaporated. The crude product was purified by flash column chromatography (90 g SiO_2, LP/EtOAc 6%) followed by recrystallization from ligroin.

(*S*)
Yield 1.50 g (68%) colorless solid
Specific rotation α_D^{20} = + 8.47° (*c* 1.37, CH_2Cl_2)
ee (HPLC) >99%

(*R*)
Yield 1.55 g (70%) colorless solid
Specific rotation α_D^{20} = – 8.81° (*c* 1.21, CH_2Cl_2)
ee (HPLC) >99%

Molecular formula, m.w. $C_{15}H_{21}NO_3$, 263.34
M.p. 101 - 102 °C
^{1}H-NMR (400 MHz, $CDCl_3$) δ 1.32 (d, *J* = 7.1 Hz, 3H), 1.39 (s, 9H), 2.35 (s, 3H), 5.20 (p, *J* = 7.1 Hz, 1H), 5.51 (d, *J* = 6.6 Hz, 1H), 7.22 (d, *J* = 8.0 Hz, 2H), 7.80 (d, *J* = 7.9 Hz, 2H)
^{13}C-NMR (101 MHz, $CDCl_3$) δ 20.3 (q), 21.8 (q), 28.5 (q), 51.1 (d), 79.8 (s), 128.9 (d), 129.7 (d), 131.8 (s), 144.8 (s), 155.3 (s), 199.1 (s)
HR-ESI-MS m/z 264.1594 $[M+H]^+$ (calcd 264.1601, diff –0.18ppm)

N-(Ethoxycarbonyl)alanine

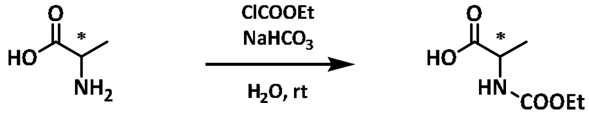

(*R*)- or (*S*)-alanine **(*R*) (90%)** **(*S*) (94%)**

The title compound was prepared according to a modified literature procedure.[154]

D- or L-alanine (3.0 g, 33.7 mmol, 1 equiv.) was dissolved in satd. $NaHCO_3$ (135 mL). Ethyl chloroformate (4.8 mL, 50.5 mmol, 1.5 equiv.) was added and the reaction mixture was stirred at rt for 24 h.

The reaction mixture was washed with 2 x 30 mL of Et_2O, cooled to 0 °C and acidified with 1M HCl to pH = 1. The aqueous layer was extracted with 3 x 50 mL of EtOAc. The combined organic layers were dried over anhydrous magnesium sulfate and evaporated to give the product as viscous oil.

(*S*)

**Yield**	5.08 g (94%) colorless oil
**Specific rotation**	α_D^{20} = – 14.81° (*c* 1.12, CH_2Cl_2), lit.[155]: α_D^{22} = – 15.3° (*c* 2.0, CH_2Cl_2)

(*R*)

**Yield**	4.89 g (90%) colorless oil
**Specific rotation**	α_D^{20} = + 15.92° (*c* 0.94, CH_2Cl_2)

**Molecular formula, m.w.**	$C_6H_{11}NO_4$, 161.16
**^{1}H-NMR (400 MHz, DMSO-*d*$_6$)**	δ 1.15 (t, *J* = 7.2 Hz, 3H), 1.24 (d, *J* = 7.4 Hz, 3H), 3.93 – 4.01 (m, 3H), 7.39 (d, *J* = 7.6 Hz, 1H), 12.47 (s, 1H)
**^{13}C-NMR (101 MHz, DMSO-*d*$_6$)**	δ 14.6 (q), 17.1 (q), 49.1 (d), 59.7 (t), 155.9 (s), 174.5 (s)

Ethyl (1-(methoxy(methyl)amino)-1-oxopropan-2-yl)carbamate

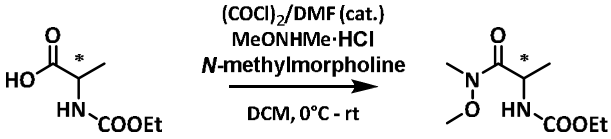

(*R*)- or (*S*) **(*R*) (55%)** **(*S*) (59%)**

The title compound was prepared according to a modified literature procedure.[156]

(*R*)- or (*S*)-*N*-(ethoxycarbonyl)alanine (4.0 g, 24.8 mmol, 1 equiv.) was dissolved in anhydrous dichloromethane (50 mL) in a round-bottom flask equipped with septum and argon balloon. Oxalyl chloride (2.11 mL, 24.8 mmol, 1 equiv.) was added via syringe, followed by 3 drops of DMF. The reaction mixture was stirred overnight at rt. Then *N,O*-dimethylhydroxylamine hydrochloride (2.89 g,

29.8 mmol, 1.2 equiv.) was added. After cooling the reaction to 0 °C, *N*-methylmorpholine (8.2 mL, 74.5 mmol, 3 equiv.) was added dropwise. Stirring was continued for 30 min at rt.

The reaction mixture was diluted with Et_2O and successively washed with 1M HCl, satd. $NaHCO_3$ and brine. Evaporation of the solvent gave the crude product, which was purified using flash column chromatography (90 g SiO_2, LP/EtOAc 2:1).

(*S*)

**Yield**	2.98 g (59%) colorless solid
**Specific rotation**	α_D^{20} = + 0.42° (*c* 1.07, CH_2Cl_2), lit.: not reported

(*R*)

**Yield**	2.81 g (55%) colorless solid
**Specific rotation**	α_D^{20} = – 0.46° (*c* 1.02, CH_2Cl_2), lit.: not reported

**Molecular formula, m.w.**	$C_8H_{16}N_2O_4$, 204.23
**M.p.**	46 - 48 °C (lit.: not reported)
**^{1}H-NMR (400 MHz, $CDCl_3$)**	δ 1.22 (t, *J* = 7.1 Hz, 3H), 1.31 (d, *J* = 6.9 Hz, 3H), 3.19 (s, 3H), 3.75 (s, 3H), 4.08 (q, *J* = 7.1 Hz, 2H), 4.66 – 4.74 (m, 1H), 5.42 (d, *J* = 6.5 Hz, 1H)
**^{13}C-NMR (101 MHz, $CDCl_3$)**	δ 14.7 (q), 18.7 (q), 32.3 (d), 47.0 (q), 61.0 (t), 61.7 (q), 156.1 (s), 173.5 (s)
**HR-ESI-MS**	m/z 205.1191 $[M+H]^+$ (calcd 205.1189, diff –4.06ppm)

Spectral data were in agreement with those reported in the literature.[156]

Ethyl (1-oxo-1-(p-tolyl)propan-2-yl)carbamate

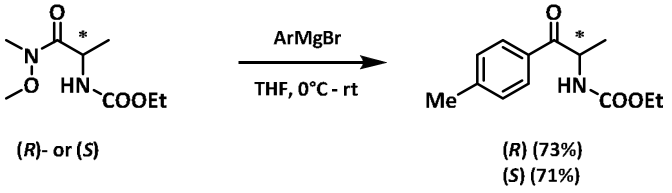

(S)- or (R)-Ethyl (1-(methoxy(methyl)amino)-1-oxopropan-2-yl)carbamate (1.5 g, 7.35 mmol, 1 equiv.) was dissolved in anhydrous THF (44 mL) under argon and cooled to 0 °C. Then a freshly prepared solution of
4-tolylmagnesium bromide (1M in THF, 29.4 mL, 29.4 mmol, 4 equiv.) was added dropwise. The reaction was maintained at 0 °C for 3 hours when TLC showed full consumption of starting material.

The reaction was quenched by sequential addition of satd. NH_4Cl (30 mL) and water (30 mL). The biphasic mixture was extracted with 3 x 60 mL of Et_2O. The combined organic layers were dried over anhydrous magnesium sulfate and evaporated. The crude product was purified by flash column chromatography (90 g SiO_2, LP/EtOAc 2:1).

(*S*)

Yield 1.23 g (71 %) colorless solid
Specific rotation α_D^{20} = + 18.42° (*c* 1.05, CH_2Cl_2)
ee (HPLC) >99 %

(*R*)
Yield 1.26 g (73 %) colorless solid
Specific rotation α_D^{20} = – 19.95° (*c* 0.94, CH_2Cl_2)
ee (HPLC) >99%

Molecular formula, m.w. $C_{13}H_{17}NO_3$, 235.28
M.p. 60 - 63 °C
^{1}H-NMR (200 MHz, $CDCl_3$) δ 1.26 (t, *J* = 7.1 Hz, 3H), 1.41 (d, *J* = 7.1 Hz, 3H), 2.42 (s, 3H), 4.13 (q, *J* = 7.1 Hz, 2H), 5.30 (p, *J* = 7.0 Hz, 1H), 5.75 (d, *J* = 7.1 Hz, 1H), 7.29 (d, *J* = 8.0 Hz, 2H), 7.88 (d, *J* = 8.3 Hz, 2H)
^{13}C NMR (101 MHz, $CDCl_3$) δ 14.7 (q), 20.3 (q), 21.9 (q), 51.5 (d), 61.1 (t), 129.0 (d), 129.7 (d), 131.6 (s), 145.0 (s), 156.1 (s), 198.8 (s)
HR-ESI-MS m/z 236.1292 $[M+H]^+$ (calcd 236.1288, diff –0.98ppm)

tert-*Butyl ((*anti*)-1-hydroxy-1-(*p*-tolyl)propan-2-yl)carbamate*

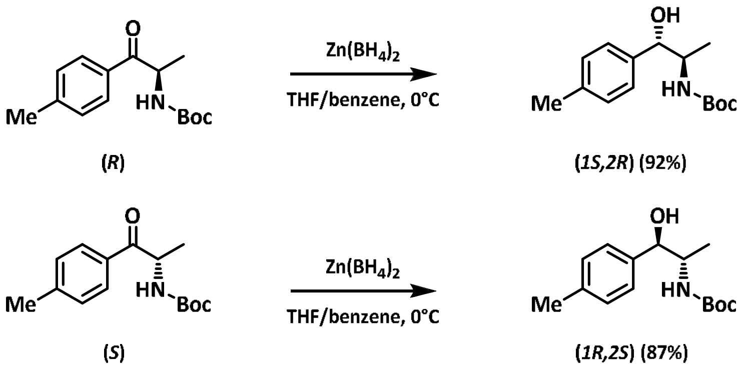

The title compound was prepared according to a modified literature procedure.[100]

Tert-butyl (1-oxo-1-(*p*-tolyl)propan-2-yl)carbamate (400 mg, 1.52 mmol, 1 equiv.) was dissolved in 40 mL of benzene and cooled to 0 °C. Once the solution was partially solidified, $Zn(BH_4)_2$ (0.145 M in THF, 10.5 mL, 1.52 mmol, 1 equiv.) was added dropwise. Full conversion as indicated by TLC was reached after 2 hours.

The reaction was quenched by addition of 100 mL of ice water and extracted with 3 x 100 mL of EtOAc. The organic layer was washed with 50 mL of brine, dried over anhydrous magnesium sulfate and evaporated. After flash column chromatography (20 g SiO_2, LP/EtOAc 4:1) fractions containing the pure anti product and fractions containing a mixture of diastereomers were obtained.

(1*R*, 2*S*)
Yield 365 mg (91%, 13.2:1 dr) mixture of diastereomers;
220 mg (55 %) pure anti-diastereomer after column chromatography, colorless solid
Specific rotation α_D^{20} = – 45.43° (*c* 1.13, CH_2Cl_2)

(1*S*, 2*R*)

**Yield**	395 mg (98%, 9.3:1 dr) mixture of diastereomers; 157 mg (39 %) pure anti-diastereomer after column chromatography, colorless solid
**Specific rotation**	α_D^{20} = + 50.21° (*c* 0.99, CH_2Cl_2)
**Molecular formula, m.w.**	$C_{15}H_{23}NO_3$, 265.35
**M.p.**	129 - 130 °C
**^{1}H-NMR (400 MHz, $CDCl_3$)**	δ 0.97 (d, *J* = 6.9 Hz, 3H, CHCH_3), 1.46 (s, 9H), 2.34 (s, 3H), 3.25 (s, 1H), 3.91 – 4.04 (m, 1H), 4.63 – 4.73 (m, 1H), 4.80 (t, 1H), 7.15 (d, *J* = 7.9 Hz, 2H), 7.22 (d, *J* = 8.1 Hz, 2H)
**^{13}C-NMR (101 MHz, $CDCl_3$)**	δ = 14.9 (q), 21.2 (q), 28.5 (q), 52.1 (d), 76.8 (d), 79.8 (s), 126.4 (d), 128.9 (d), 137.1 (s), 138.0 (s), 156.5 (s)
**HR-ESI-MS**	m/z 266.1750 $[M+H]^+$ (calcd 266.1757, diff 0.44 ppm)

Ethyl ((anti)-1-hydroxy-1-(p-tolyl)propan-2-yl)carbamate

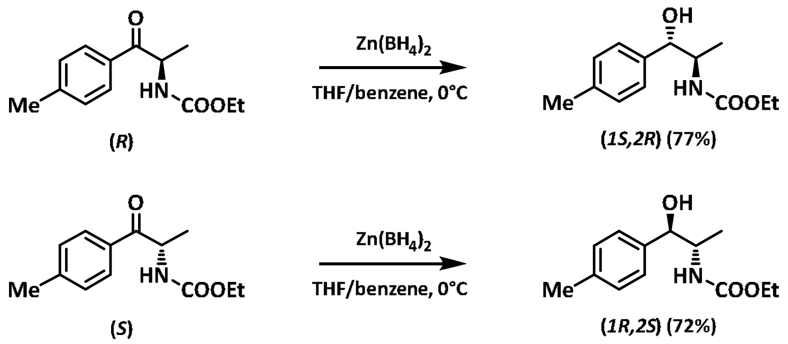

The title compounds were prepared according to a modified literature procedure.[100]

(1*R*,2*S*)

Ethyl (1-oxo-1-(*p*-tolyl)propan-2-yl)carbamate (396 mg, 1.68 mmol, 1 equiv.) was dissolved in 46 mL of benzene and cooled to 0 °C. Once the solution was partially solidified, $Zn(BH_4)_2$ (0.145 M in THF, 11.6 mL, 1.68 mmol, 1 equiv.) was added dropwise. Full conversion as indicated by TLC was reached after 1 hour.

The reaction was quenched by addition of 100 mL of ice water and extracted with 3 x 150 mL of EtOAc. The organic layer was dried over anhydrous magnesium sulfate and evaporated. The pure product was obtained after flash column chromatography (20 g SiO_2, LP/EtOAc 4:1) as a single diastereomer.

**Yield**	289 mg (72%) colorless solid
**Specific rotation**	α_D^{20} = – 60.39° (*c* 0.85, CH_2Cl_2)

(1*S*,2*R*)

**Reaction scale**	488 mg starting material (2.07 mmol, 1 equiv.)

**Yield**	381 g (77%) colorless solid
**Specific rotation**	α_D^{20} = + 57.74° (*c* 1.07, CH_2Cl_2)

**Molecular formula, m.w.**	$C_{13}H_{19}NO_3$, 237.30
**M.p.**	79 - 81 °C
**^{1}H-NMR (400 MHz, $CDCl_3$)**	1 : 1.3 mixture of rotamers. δ 1.36 (s, 3H), 1.38 (s, 9H), 3.99 – 4.19 (m, 1H), 4.16 – 4.43 (m, 1H), 5.04 – 5.10 (m, 1H), 6.76 (s, 1H), 11.42 (s, 1H)
**^{13}C-NMR (101 MHz, $CDCl_3$)**	δ 18.5 (q), 28.4 (q), 49.2 (d), 50.3 (d), 80.4 (s), 81.8 (s), 155.5 (s), 156.9 (s), 177.5 (s), 178.1 (s)
**HR-ESI-MS**	m/z 238.1447 $[M+H]^+$ (calcd 238.1444, diff –1.17ppm)

(anti)-2-(Methylamino)-1-(p-tolyl)propan-1-ol hydrochloride
(1*R*,2*S*) via reduction of the Boc-protected precursor

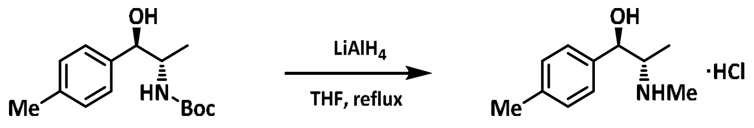

(1*R*,2*S*) (1*R*,2*S*) (50%)

The title compound was prepared according to a modified literature procedure.[157]

In a 8-mL screw-cap vial *tert*-butyl ((*1R,2S*)-1-hydroxy-1-(*p*-tolyl)propan-2-yl)carbamate (100 mg, 0.38 mmol, 1 equiv.) was dissolved in anhydrous THF (0.75 mL). Lithium aluminumhydride (43 mg, 1.13 mmol, 3 equiv.) was added and the reaction mixture was heated to 70 °C for 3 hours when full conversion was reached as indicated by TLC analysis.

The reaction was hydrolyzed by sequential addition of water (40 µl), 15% NaOH (40 µl) and water (120 µl) and stirred for 30 minutes. The resulting granular precipitate separated by filtration and extracted with 2 x 2 mL of EtOAc. The combined organic extracts were dried over anhydrous magnesium sulfate and evaporated. Flash column chromatography (20 g SiO_2, $CHCl_3$/MeOH 20%/NEt_3 1%) provided the pure free base as brown oil (46mg, 68%).

The free base was dissolved in 2.5 mL of anhydrous Et_2O and treated with an excess (0.25 mL) of 2M HCl/Et_2O. The precipitate was collected by centrifugation, washed with 2 x 2 mL of Et_2O and dried *in vacuo*.

**Free base**	
**Yield**	46 mg (68%) brown oil

**Hydrochloride**	
**Yield**	41 mg (50%) colorless solid
**Specific rotation**	α_D^{20} = – 44.66° (*c* 0.10, H_2O)
**ee (HPLC)**	99%

(1*S*,2*R*) via reduction of the ethoxycarbonyl-protected precursor

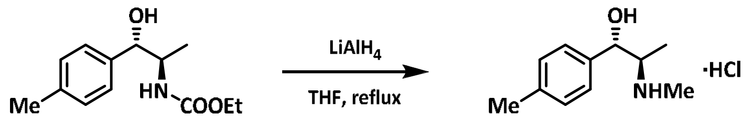

(1*S*,2*R*) (1*S*,2*R*) (64%)

The title compound was prepared according to a modified literature procedure.[157]

In a 8-mL screw-cap vial ethyl ((*1S,2R*)-1-hydroxy-1-(p-tolyl)propan-2-yl)carbamate (200 mg, 0.84 mmol, 1 equiv.) was dissolved in anhydrous THF (1.70 mL). Lithium aluminumhydride (96 mg, 2.52 mmol, 3 equiv.) was added and the reaction mixture was heated to 70 °C overnight when full conversion was reached as indicated by TLC analysis.

The reaction was hydrolyzed by addition of 15% NaOH (4 mL) and extracted with 5 x 2 mL of EtOAc. The combined organic extracts were dried over anhydrous magnesium sulfate and evaporated. Flash column chromatography (20 g SiO_2, $CHCl_3$/MeOH 20%/NEt_3 1%) provided the pure free base as colorless solid (107 mg, 71%).

The free base (90 mg, 0.5 mmol, 1 equiv.) was dissolved in 4.5 mL of anhydrous Et_2O and treated with an excess (0.45 mL) of 2M HCl/Et_2O. Collection of the precipitate by centrifugation, washing with 2 x 4 mL of Et_2O and drying *in vacuo* provided the pure hydrochloride as a colorless solid (91 mg, 90% from the free base).

**Free base**	
**Molecular formula, m.w.**	$C_{11}H_{17}NO$, 179.13
**^{1}H-NMR (400 MHz, $CDCl_3$)**	δ 0.87 (d, *J* = 6.5 Hz, 3H), 2.33 (s, 3H), 2.43 (s, 3H), 2.69 – 2.81 (m, 1H), 2.90 (s, 2H), 4.72 (d, *J* = 3.9 Hz, 1H), 7.13 (d, *J* = 8.0 Hz, 2H), 7.20 (d, *J* = 8.0 Hz, 2H)
**^{13}C-NMR (101 MHz, $CDCl_3$)**	δ = 14.0 (q), 21.2 (q), 33.9 (q), 60.6 (d), 73.3 (d), 126.2 (d), 128.9 (d), 136.7 (s), 138.8 (s)
**Hydrochloride**	
**Yield**	91 mg (64% overall) colorless solid
**Molecular formula, m.w.**	$C_{11}H_{18}ClNO$, 215.72
**M.p.**	sublimation > 200 °C
**^{1}H-NMR (400 MHz, D_2O)**	δ 1.15 (d, *J* = 6.8 Hz, 3H), 2.35 (s, 3H), 2.77 (s, 3H), 3.48 – 3.59 (m, 1H), 5.09 (d, *J* = 3.7 Hz, 1H), 7.32 (s, 4H)
**^{13}C-NMR (101 MHz, D_2O)**	δ = 9.8 (q), 20.2 (q), 30.7 (q), 60.0 (d), 71.4 (d), 126.1 (d), 129.3 (d), 135.4 (s), 138.7 (s)
**Specific rotation**	α_D^{20} = + 37.55° (*c* 0.21, H_2O)
**ee (HPLC)**	99%
**HR-ESI-MS**	m/z 180.1395 $[M+H]^+$ (calcd 180.1390, diff – 6.76 ppm)

Compound spectra

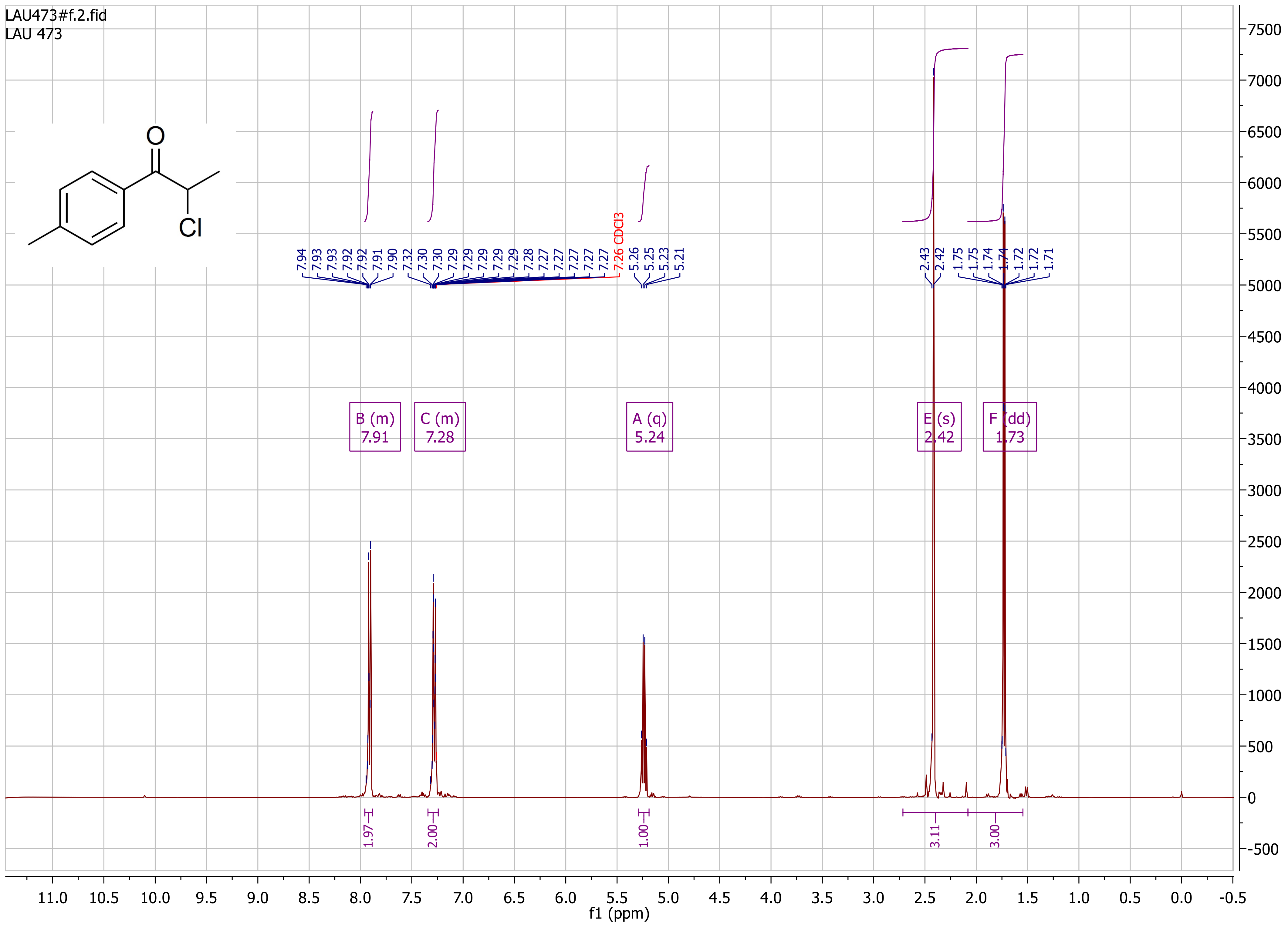

LAU473#f.2.fid
LAU 473
O
Cl
7.94
7.93
7.93
7.92
7.92
7.91
7.90
7.32
7.30
7.30
7.29
7.29
7.29
7.29
7.29
7.28
7.27
7.27
7.27
7.27
7.27
7.26 CDCl3
5.26
5.25
5.23
5.21
2.43
2.42
1.75
1.75
1.74
1.74
1.72
1.72
1.71
B (m)
7.91
C (m)
7.28
A (q)
5.24
E (s)
2.42
F (dd)
1.73
1.97
2.00
1.00
3.11
3.00
f1 (ppm)

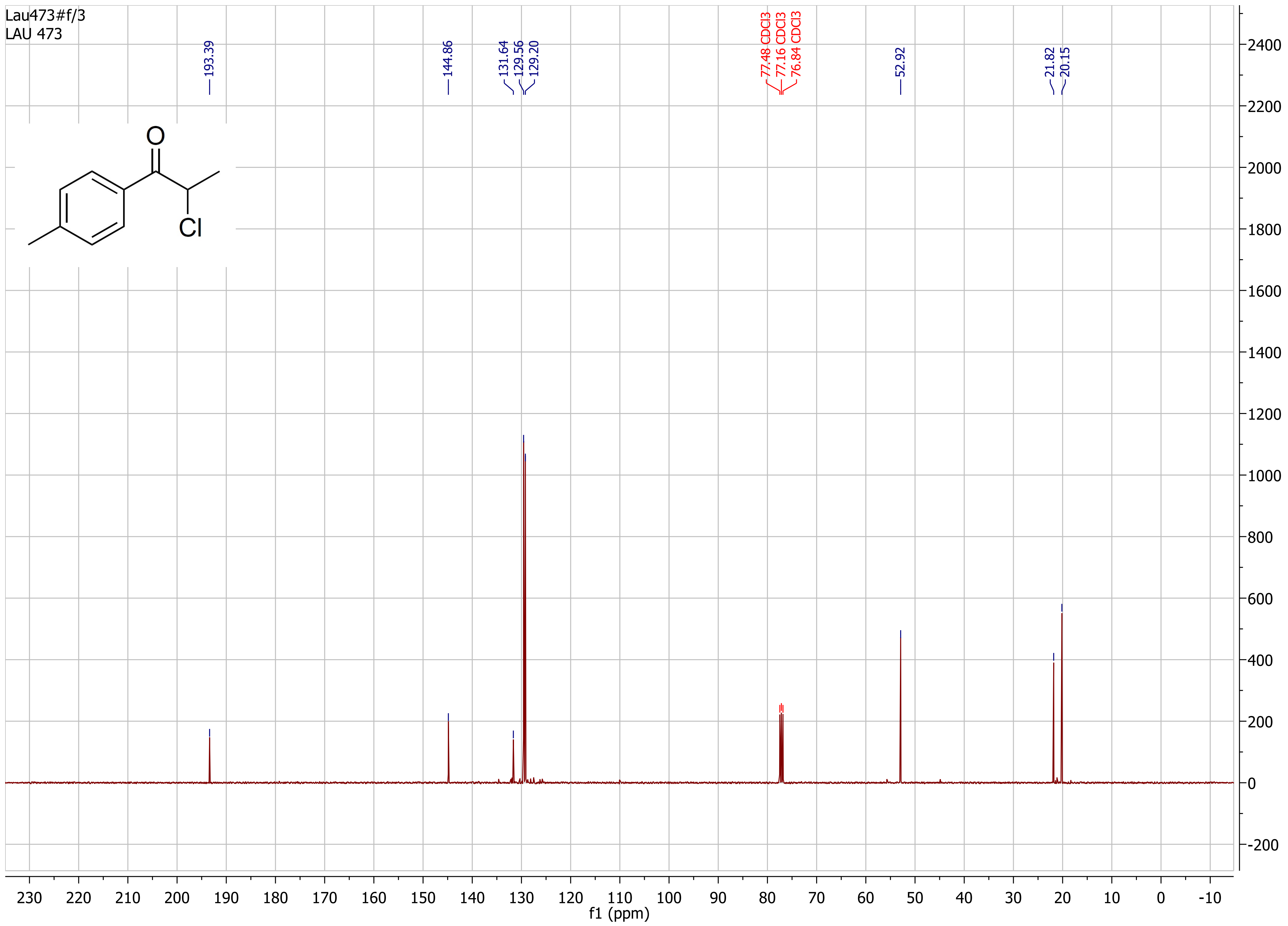

Lau473#f/3
LAU 473
193.39
144.86
131.64
129.56
129.20
77.48 CDCl3
77.16 CDCl3
76.84 CDCl3
52.92
21.82
20.15
O
Cl
f1 (ppm)

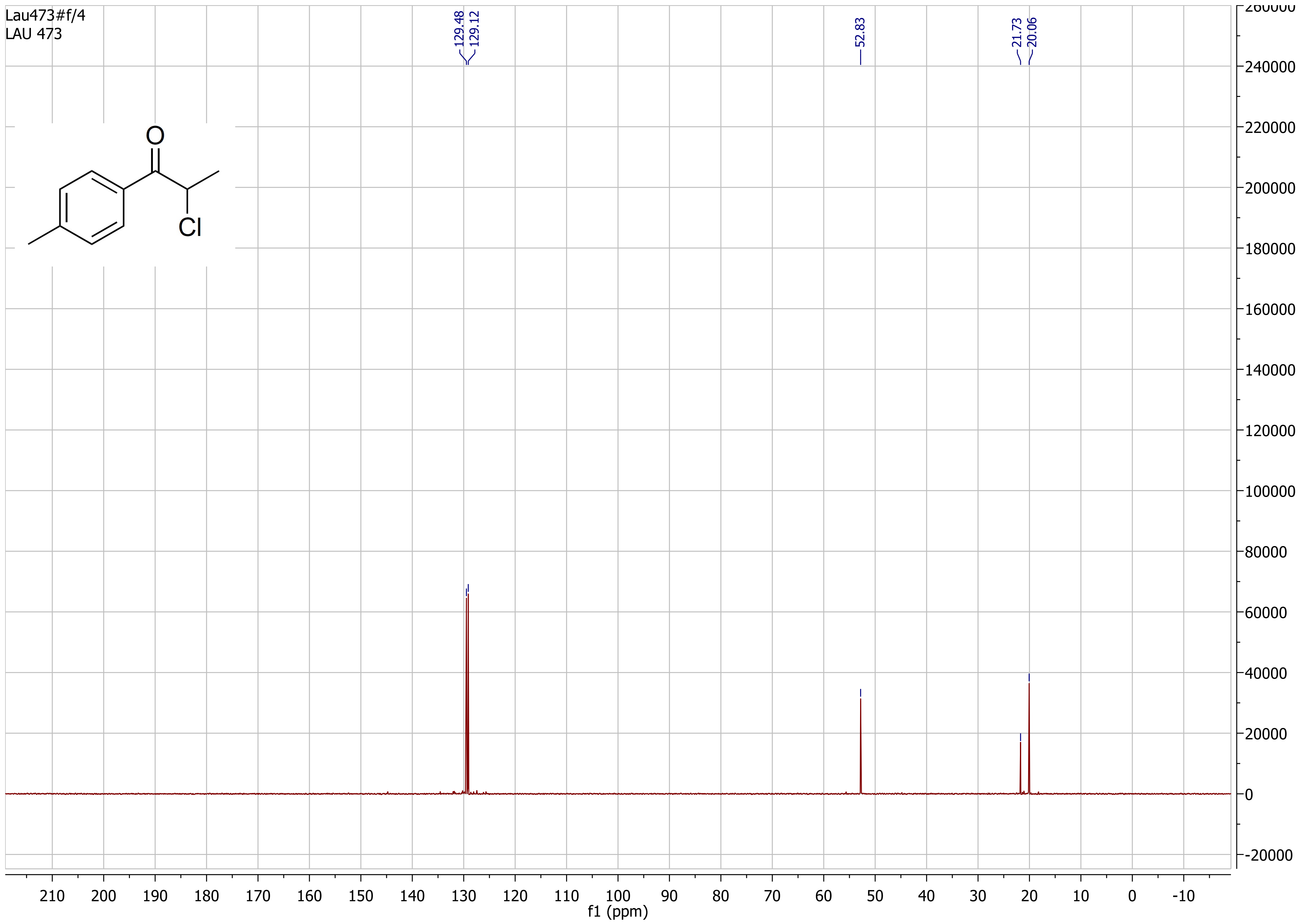

Lau473#f/4
LAU 473
O
Cl
129.48
129.12
52.83
21.73
20.06
260000
240000
220000
200000
180000
160000
140000
120000
100000
80000
60000
40000
20000
0
-20000
210
200
190
180
170
160
150
140
130
120
110
100
90
80
70
60
50
40
30
20
10
0
-10
f1 (ppm)

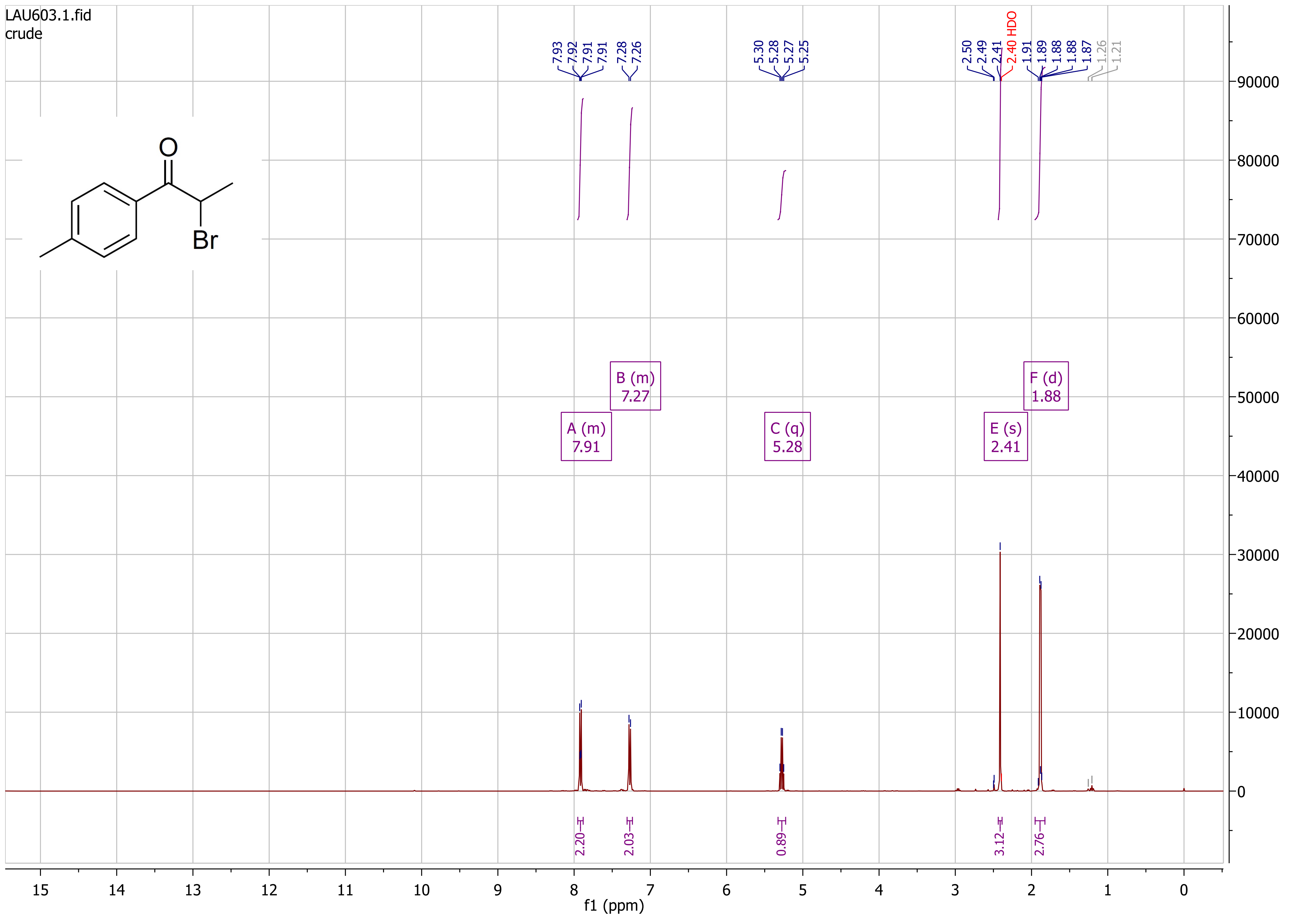

LAU603.1.fid
crude
Br
O
7.93
7.92
7.91
7.91
7.28
7.26
5.30
5.28
5.27
5.25
2.50
2.49
2.41
2.40 HDO
1.91
1.89
1.88
1.88
1.87
1.26
1.21
A (m) 7.91
B (m) 7.27
C (q) 5.28
E (s) 2.41
F (d) 1.88
2.20
2.03
0.89
3.12
2.76
f1 (ppm)

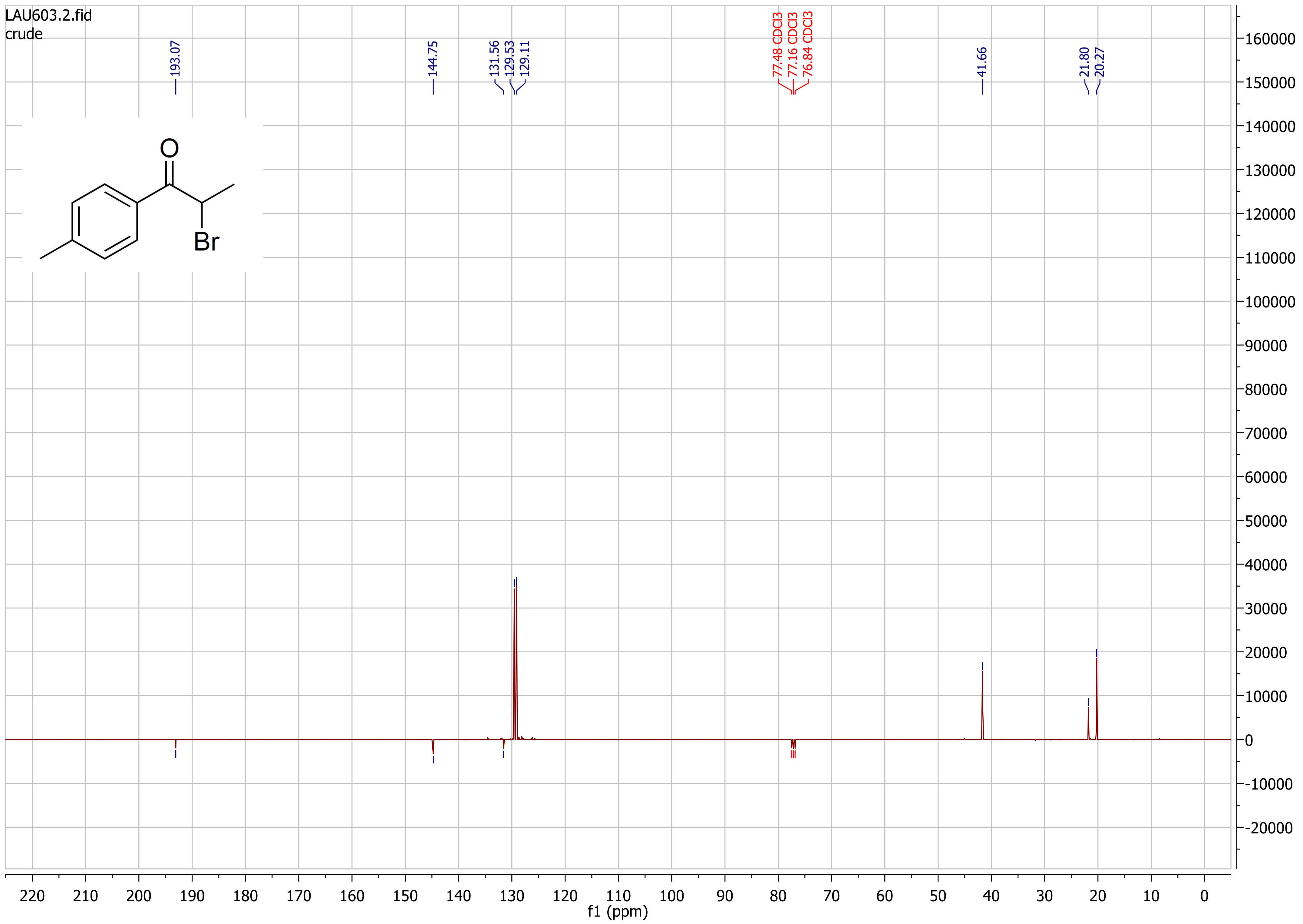
LAU603.2.fid
crude
193.07
144.75
131.56
129.53
129.11
77.48 CDCl3
77.16 CDCl3
76.84 CDCl3
41.66
21.80
20.27
O
Br
f1 (ppm)

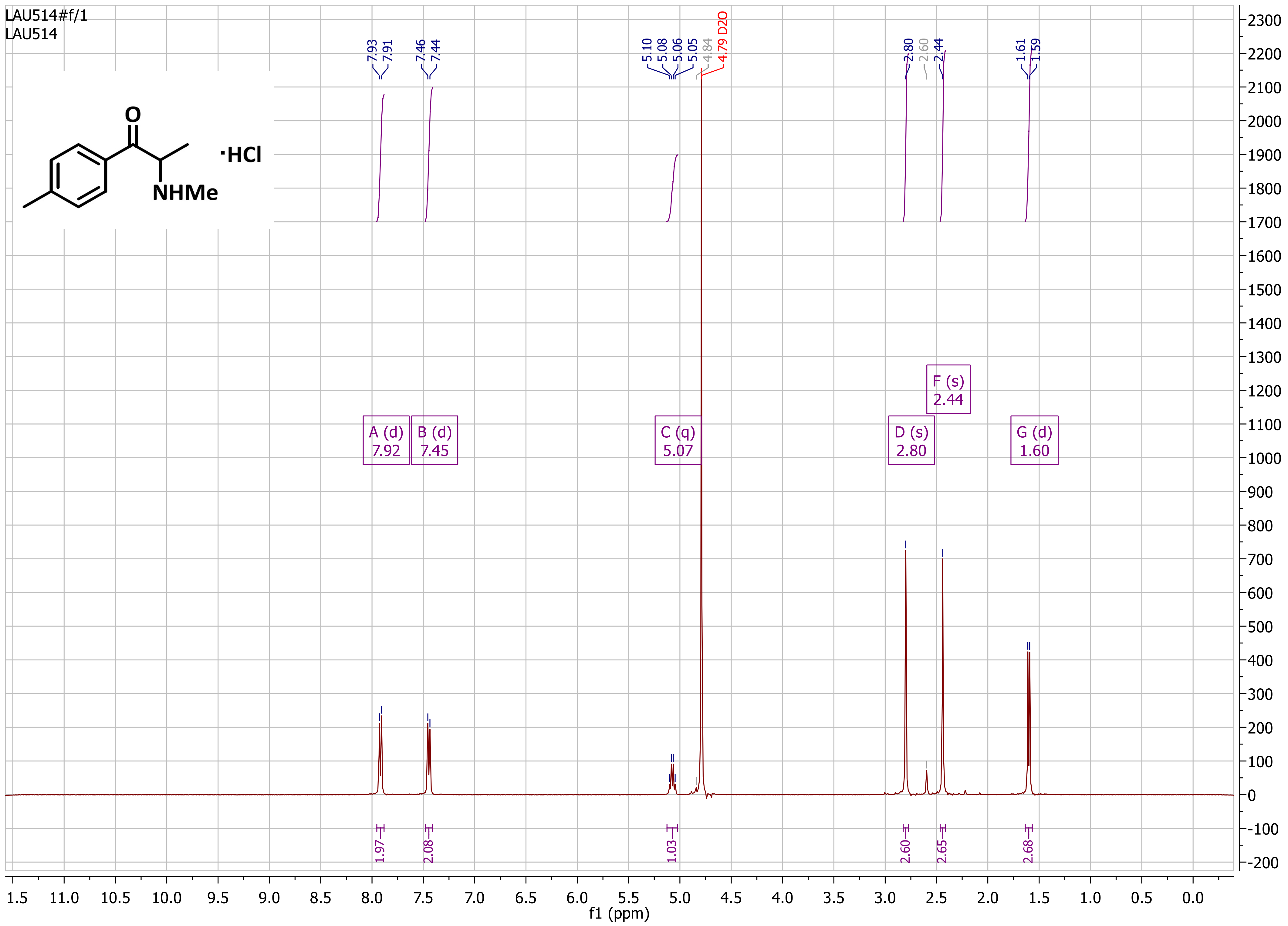
LAU514#f/1
LAU514
·HCl
NHMe
O
7.93
7.91
7.46
7.44
5.10
5.08
5.06
5.05
4.84
4.79 D2O
2.80
2.60
2.44
1.61
1.59
A (d) 7.92
B (d) 7.45
C (q) 5.07
D (s) 2.80
F (s) 2.44
G (d) 1.60
1.97
2.08
1.03
2.60
2.65
2.68
f1 (ppm)

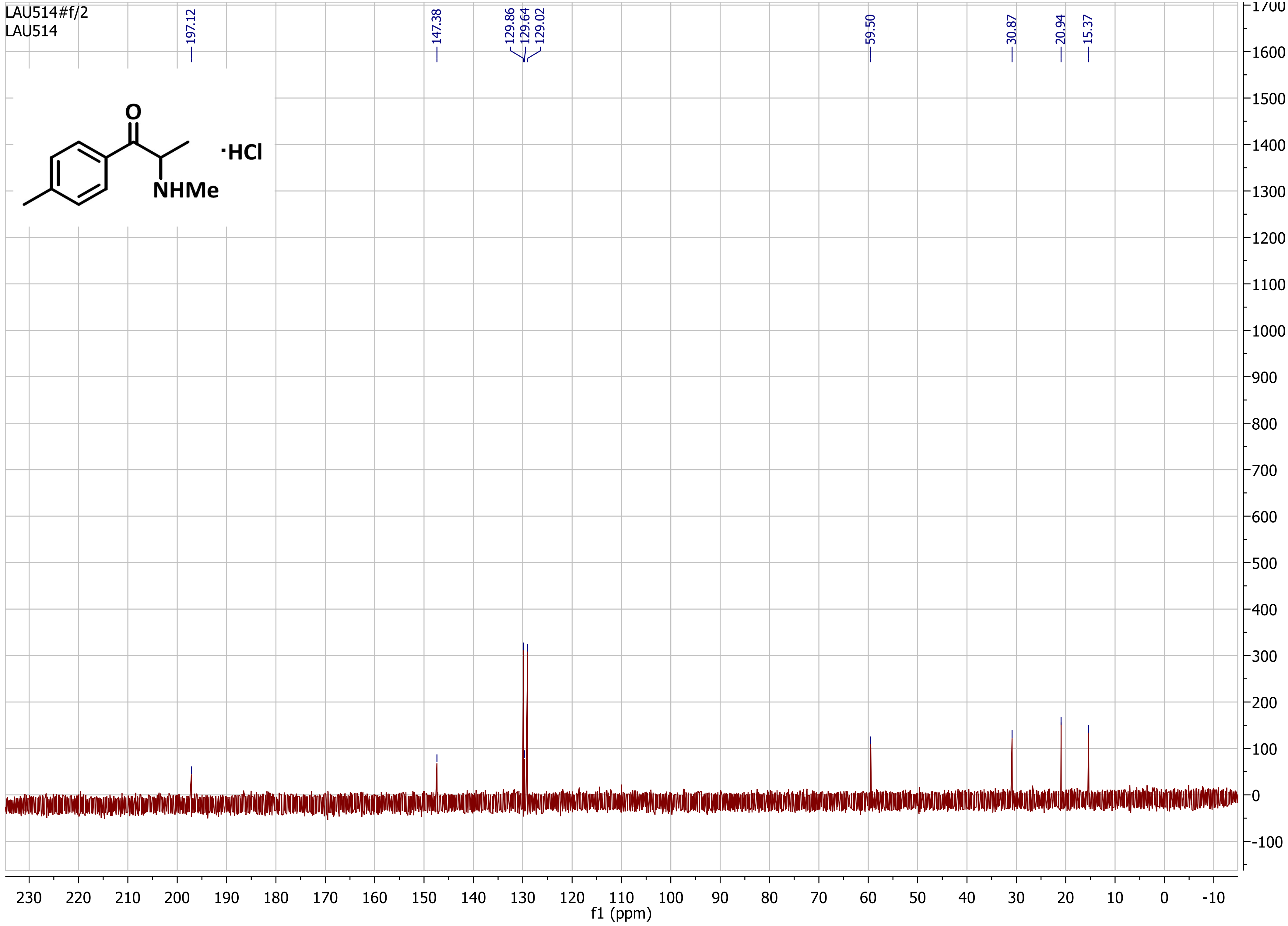
LAU514#f/2
LAU514
197.12
147.38
129.86
129.64
129.02
59.50
30.87
20.94
15.37
·HCl
O
NHMe
f1 (ppm)

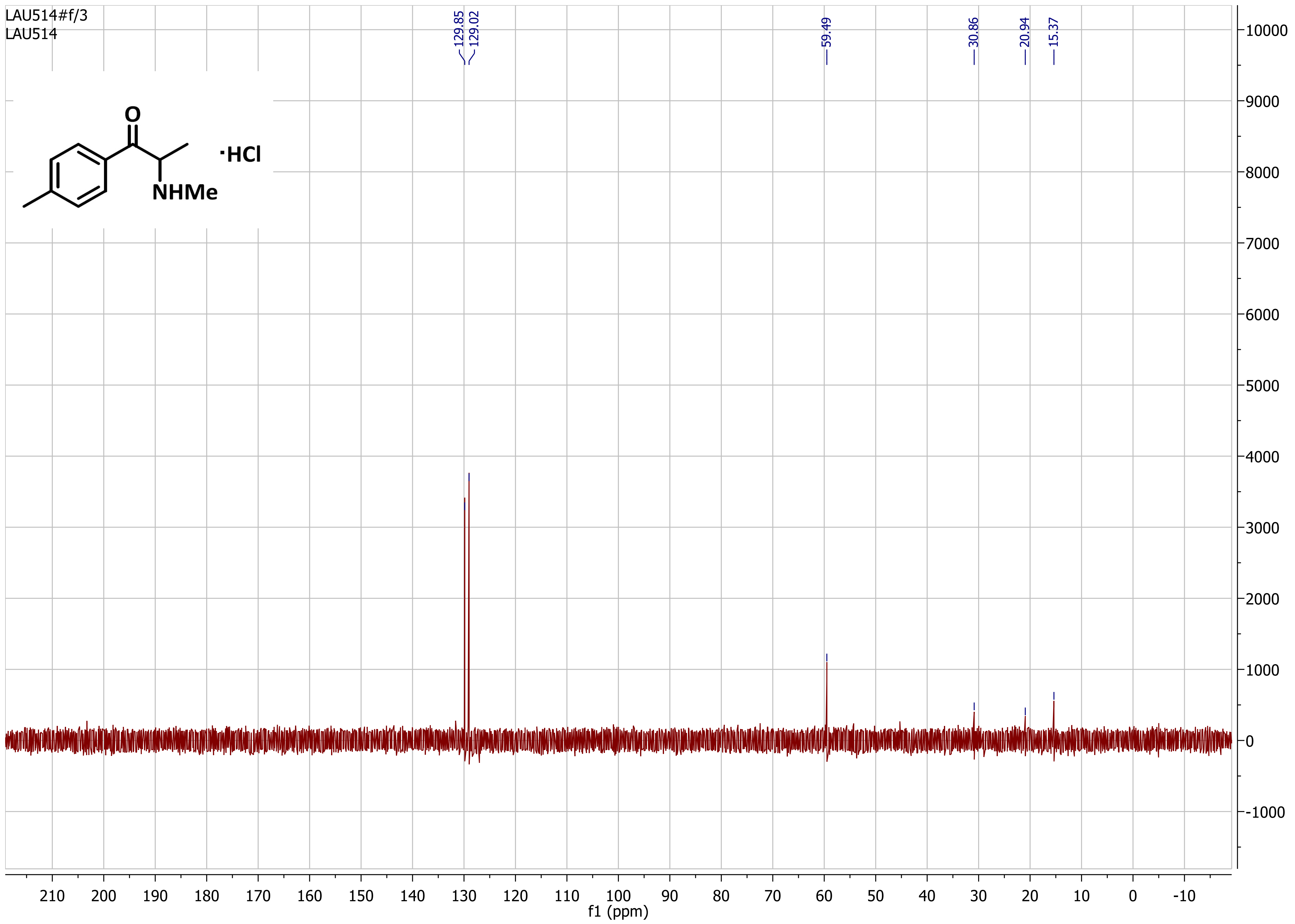
LAU514#f/3
LAU514
129.85
129.02
59.49
30.86
20.94
15.37
·HCl
NHMe
O
10000
9000
8000
7000
6000
5000
4000
3000
2000
1000
0
-1000
210
200
190
180
170
160
150
140
130
120
110
100
90
80
70
60
50
40
30
20
10
0
-10
f1 (ppm)

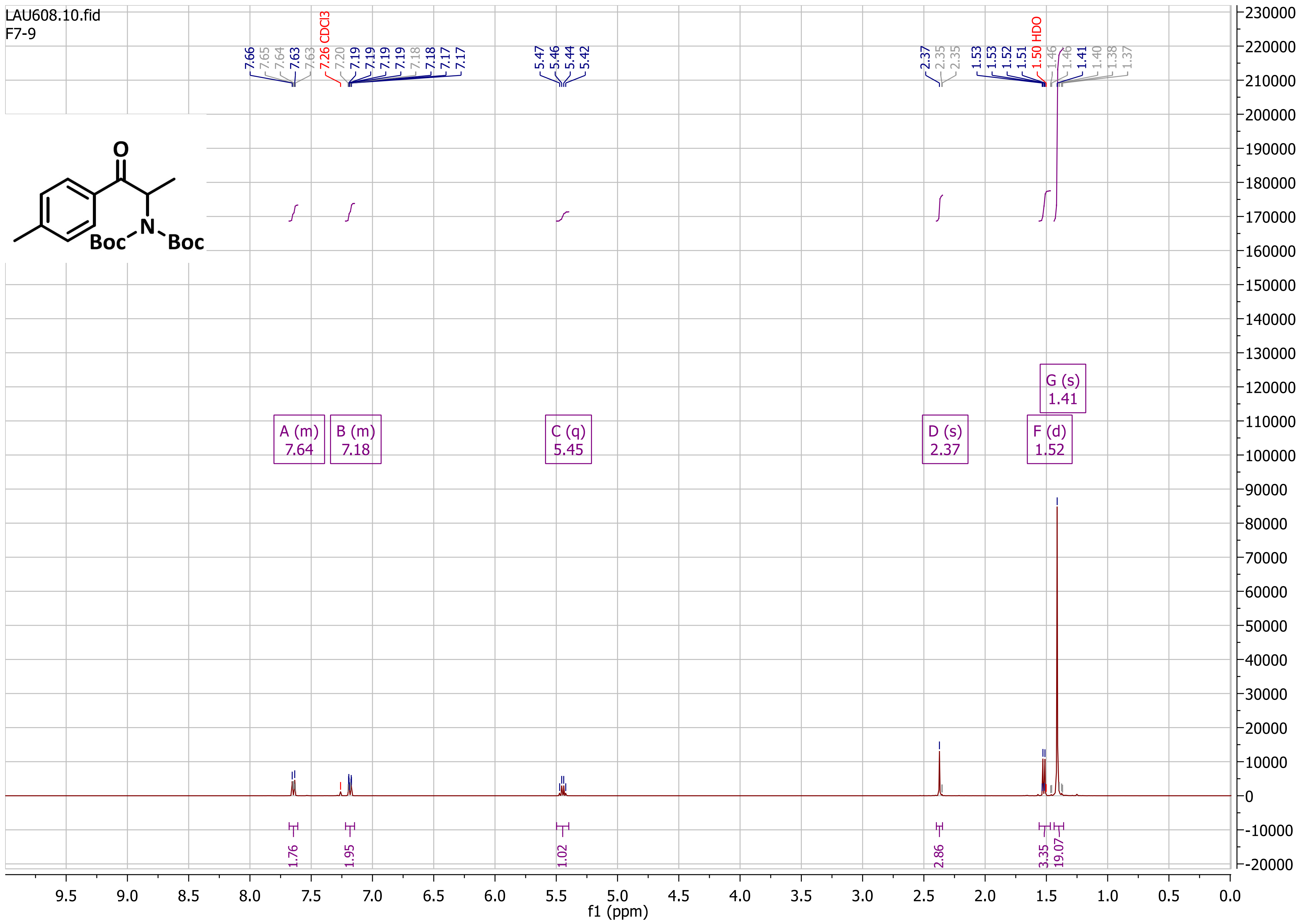
LAU608.10.fid
F7-9
7.66
7.65
7.64
7.63
7.63
7.26 CDCl3
7.20
7.19
7.19
7.19
7.19
7.18
7.18
7.17
7.17
5.47
5.46
5.44
5.42
2.37
2.35
2.35
1.53
1.53
1.52
1.51
1.50 HDO
1.46
1.46
1.41
1.40
1.38
1.37
O
N
Boc
Boc
A (m)
7.64
B (m)
7.18
C (q)
5.45
D (s)
2.37
F (d)
1.52
G (s)
1.41
1.76
1.95
1.02
2.86
3.35
19.07
f1 (ppm)

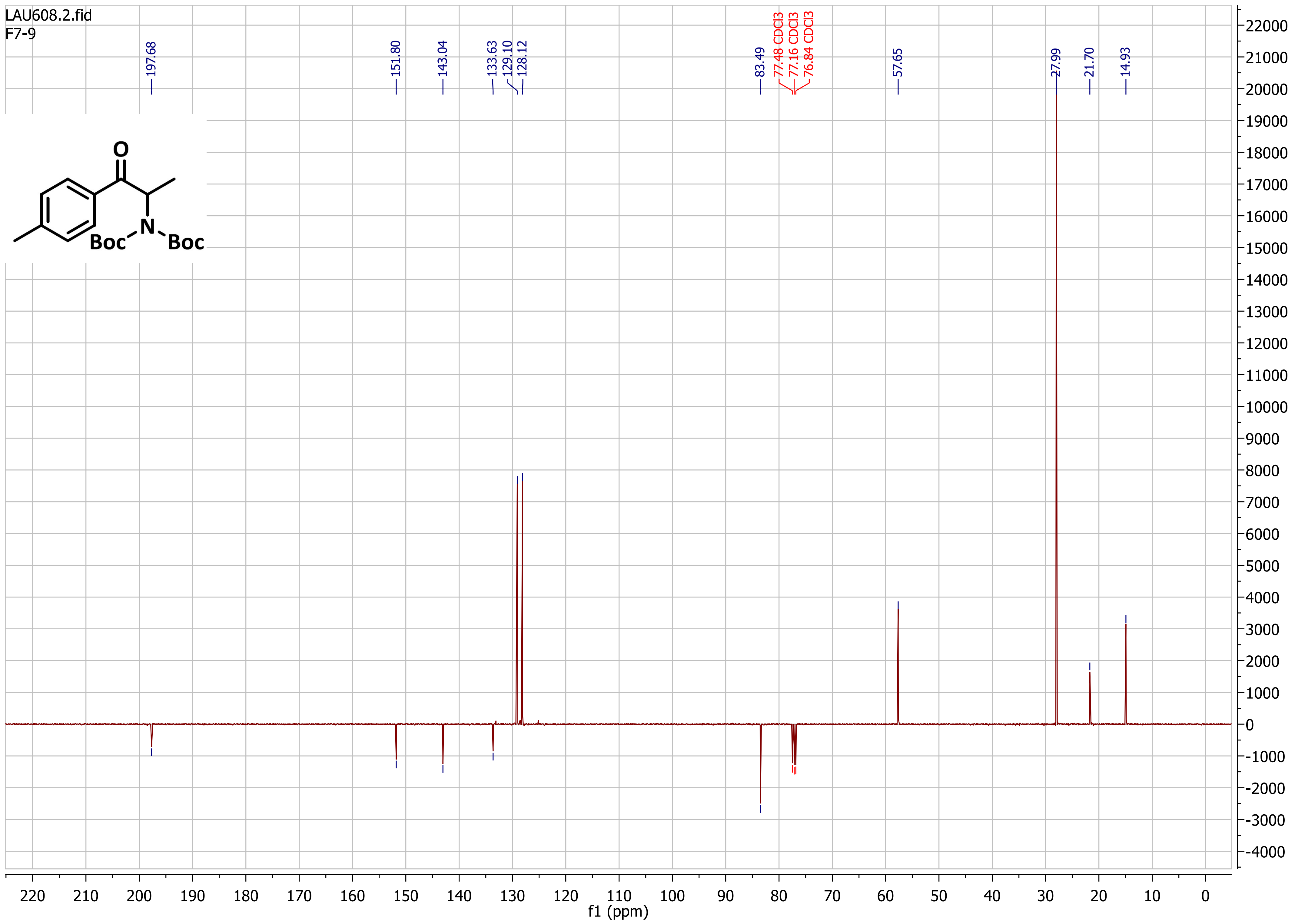
LAU608.2.fid
F7-9
197.68
151.80
143.04
133.63
129.10
128.12
83.49
77.48 CDCl3
77.16 CDCl3
76.84 CDCl3
57.65
27.99
21.70
14.93
O
N
Boc
Boc
f1 (ppm)

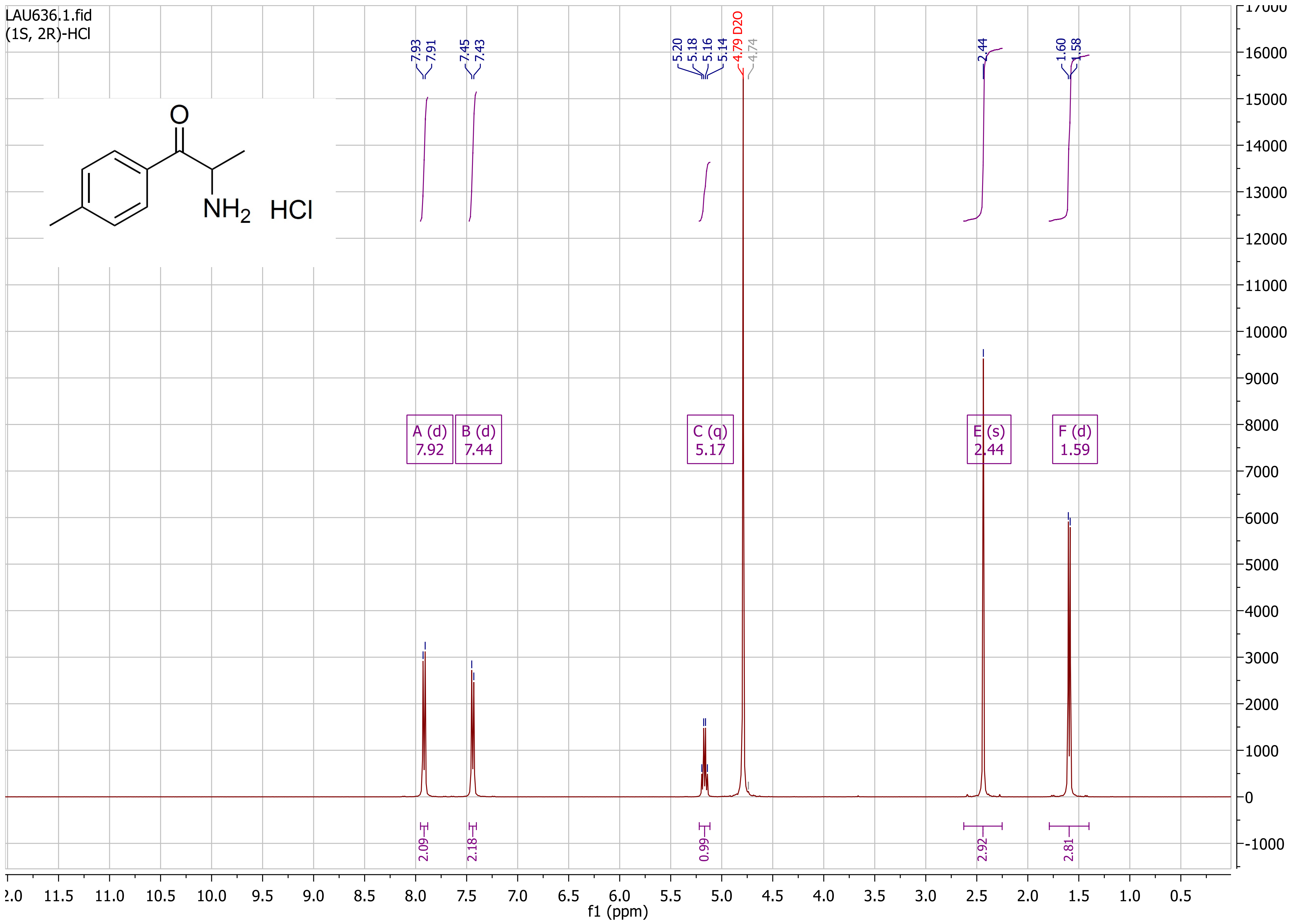

LAU636.1.fid
(1S, 2R)-HCl
NH2 HCl
7.93
7.91
7.45
7.43
5.20
5.18
5.16
5.14
4.79 D2O
4.74
2.44
1.60
1.58
A (d) 7.92
B (d) 7.44
C (q) 5.17
E (s) 2.44
F (d) 1.59
2.09
2.18
0.99
2.92
2.81
f1 (ppm)

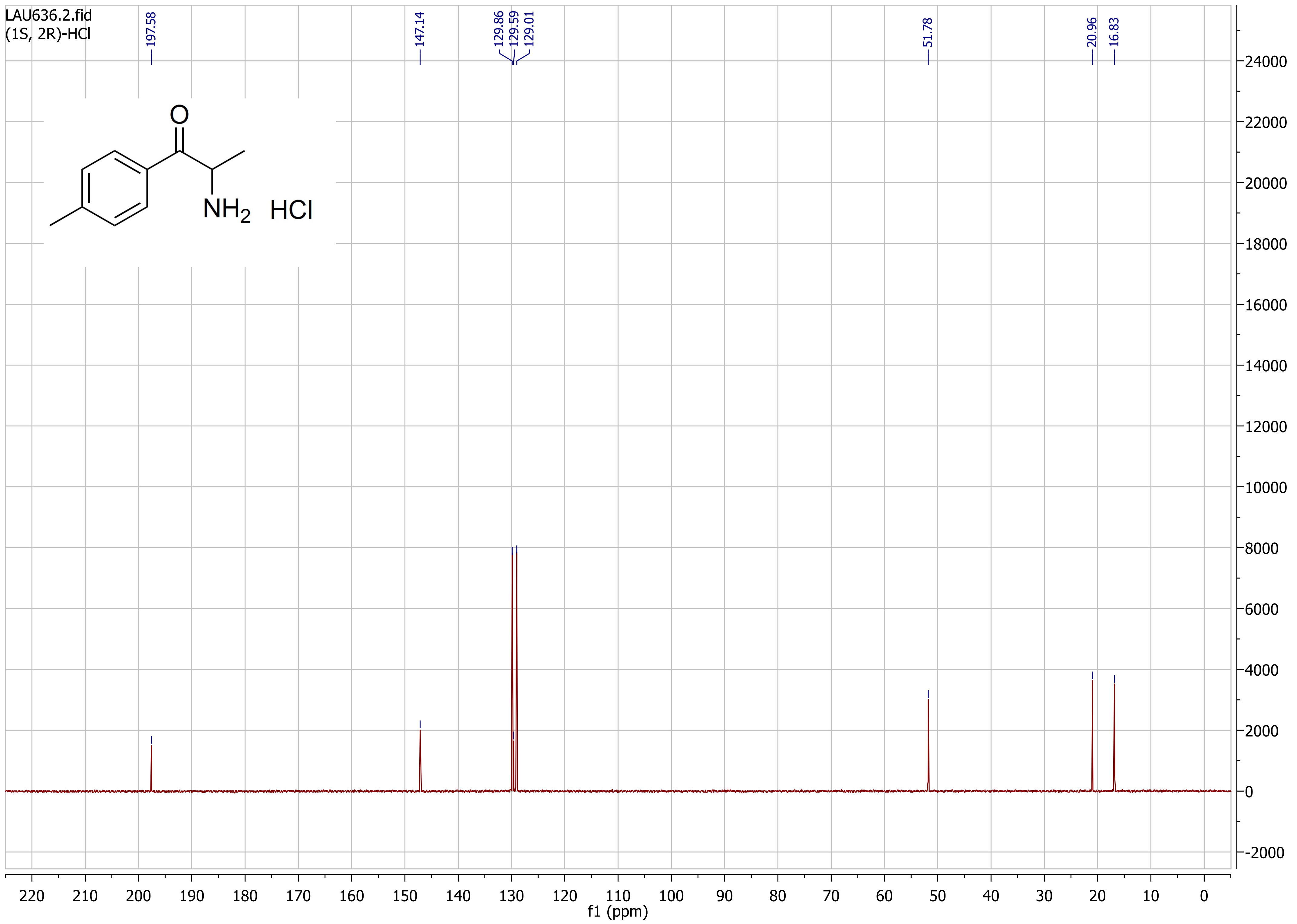
LAU636.2.fid
(1S, 2R)-HCl
197.58
147.14
129.86
129.59
129.01
51.78
20.96
16.83
O
NH2 HCl
24000
22000
20000
18000
16000
14000
12000
10000
8000
6000
4000
2000
0
-2000
220
210
200
190
180
170
160
150
140
130
120
110
100
90
80
70
60
50
40
30
20
10
0
f1 (ppm)

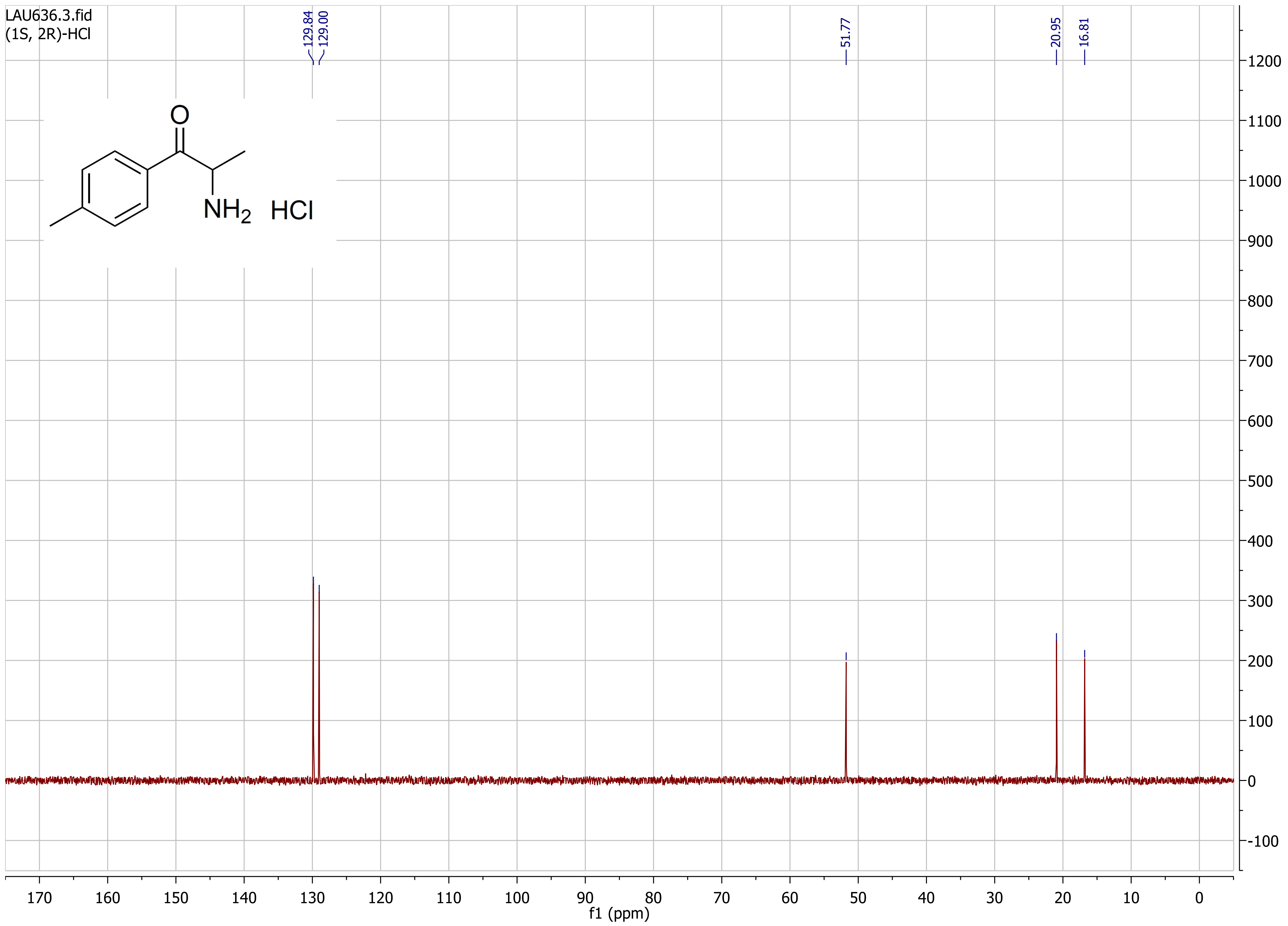
LAU636.3.fid
(1S, 2R)-HCl
129.84
129.00
51.77
20.95
16.81
NH2 HCl
O
f1 (ppm)

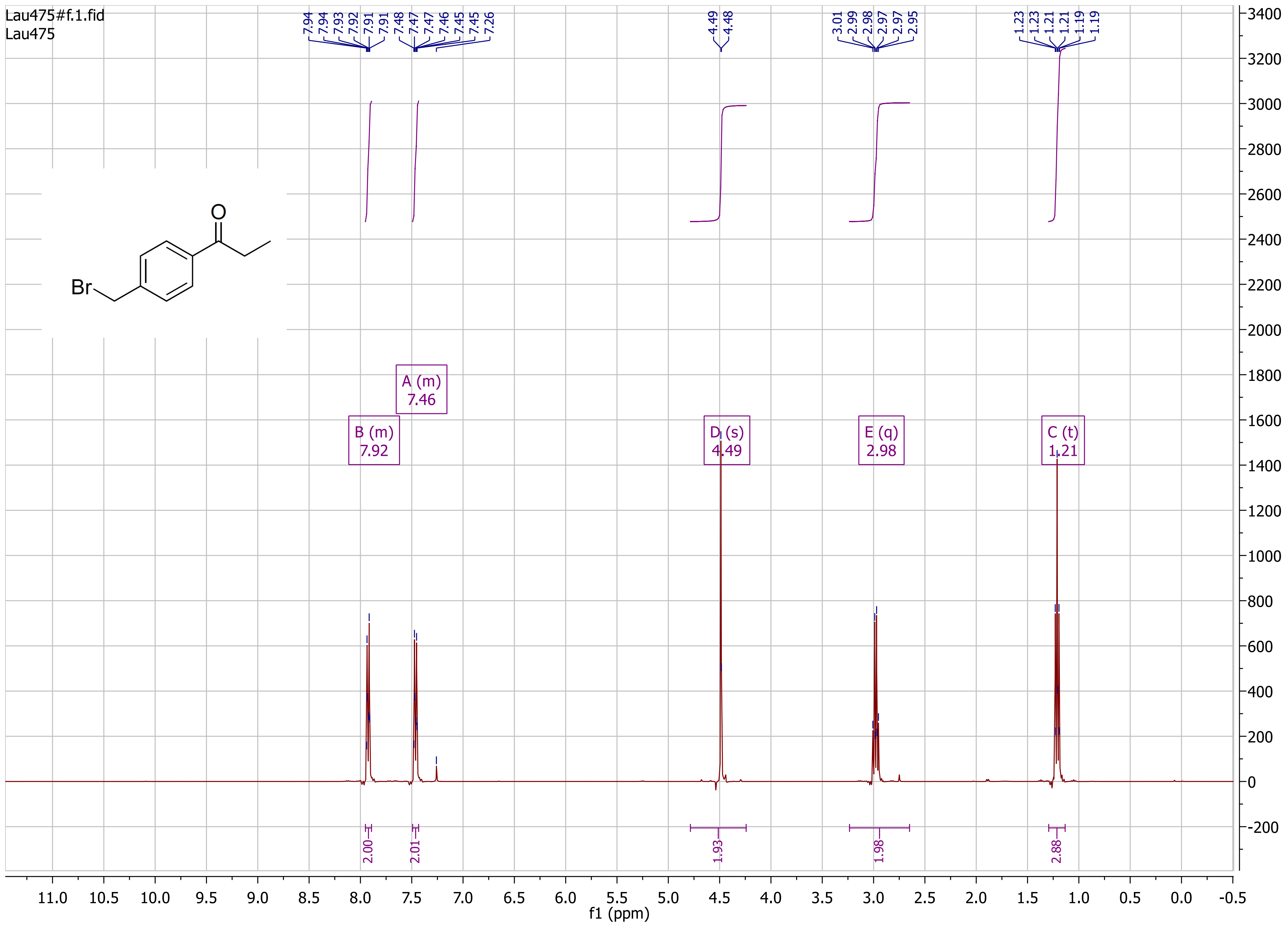

Lau475#f.1.fid
Lau475
7.94 7.94 7.93 7.92 7.91 7.91 7.48 7.47 7.47 7.46 7.45 7.45 7.26
4.49 4.48
3.01 2.99 2.98 2.97 2.97 2.95
1.23 1.23 1.21 1.21 1.19 1.19
Br
O
A (m)
7.46
B (m)
7.92
D (s)
4.49
E (q)
2.98
C (t)
1.21
2.00
2.01
1.93
1.98
2.88
f1 (ppm)

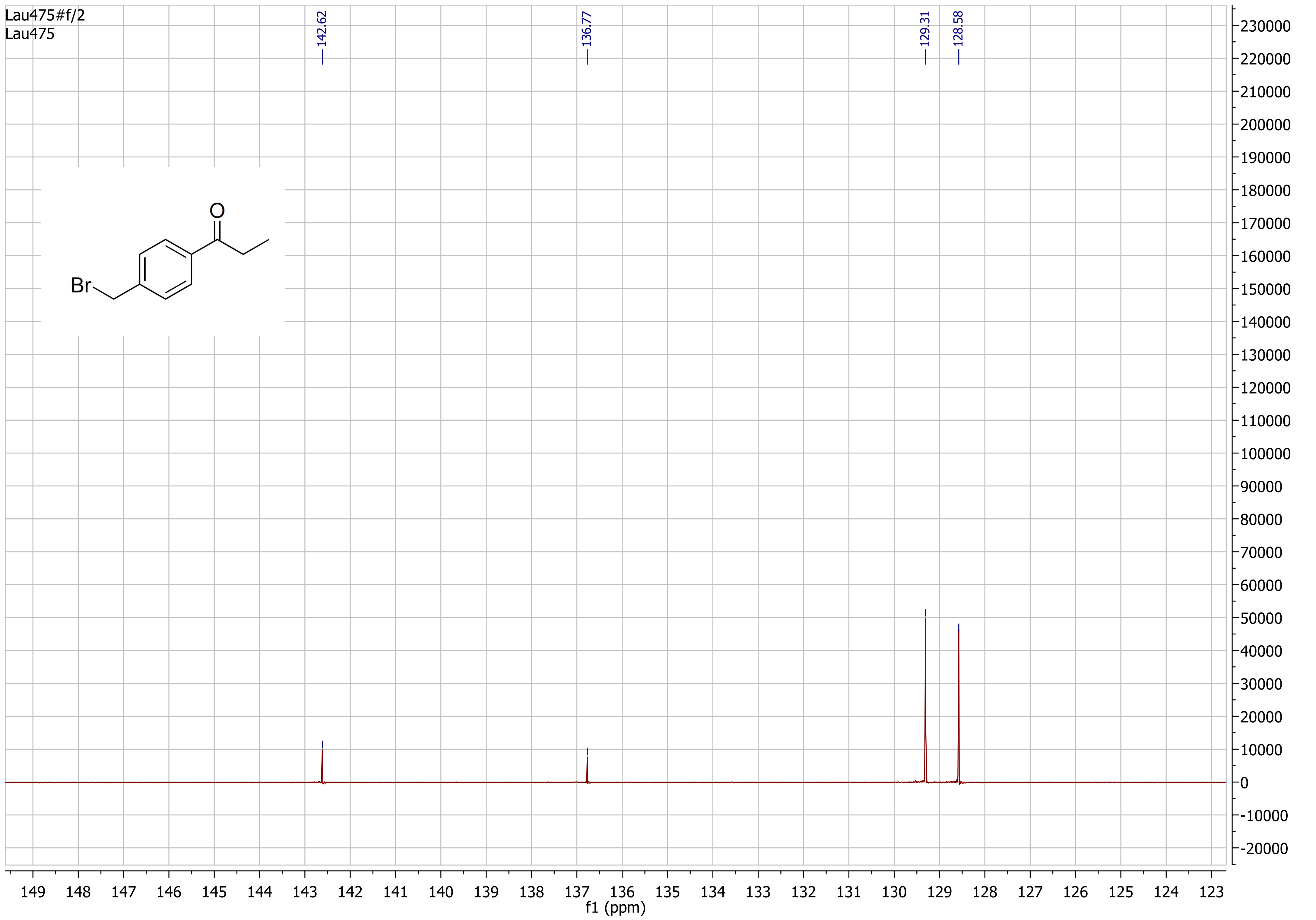
Lau475#f/2
Lau475
142.62
136.77
129.31
128.58
O
Br
f1 (ppm)
149 148 147 146 145 144 143 142 141 140 139 138 137 136 135 134 133 132 131 130 129 128 127 126 125 124 123
230000 220000 210000 200000 190000 180000 170000 160000 150000 140000 130000 120000 110000 100000 90000 80000 70000 60000 50000 40000 30000 20000 10000 0 -10000 -20000

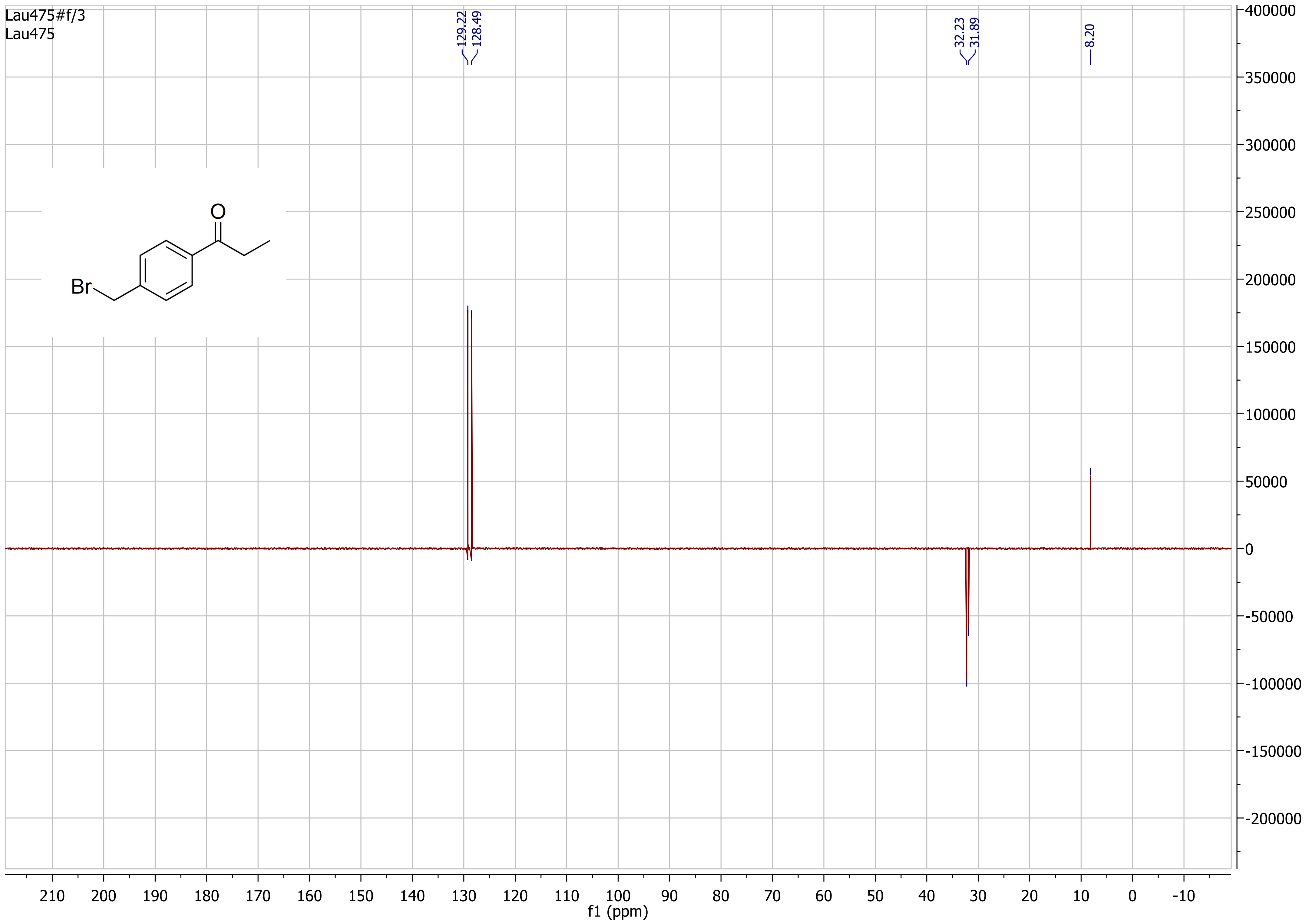

Lau475#f/3
Lau475
129.22
128.49
32.23
31.89
8.20
Br
O
400000
350000
300000
250000
200000
150000
100000
50000
0
-50000
-100000
-150000
-200000
210
200
190
180
170
160
150
140
130
120
110
100
90
80
70
60
50
40
30
20
10
0
-10
f1 (ppm)

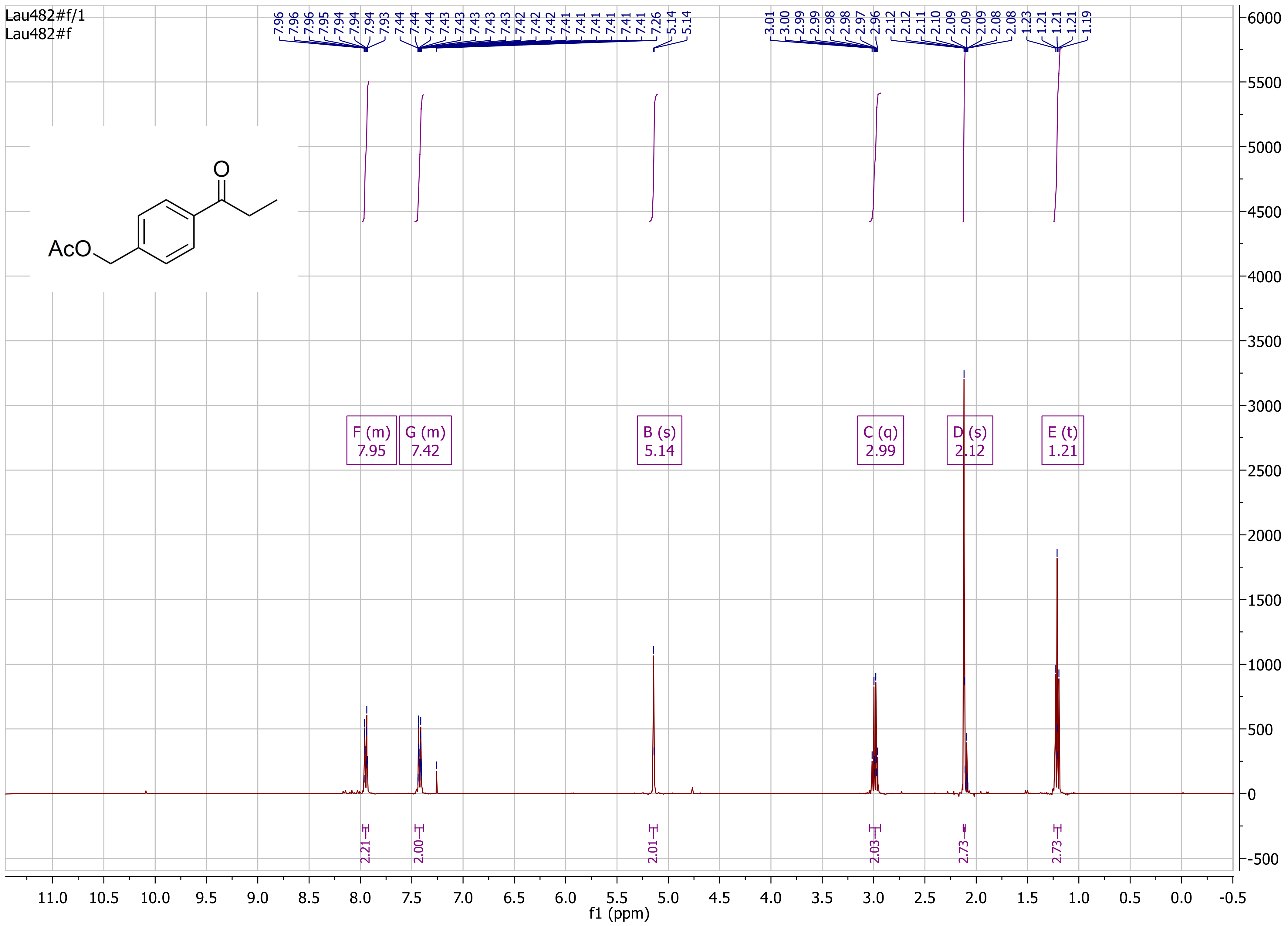
Lau482#f/1
Lau482#f
AcO
O
7.96 7.96 7.96 7.95 7.94 7.94 7.94 7.93 7.44 7.44 7.44 7.43 7.43 7.43 7.43 7.43 7.42 7.42 7.42 7.41 7.41 7.41 7.41 7.41 7.41 7.26 5.14 5.14
3.01 3.00 2.99 2.99 2.98 2.98 2.97 2.96 2.12 2.12 2.11 2.10 2.09 2.09 2.09 2.08 2.08 1.23 1.21 1.21 1.21 1.19
F (m) 7.95
G (m) 7.42
B (s) 5.14
C (q) 2.99
D (s) 2.12
E (t) 1.21
2.21
2.00
2.01
2.03
2.73
2.73
f1 (ppm)

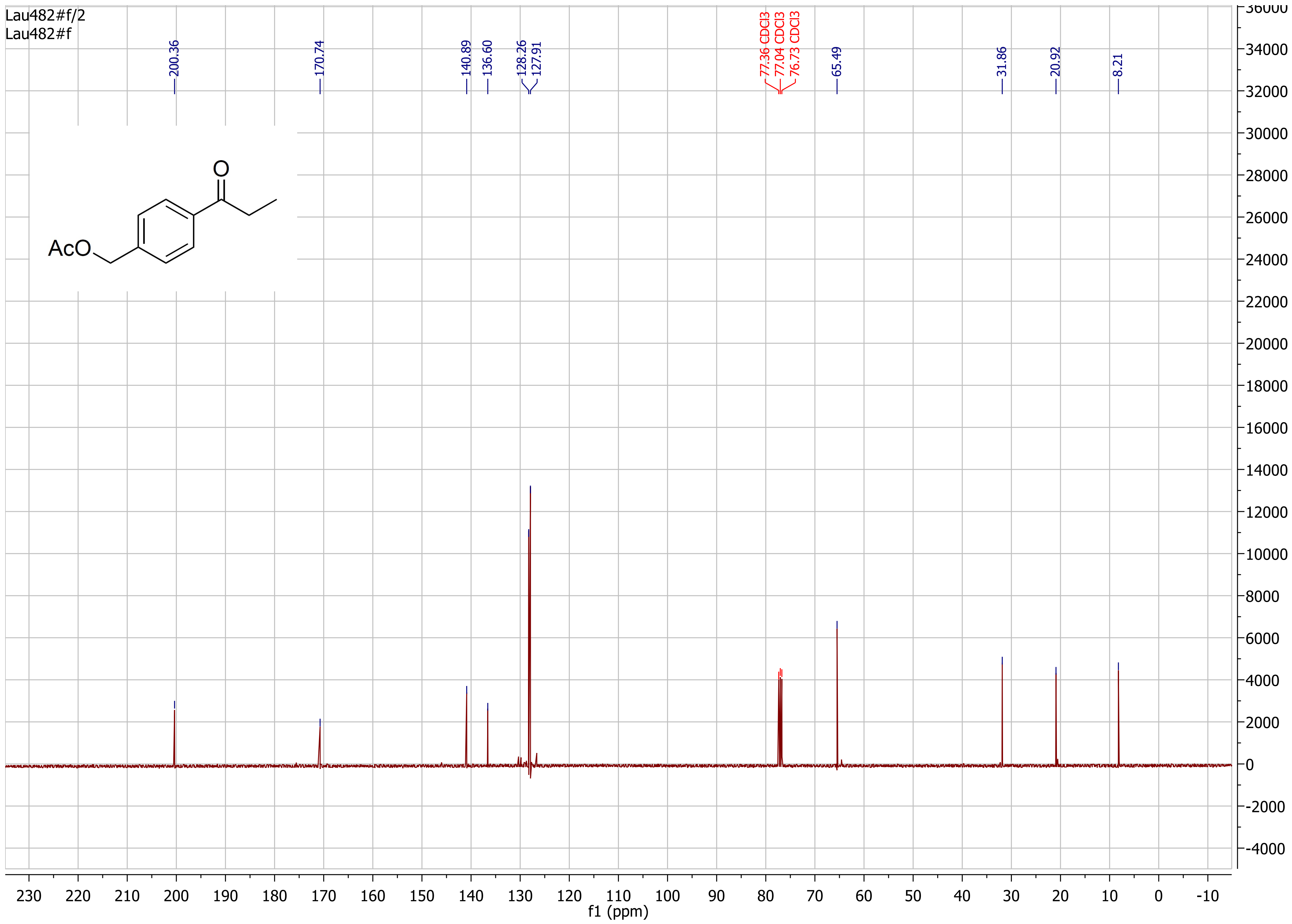

Lau482#f/2
Lau482#f
200.36
170.74
140.89
136.60
128.26
127.91
77.36 CDCl3
77.04 CDCl3
76.73 CDCl3
65.49
31.86
20.92
8.21
AcO
O
f1 (ppm)

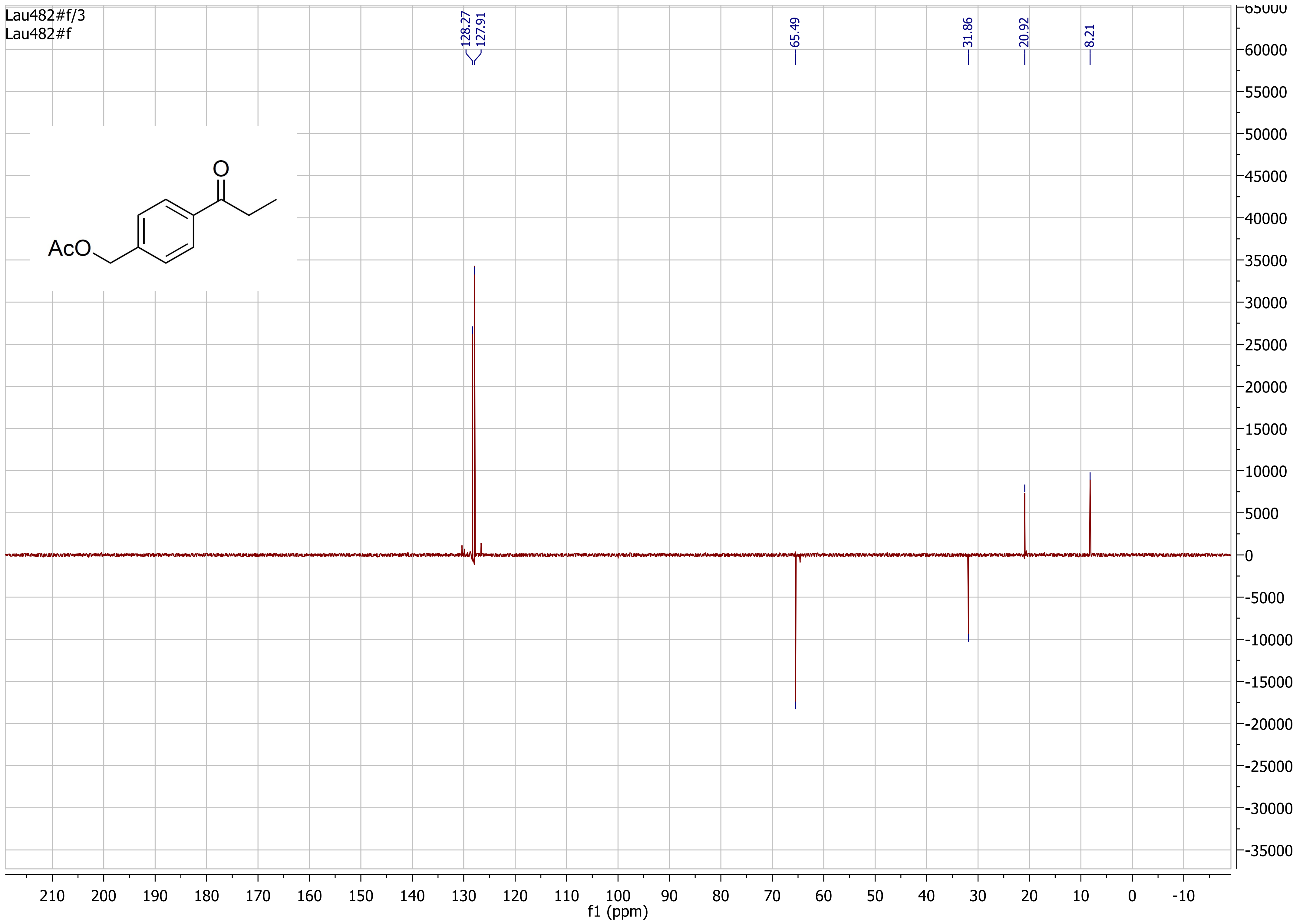
Lau482#f/3
Lau482#f
128.27
127.91
65.49
31.86
20.92
8.21
AcO
O
f1 (ppm)
210 200 190 180 170 160 150 140 130 120 110 100 90 80 70 60 50 40 30 20 10 0 -10
65000 60000 55000 50000 45000 40000 35000 30000 25000 20000 15000 10000 5000 0 -5000 -10000 -15000 -20000 -25000 -30000 -35000

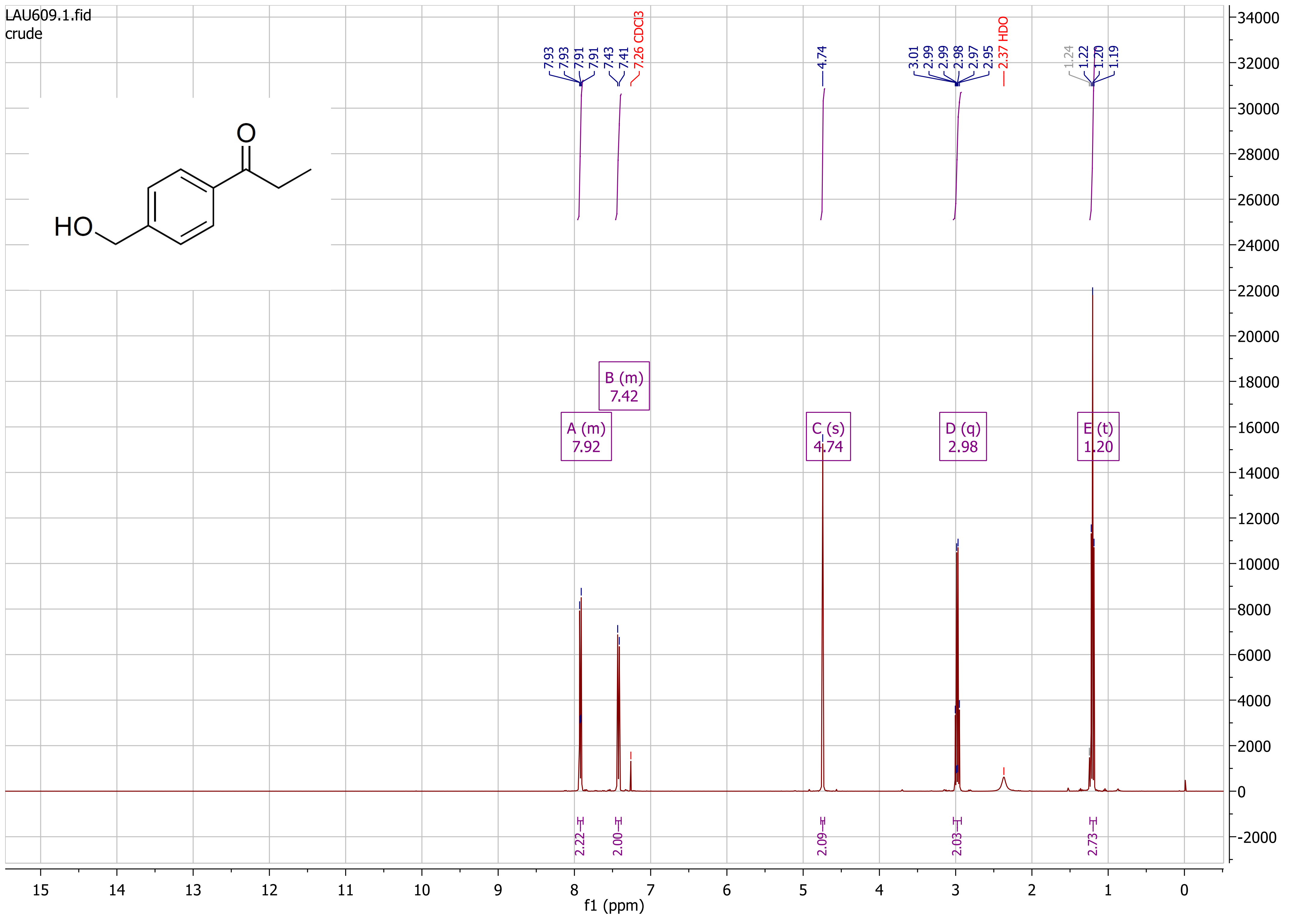

LAU609.1.fid
crude
HO
O
7.93
7.93
7.91
7.91
7.43
7.41
7.26 CDCl3
4.74
3.01
2.99
2.99
2.98
2.97
2.95
2.37 HDO
1.24
1.22
1.20
1.19
A (m)
7.92
B (m)
7.42
C (s)
4.74
D (q)
2.98
E (t)
1.20
2.22
2.00
2.09
2.03
2.73
f1 (ppm)

LAU609.2.fid
crude

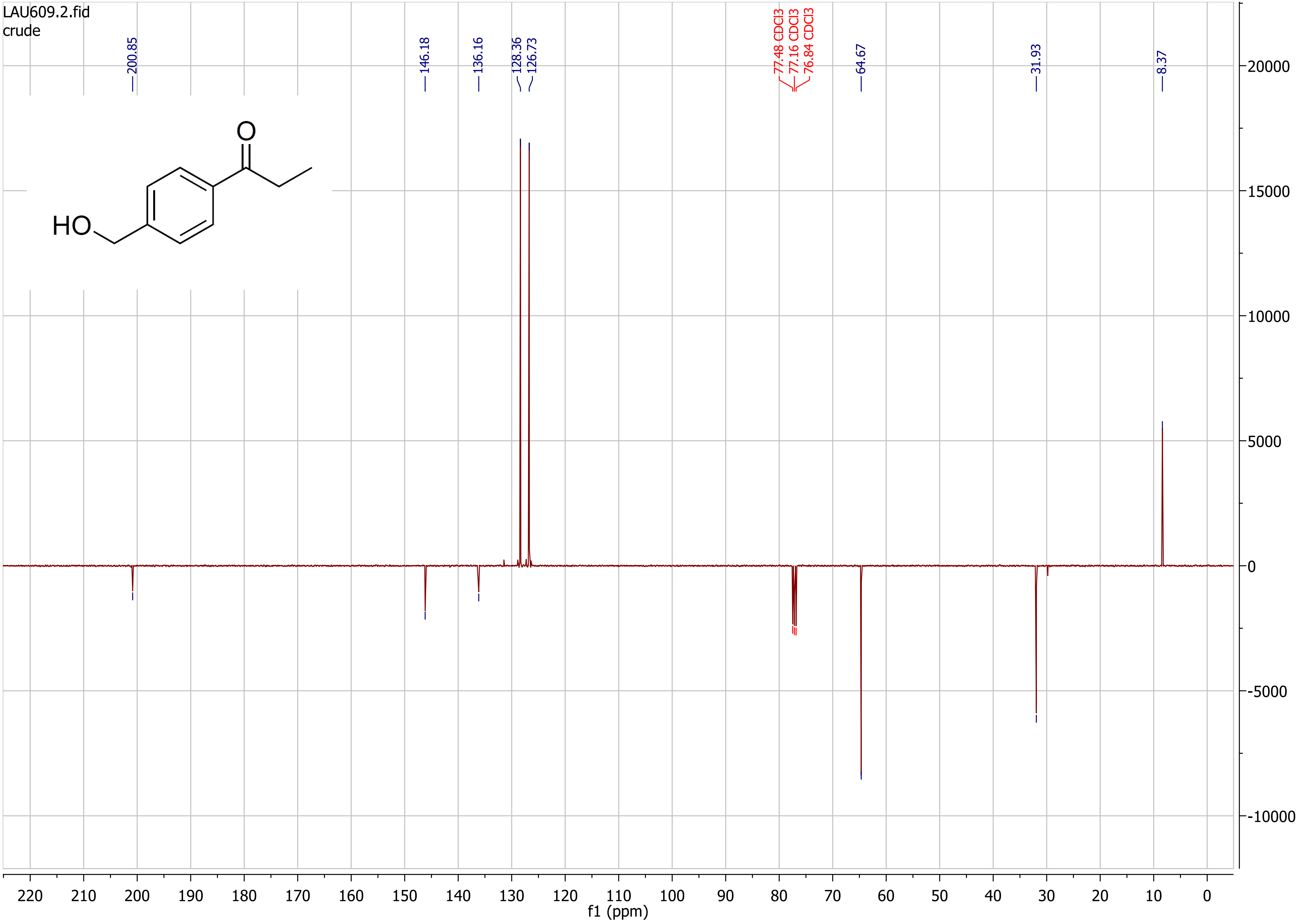

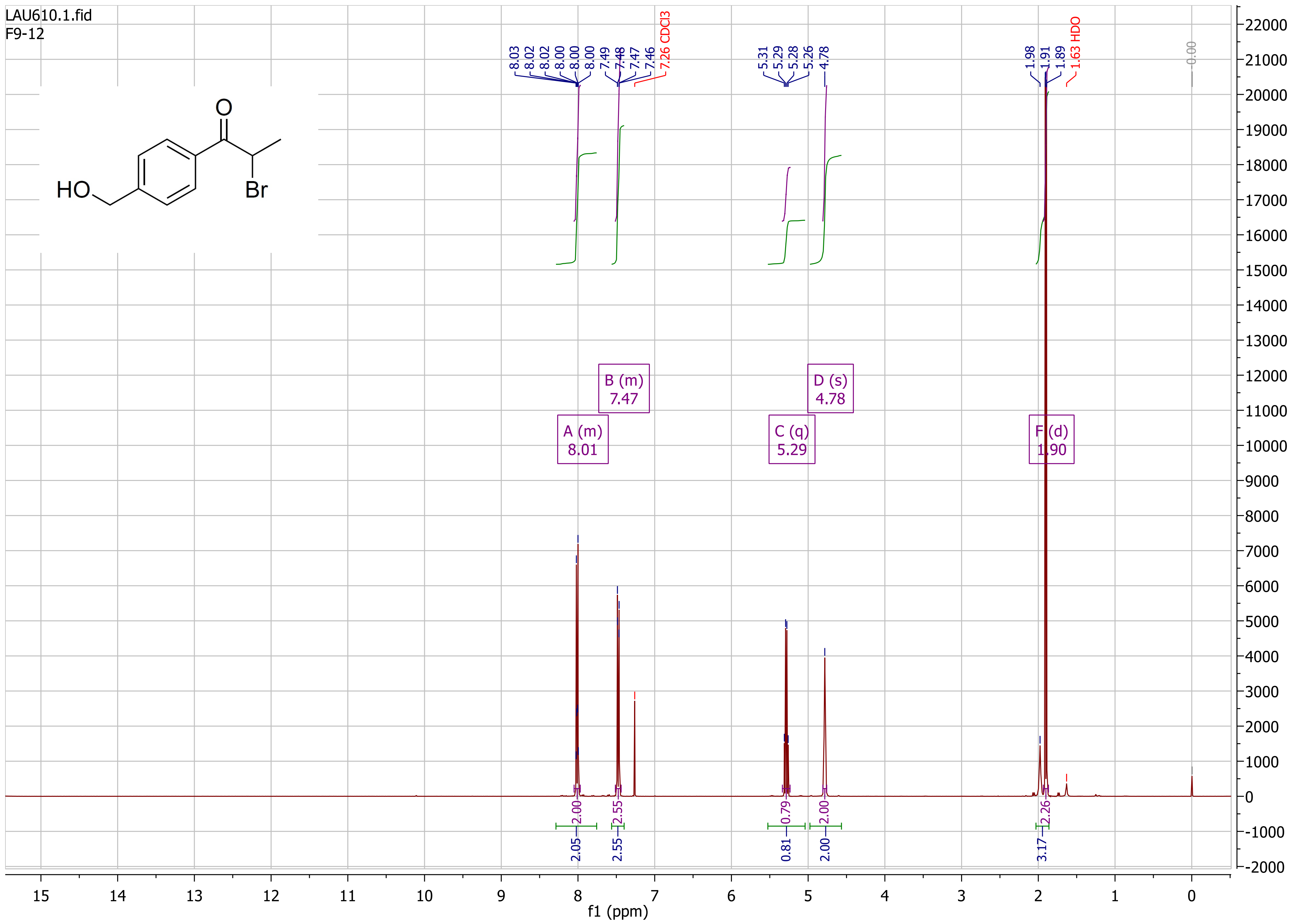
LAU610.1.fid
F9-12
HO
O
Br
8.03
8.02
8.02
8.00
8.00
8.00
7.49
7.48
7.47
7.46
7.26 CDCl3
5.31
5.29
5.28
5.26
4.78
1.98
1.91
1.89
1.63 HDO
0.00
A (m)
8.01
B (m)
7.47
C (q)
5.29
D (s)
4.78
F (d)
1.90
2.00
2.05
2.55
2.55
0.79
0.81
2.00
2.00
2.26
3.17
f1 (ppm)

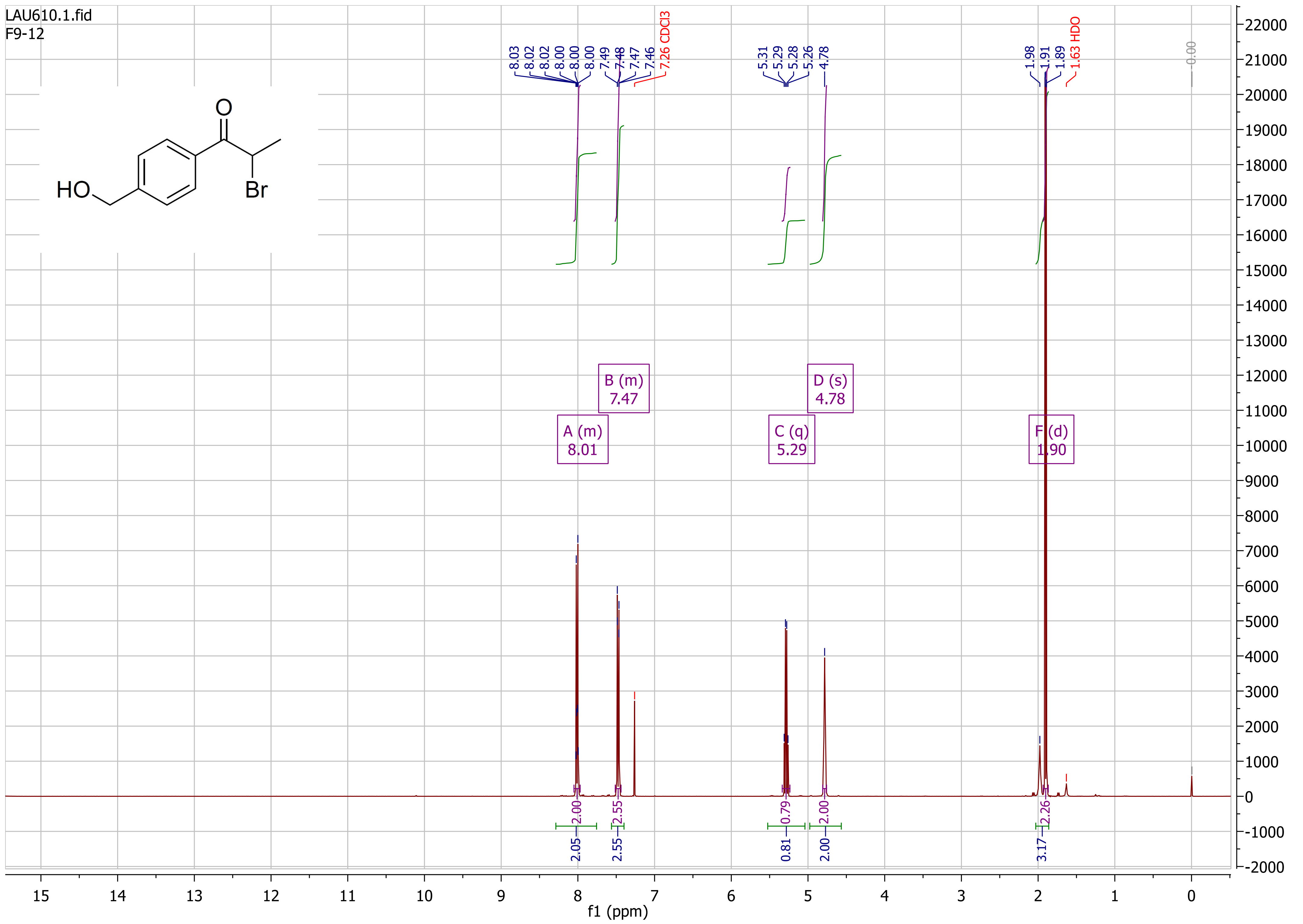

LAU610.1.fid
F9-12
HO
O
Br
8.03
8.02
8.02
8.00
8.00
8.00
7.49
7.48
7.47
7.46
7.26 CDCl3
5.31
5.29
5.28
5.26
4.78
1.98
1.91
1.89
1.63 HDO
0.00
B (m)
7.47
D (s)
4.78
A (m)
8.01
C (q)
5.29
F (d)
1.90
2.00
2.55
0.79
2.00
2.26
2.05
2.55
0.81
2.00
3.17
f1 (ppm)

LAU610.2.fid
9-12

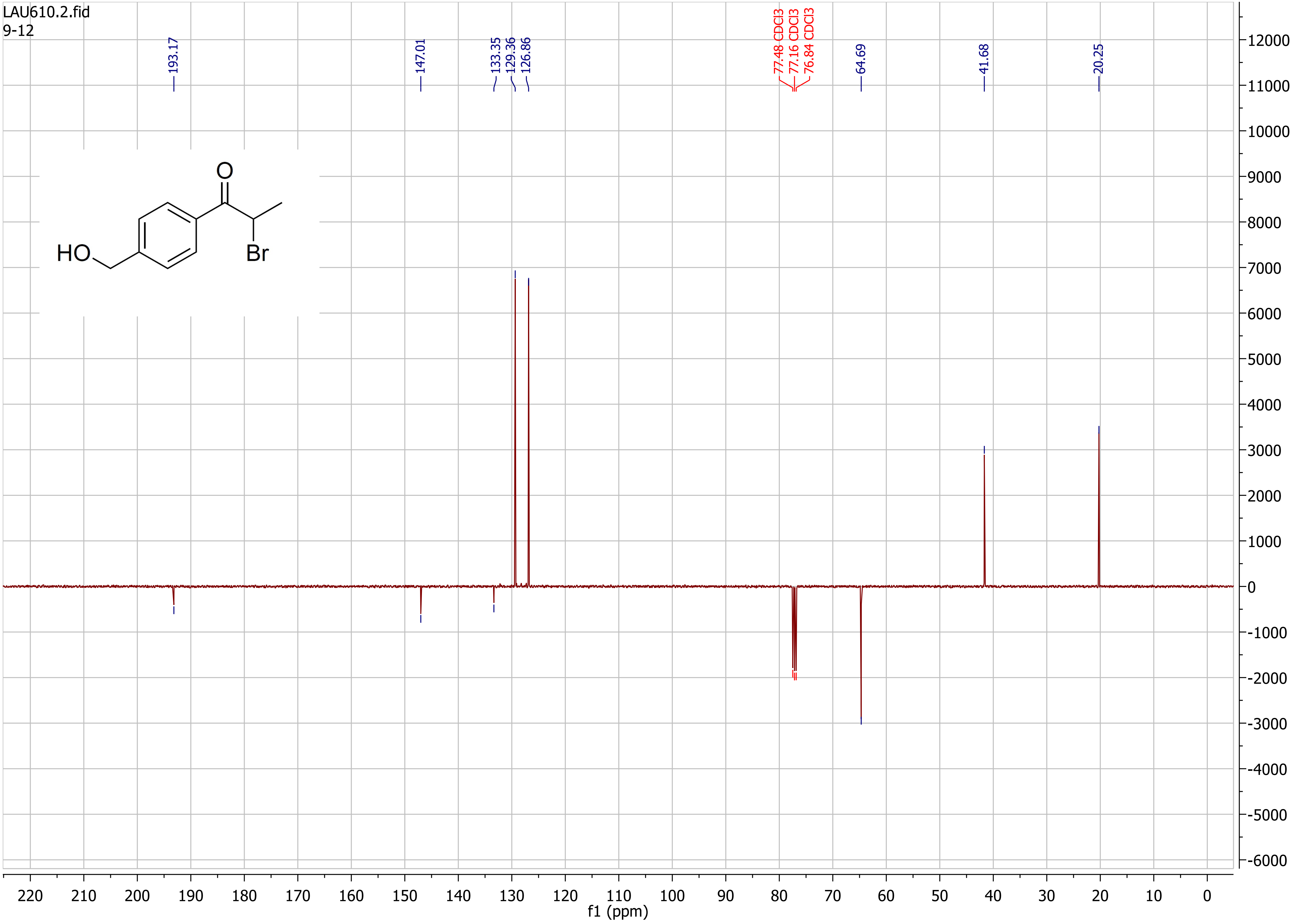

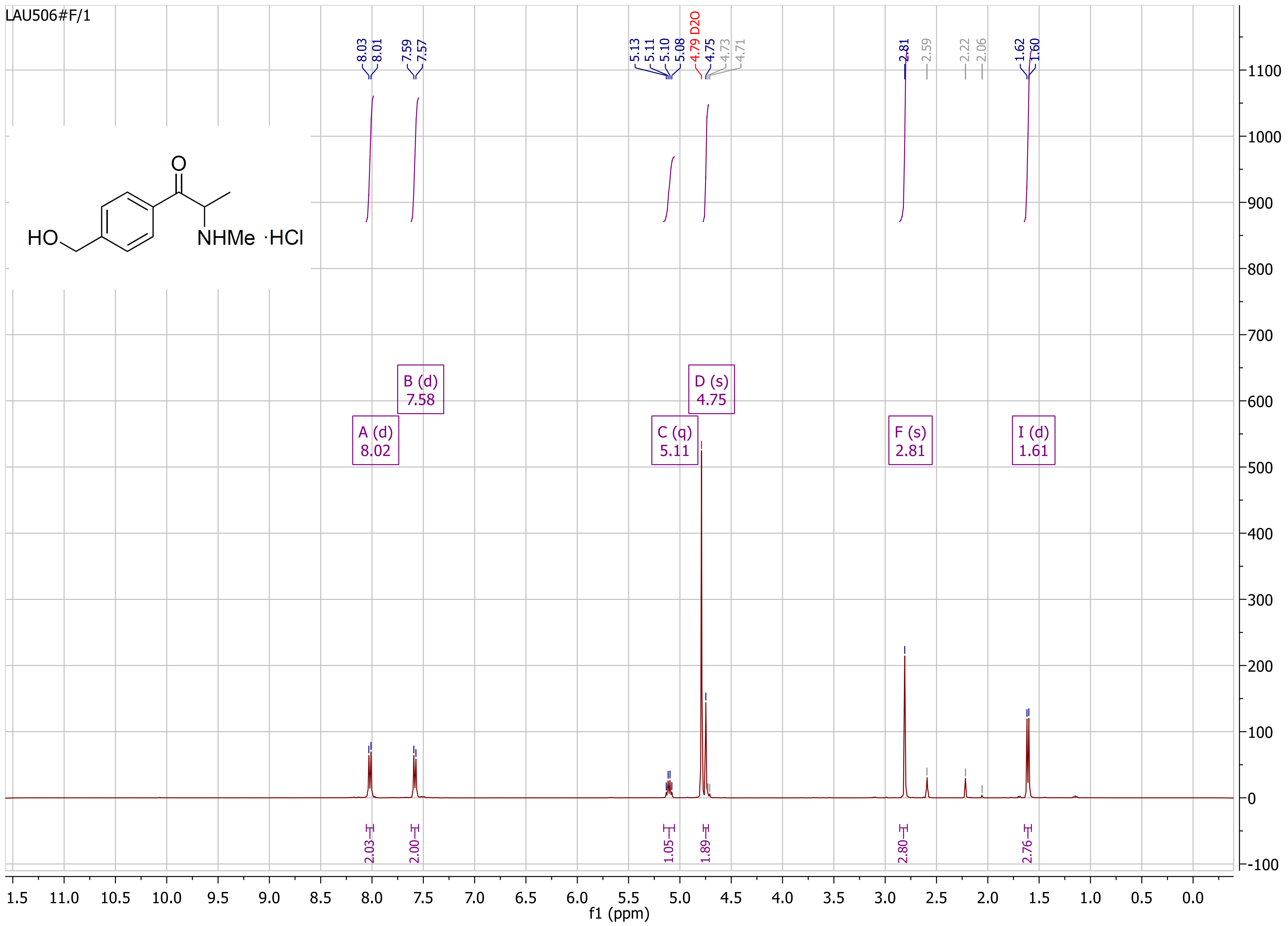

LAU506#F/1
HO
O
NHMe ·HCl
8.03
8.01
7.59
7.57
5.13
5.11
5.10
5.08
4.79 D2O
4.75
4.73
4.71
2.81
2.59
2.22
2.06
1.62
1.60
A (d) 8.02
B (d) 7.58
C (q) 5.11
D (s) 4.75
F (s) 2.81
I (d) 1.61
2.03
2.00
1.05
1.89
2.80
2.76
f1 (ppm)

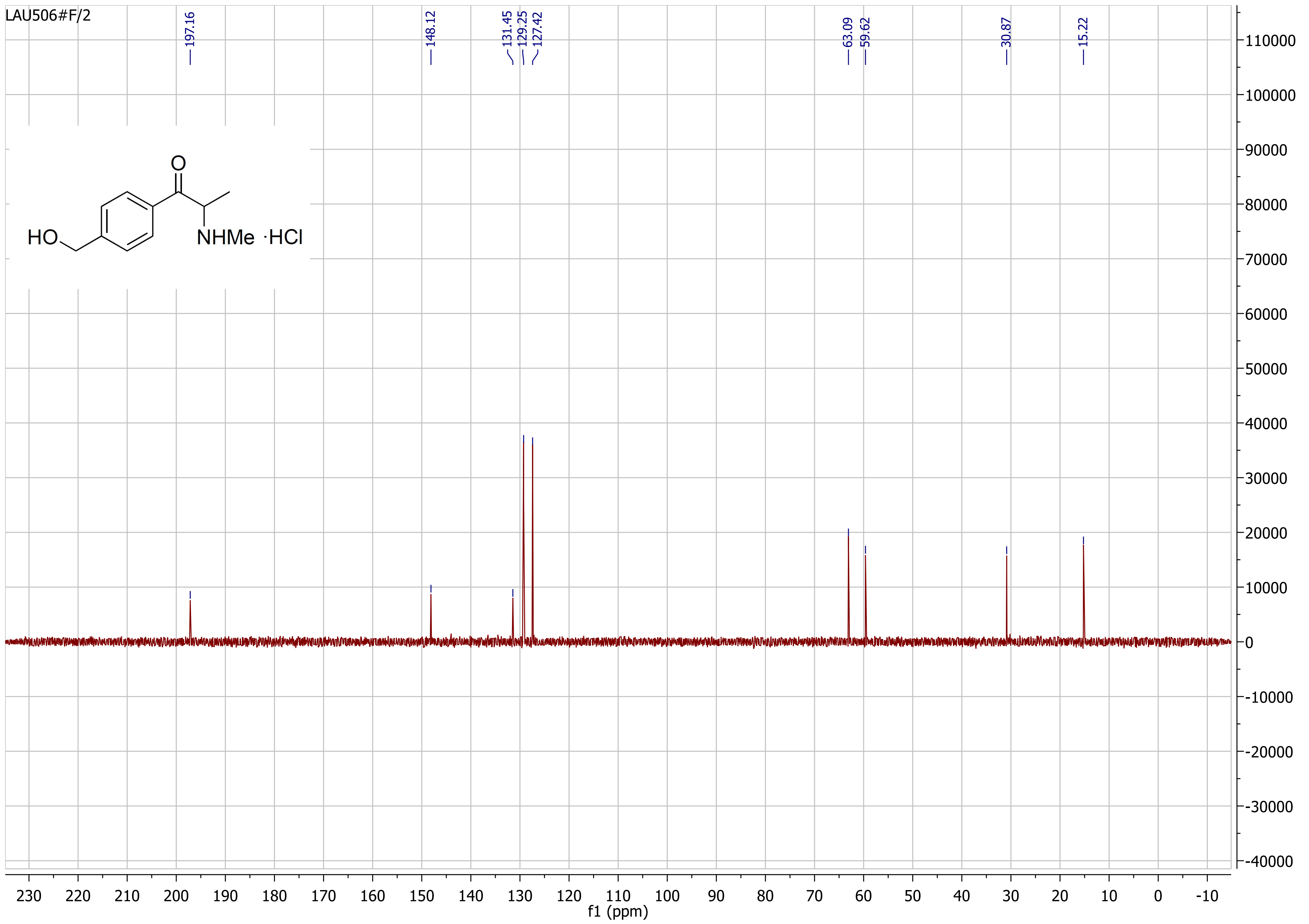
LAU506#F/2
197.16
148.12
131.45
129.25
127.42
63.09
59.62
30.87
15.22
HO
O
NHMe ·HCl
f1 (ppm)

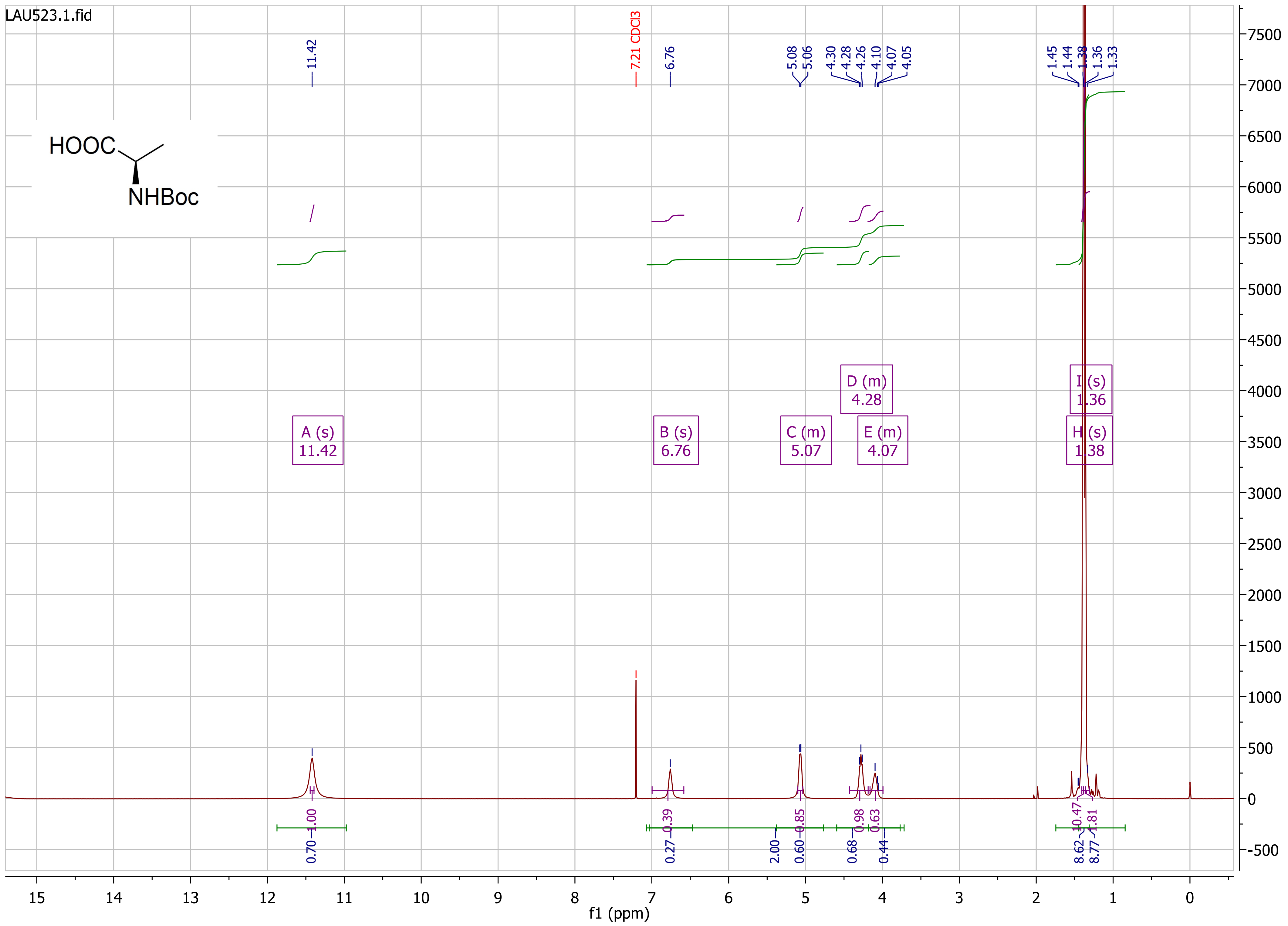

LAU523.1.fid
HOOC
NHBoc
11.42
7.21 CDCl3
6.76
5.08
5.06
4.30
4.28
4.26
4.10
4.07
4.05
1.45
1.44
1.38
1.36
1.33
A (s)
11.42
B (s)
6.76
C (m)
5.07
D (m)
4.28
E (m)
4.07
I (s)
1.36
H (s)
1.38
1.00
0.70
0.39
0.27
2.00
0.85
0.60
0.98
0.68
0.63
0.44
10.47
8.62
1.81
8.77
f1 (ppm)

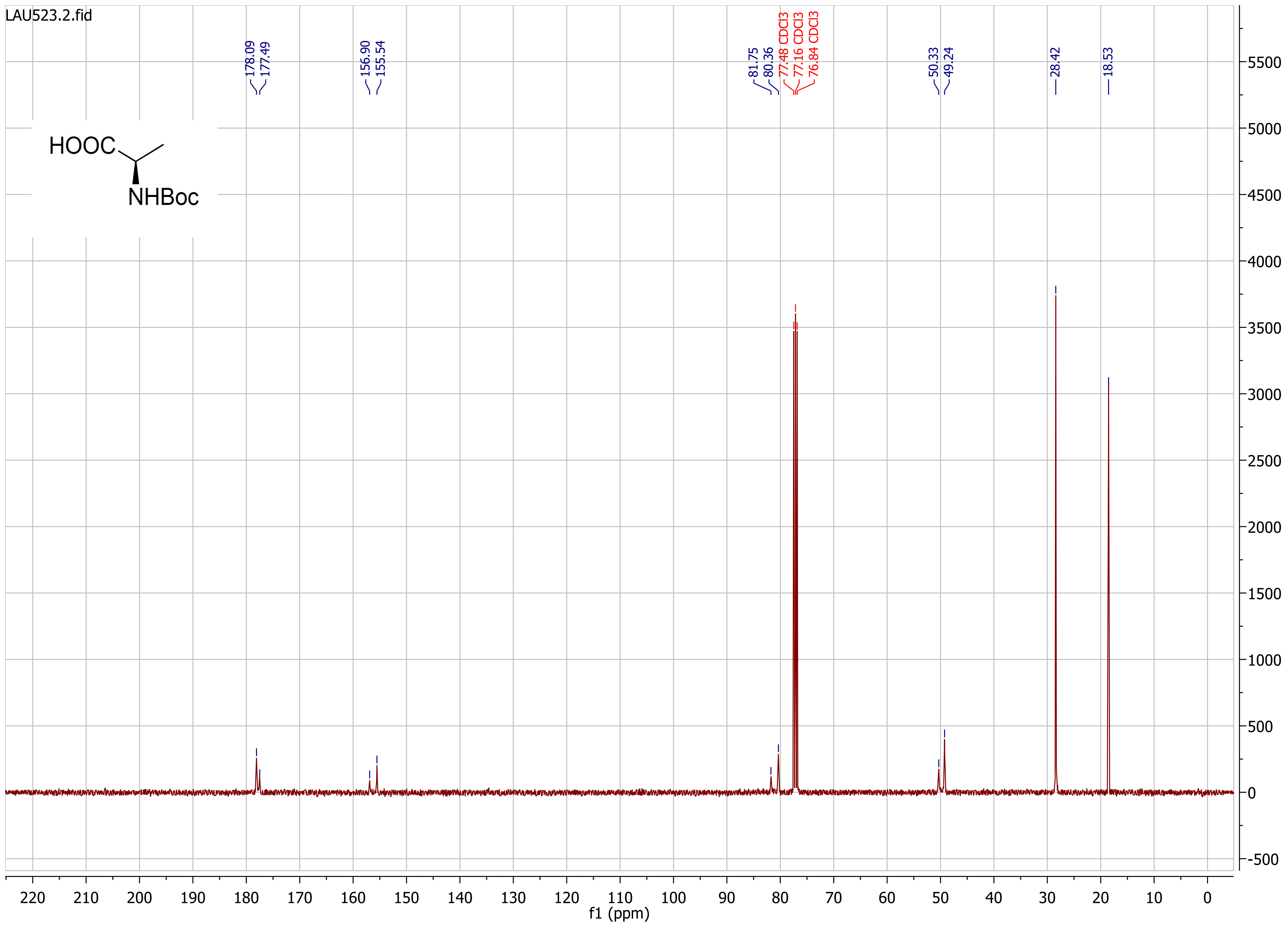
LAU523.2.fid
178.09
177.49
156.90
155.54
81.75
80.36
77.48 CDCl3
77.16 CDCl3
76.84 CDCl3
50.33
49.24
28.42
18.53
HOOC
NHBoc
f1 (ppm)

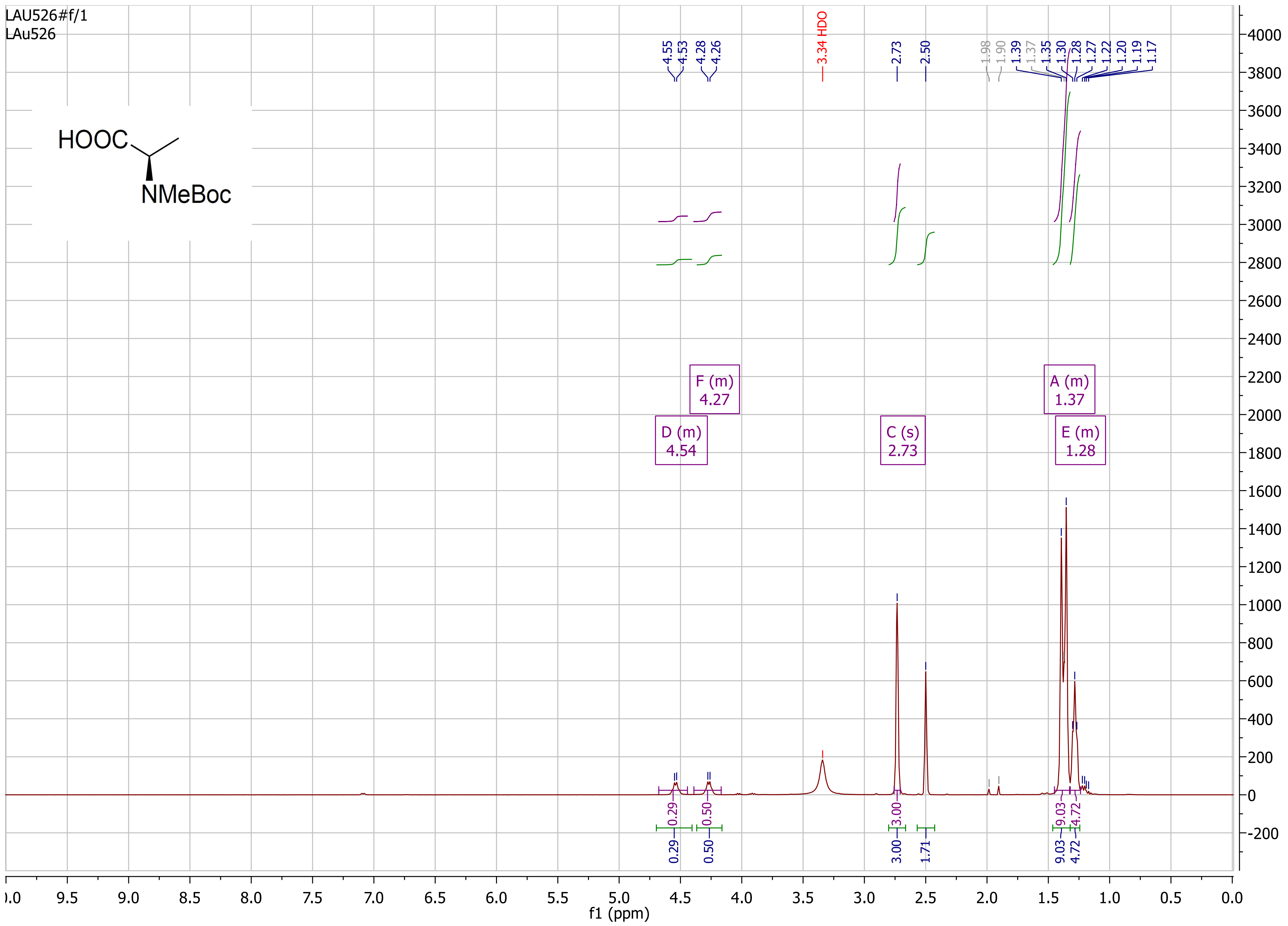
LAU526#f/1
LAu526
HOOC
NMeBoc
4.55
4.53
4.28
4.26
3.34 HDO
2.73
2.50
1.98
1.90
1.39
1.37
1.35
1.30
1.28
1.27
1.22
1.20
1.19
1.17
F (m) 4.27
D (m) 4.54
C (s) 2.73
A (m) 1.37
E (m) 1.28
0.29
0.50
3.00
1.71
9.03
4.72
f1 (ppm)

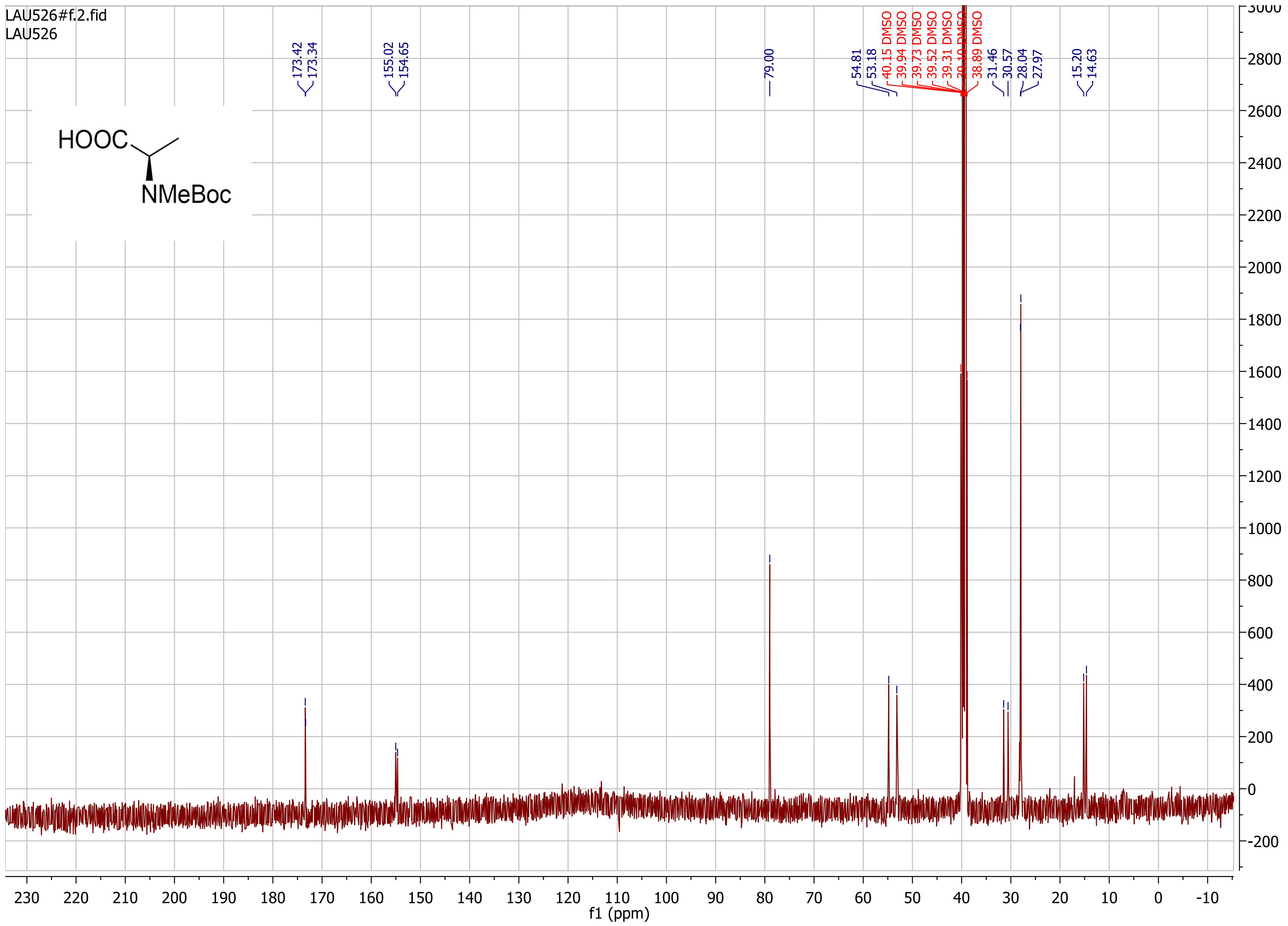

LAU526#f.2.fid
LAU526
173.42
173.34
155.02
154.65
79.00
54.81
53.18
40.15 DMSO
39.94 DMSO
39.73 DMSO
39.52 DMSO
39.31 DMSO
39.10 DMSO
38.89 DMSO
31.46
30.57
28.04
27.97
15.20
14.63
HOOC
NMeBoc
f1 (ppm)

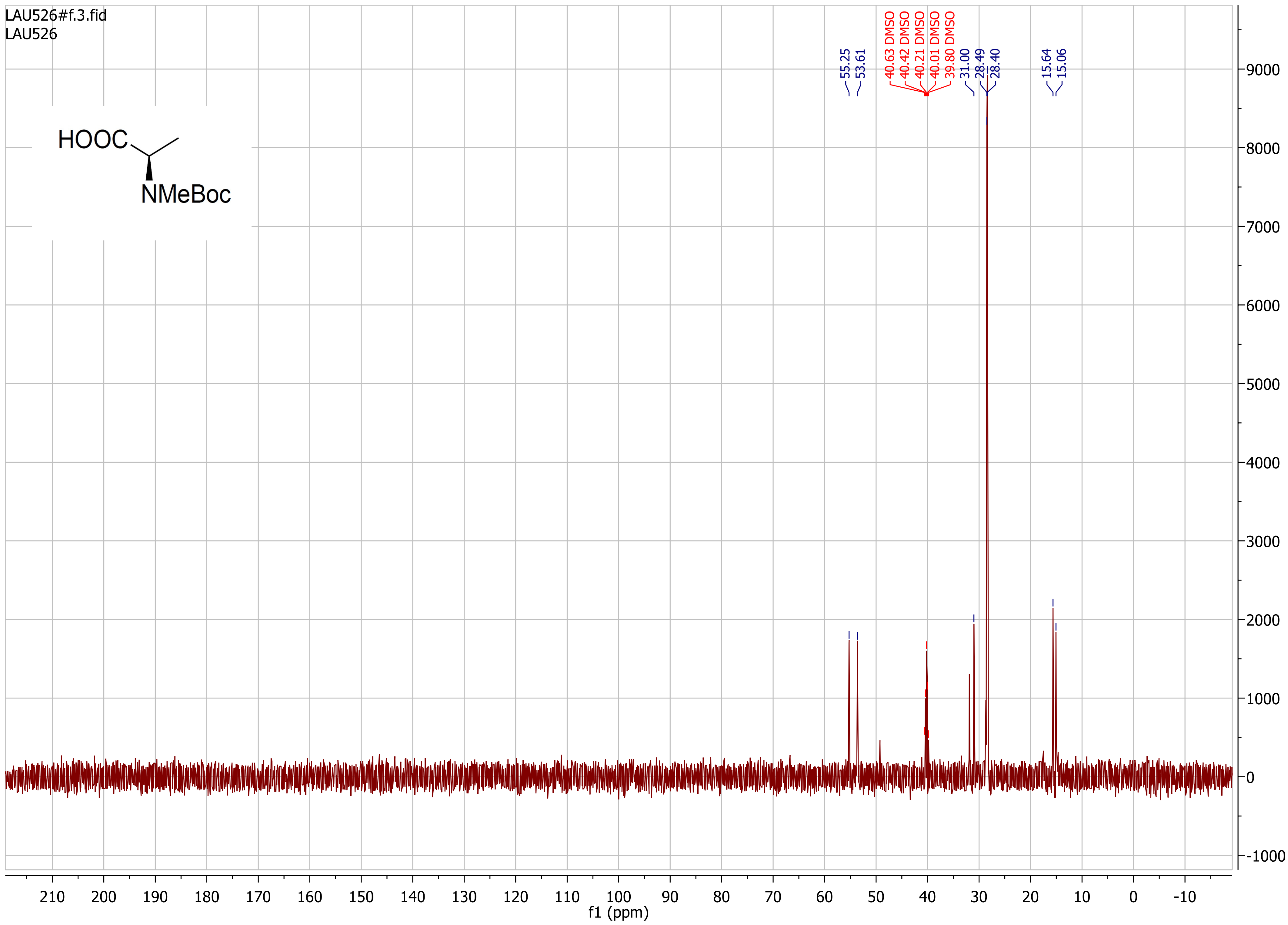

LAU526#f.3.fid
LAU526
HOOC
NMeBoc
55.25
53.61
40.63 DMSO
40.42 DMSO
40.21 DMSO
40.01 DMSO
39.80 DMSO
31.00
28.49
28.40
15.64
15.06
9000
8000
7000
6000
5000
4000
3000
2000
1000
0
-1000
210 200 190 180 170 160 150 140 130 120 110 100 90 80 70 60 50 40 30 20 10 0 -10
f1 (ppm)

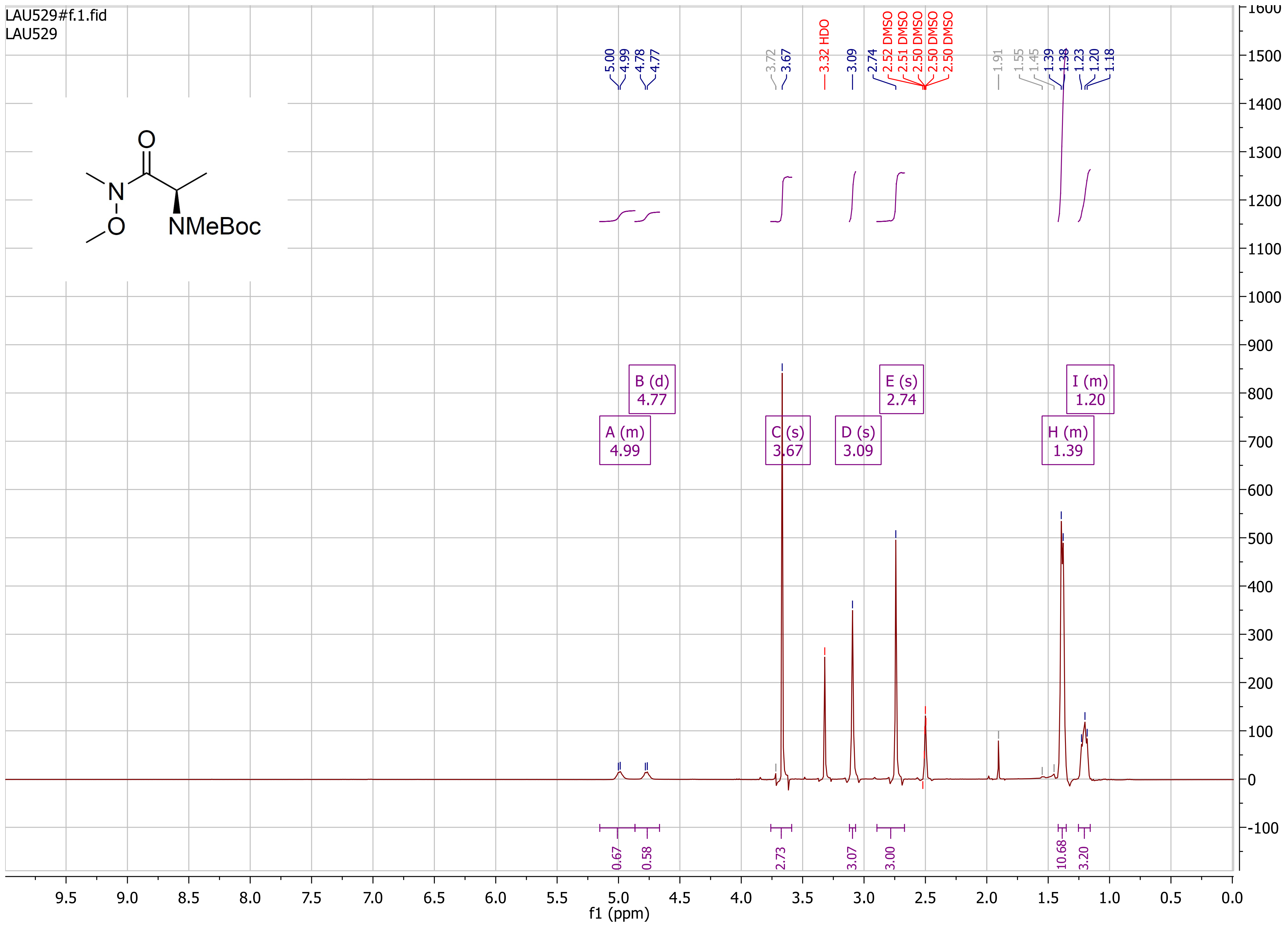
LAU529#f.1.fid
LAU529
NMeBoc
5.00
4.99
4.78
4.77
3.72
3.67
3.32 HDO
3.09
2.74
2.52 DMSO
2.51 DMSO
2.50 DMSO
2.50 DMSO
2.50 DMSO
1.91
1.55
1.45
1.39
1.38
1.23
1.20
1.18
B (d)
4.77
A (m)
4.99
C (s)
3.67
D (s)
3.09
E (s)
2.74
I (m)
1.20
H (m)
1.39
0.67
0.58
2.73
3.07
3.00
10.68
3.20
f1 (ppm)

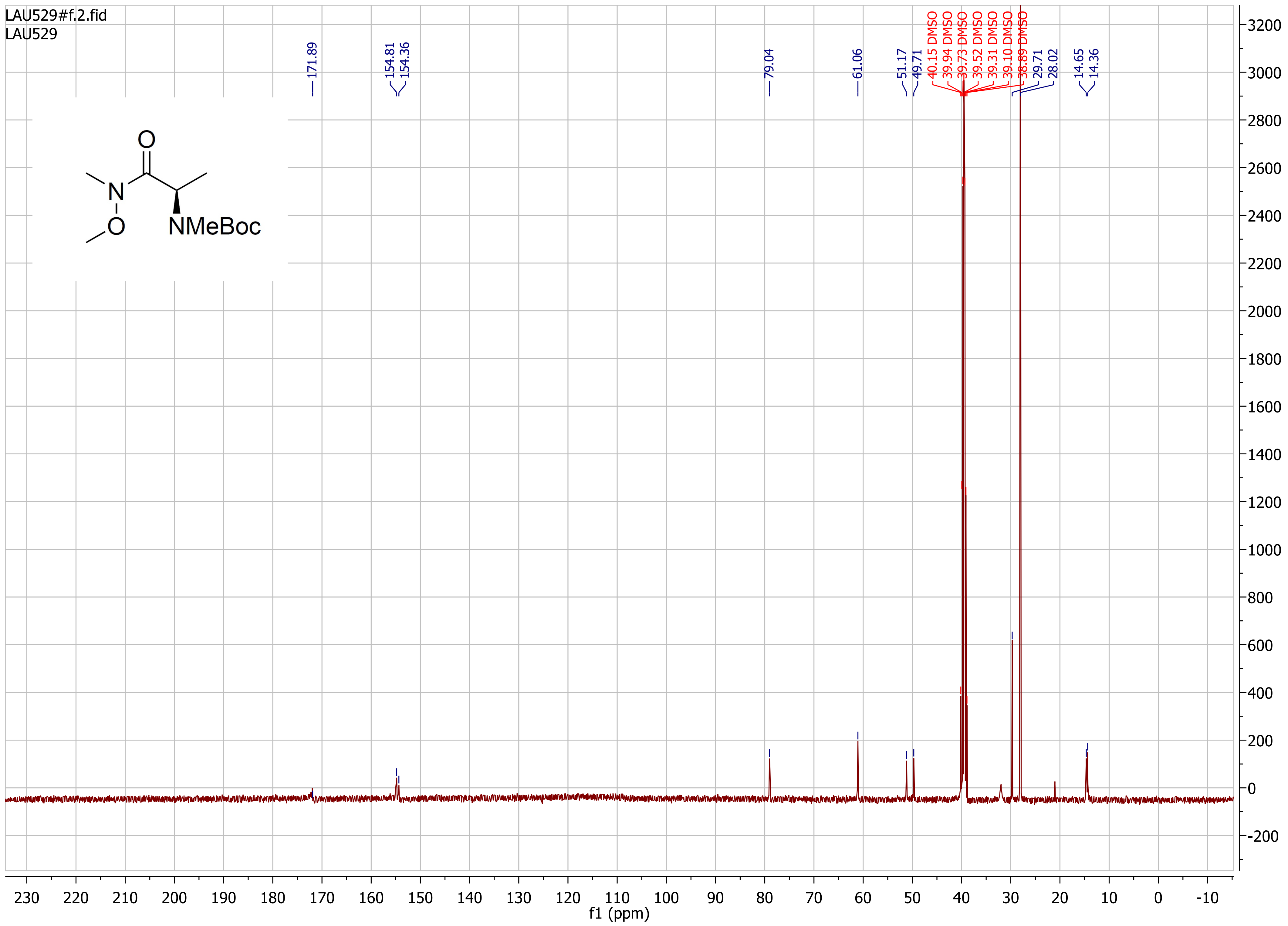
LAU529#f.2.fid
LAU529
171.89
154.81
154.36
79.04
61.06
51.17
49.71
40.15 DMSO
39.94 DMSO
39.73 DMSO
39.52 DMSO
39.31 DMSO
39.10 DMSO
38.89 DMSO
29.71
28.02
14.65
14.36
NMeBoc
f1 (ppm)

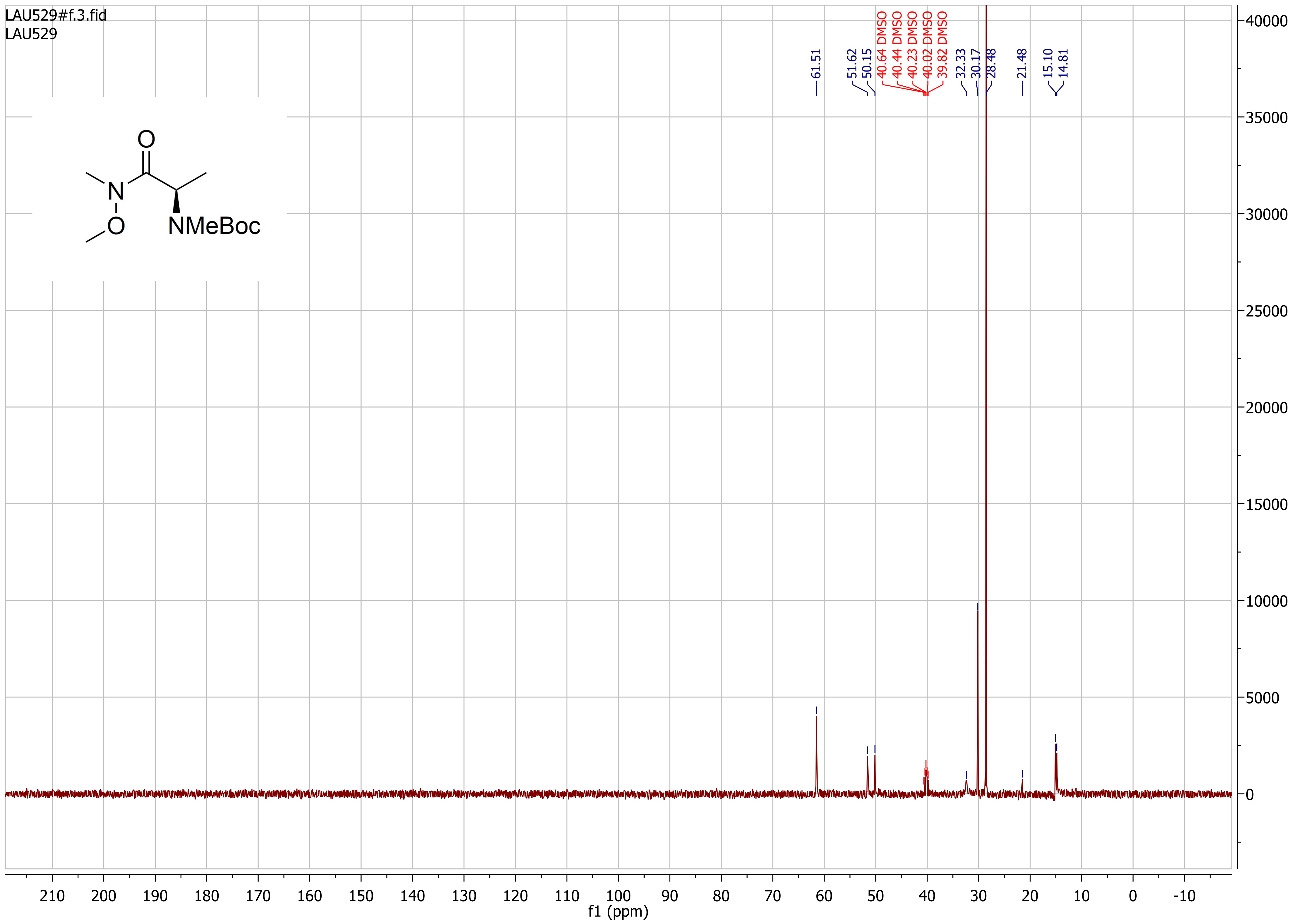
LAU529#f.3.fid
LAU529
NMeBoc
61.51
51.62
50.15
40.64 DMSO
40.44 DMSO
40.23 DMSO
40.02 DMSO
39.82 DMSO
32.33
30.17
28.48
21.48
15.10
14.81
f1 (ppm)

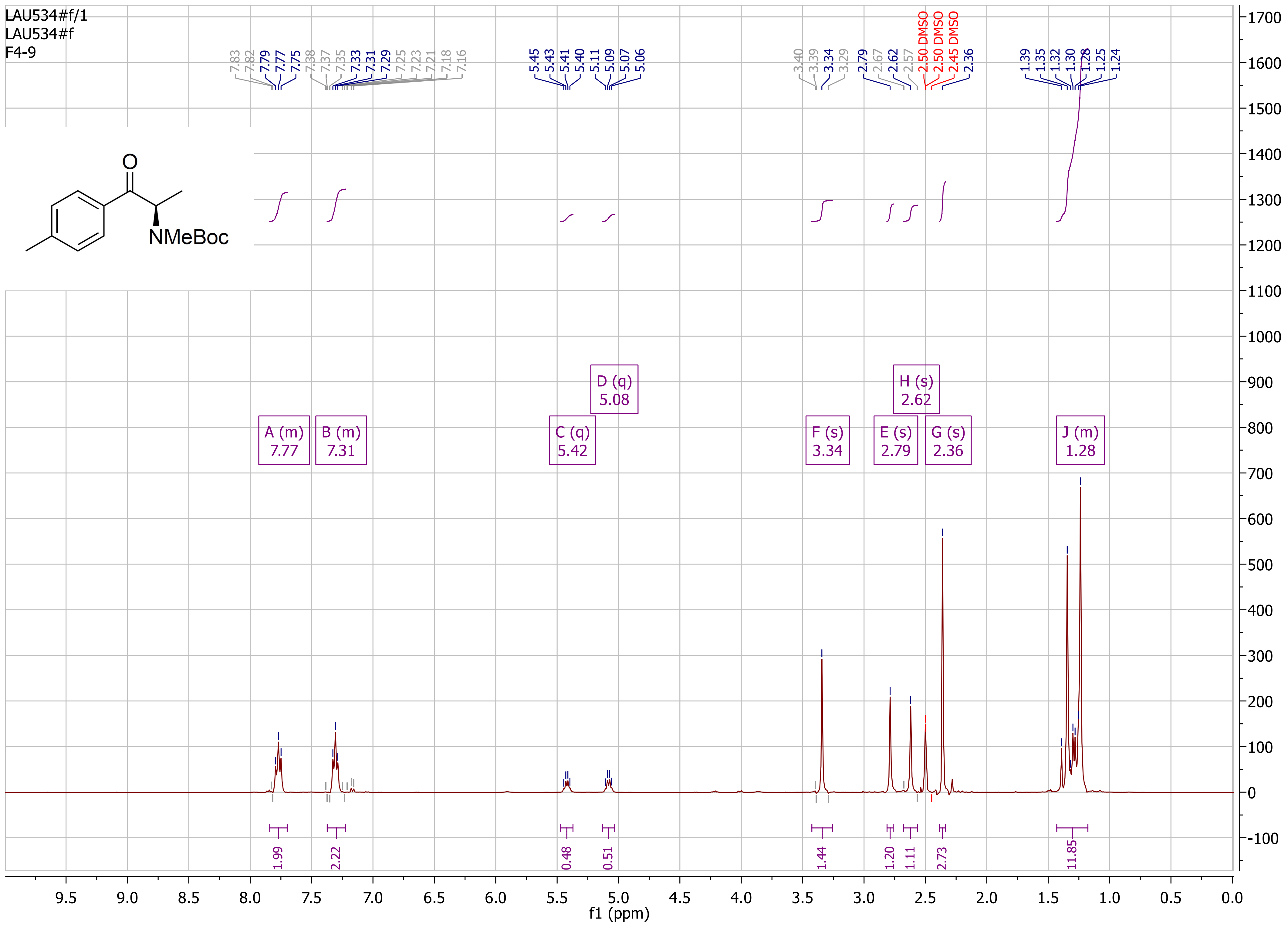

LAU534#f/1
LAU534#f
F4-9
NMeBoc
O
7.83
7.82
7.79
7.77
7.75
7.38
7.37
7.35
7.33
7.31
7.29
7.25
7.23
7.21
7.18
7.16
5.45
5.43
5.41
5.40
5.11
5.09
5.07
5.06
3.40
3.39
3.34
3.29
2.79
2.67
2.62
2.57
2.50 DMSO
2.50 DMSO
2.45 DMSO
2.36
1.39
1.35
1.32
1.30
1.28
1.25
1.24
A (m) 7.77
B (m) 7.31
C (q) 5.42
D (q) 5.08
F (s) 3.34
E (s) 2.79
H (s) 2.62
G (s) 2.36
J (m) 1.28
1.99
2.22
0.48
0.51
1.44
1.20
1.11
2.73
11.85
f1 (ppm)

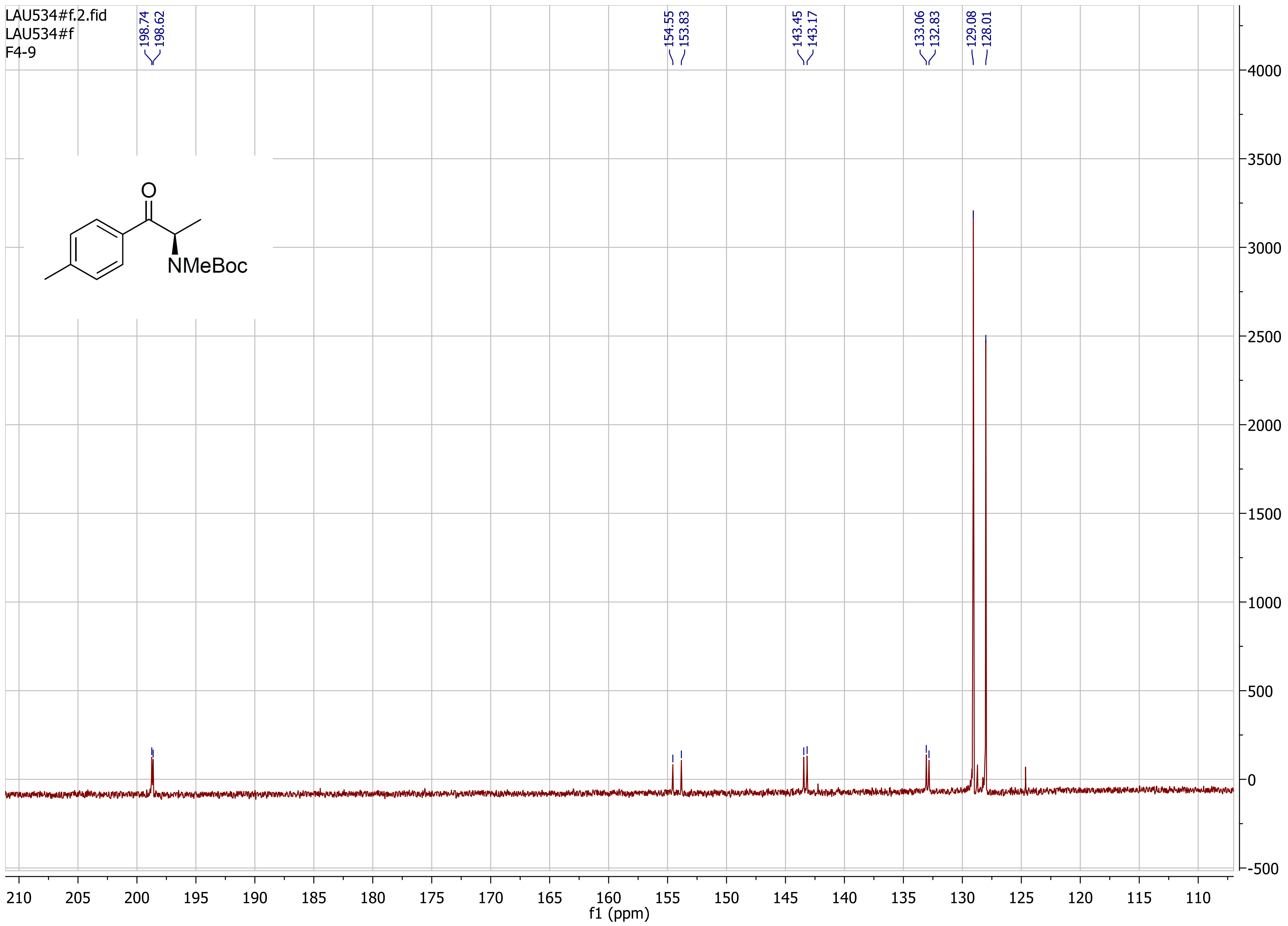
LAU534#f.2.fid
LAU534#f
F4-9
198.74
198.62
154.55
153.83
143.45
143.17
133.06
132.83
129.08
128.01
O
NMeBoc
4000
3500
3000
2500
2000
1500
1000
500
0
-500
210
205
200
195
190
185
180
175
170
165
160
155
150
145
140
135
130
125
120
115
110
f1 (ppm)

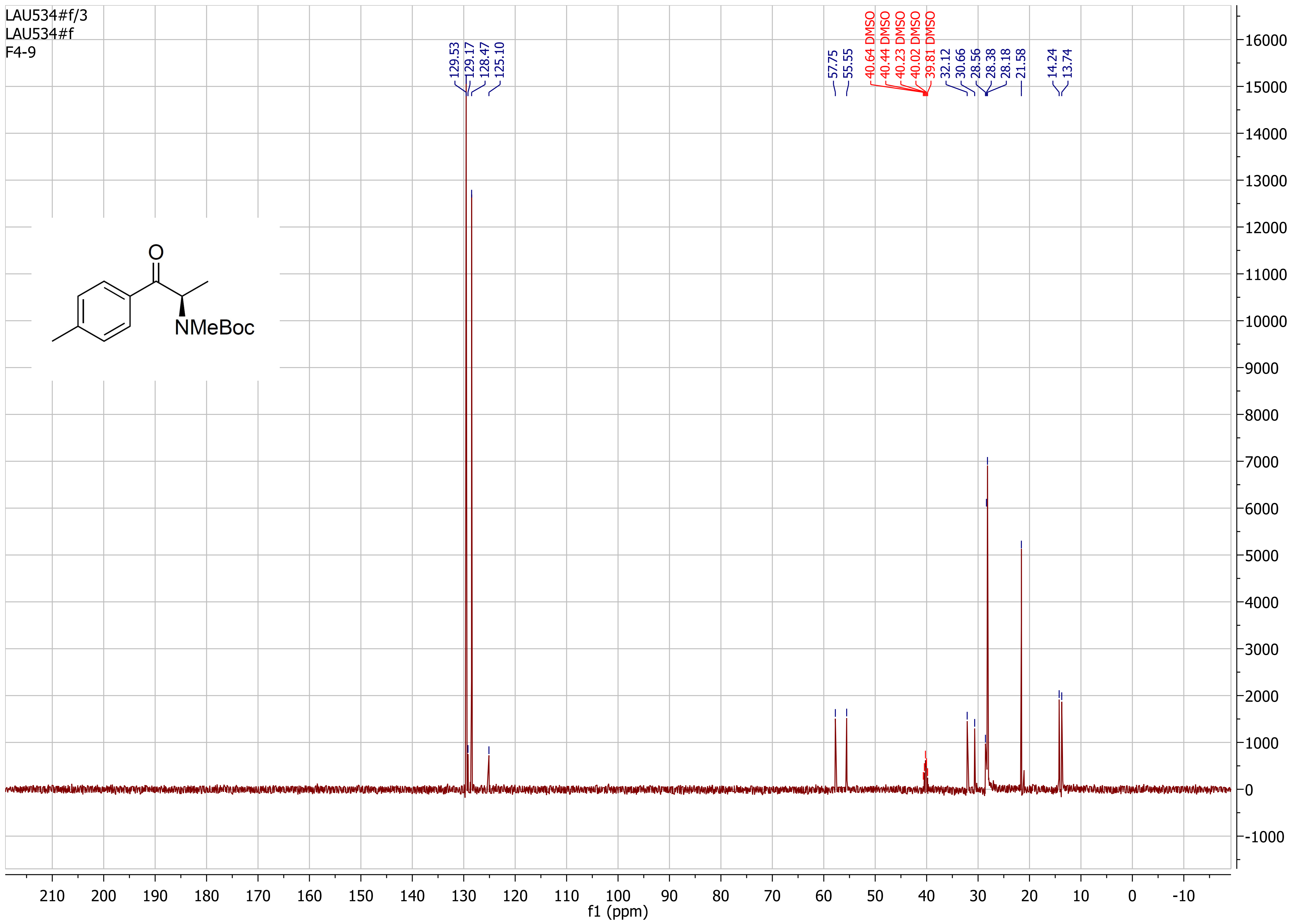

LAU534#f/3
LAU534#f
F4-9
129.53
129.17
128.47
125.10
57.75
55.55
40.64 DMSO
40.44 DMSO
40.23 DMSO
40.02 DMSO
39.81 DMSO
32.12
30.66
28.56
28.38
28.18
21.58
14.24
13.74
O
NMeBoc
f1 (ppm)

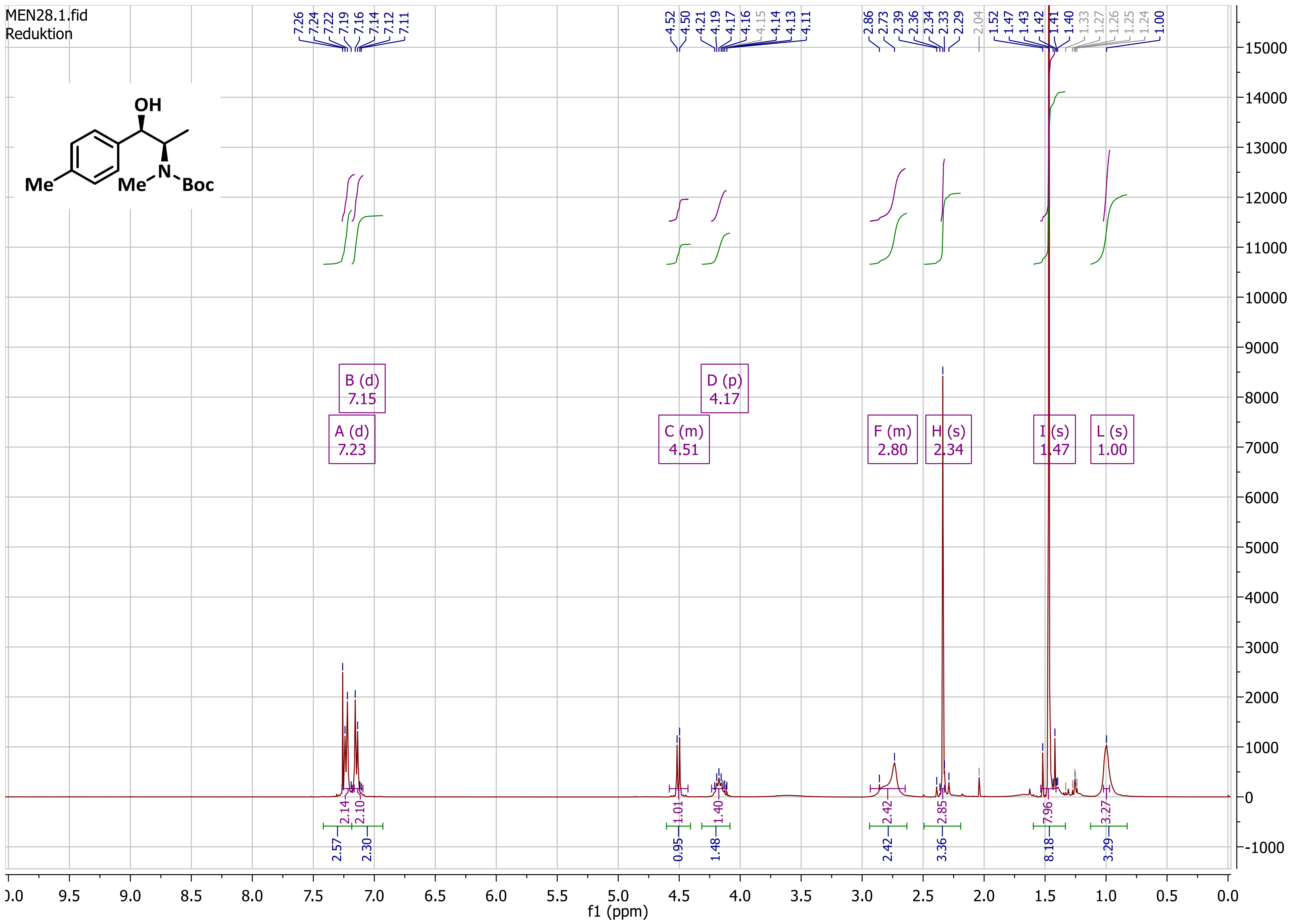

MEN28.1.fid
Reduktion
OH
Me
Me
N
Boc
7.26 7.24 7.22 7.19 7.16 7.14 7.12 7.11
4.52 4.50 4.21 4.19 4.17 4.16 4.15 4.14 4.13 4.11
2.86 2.73 2.39 2.36 2.34 2.33 2.29 2.04
1.52 1.47 1.43 1.42 1.41 1.40 1.33 1.27 1.26 1.25 1.24 1.00
B (d) 7.15
A (d) 7.23
C (m) 4.51
D (p) 4.17
F (m) 2.80
H (s) 2.34
I (s) 1.47
L (s) 1.00
2.14 2.10 2.57 2.30
1.01 0.95 1.40 1.48
2.42 2.42 2.85 3.36
7.96 8.18 3.27 3.29
f1 (ppm)

MEN28.2.fid
Reduktion

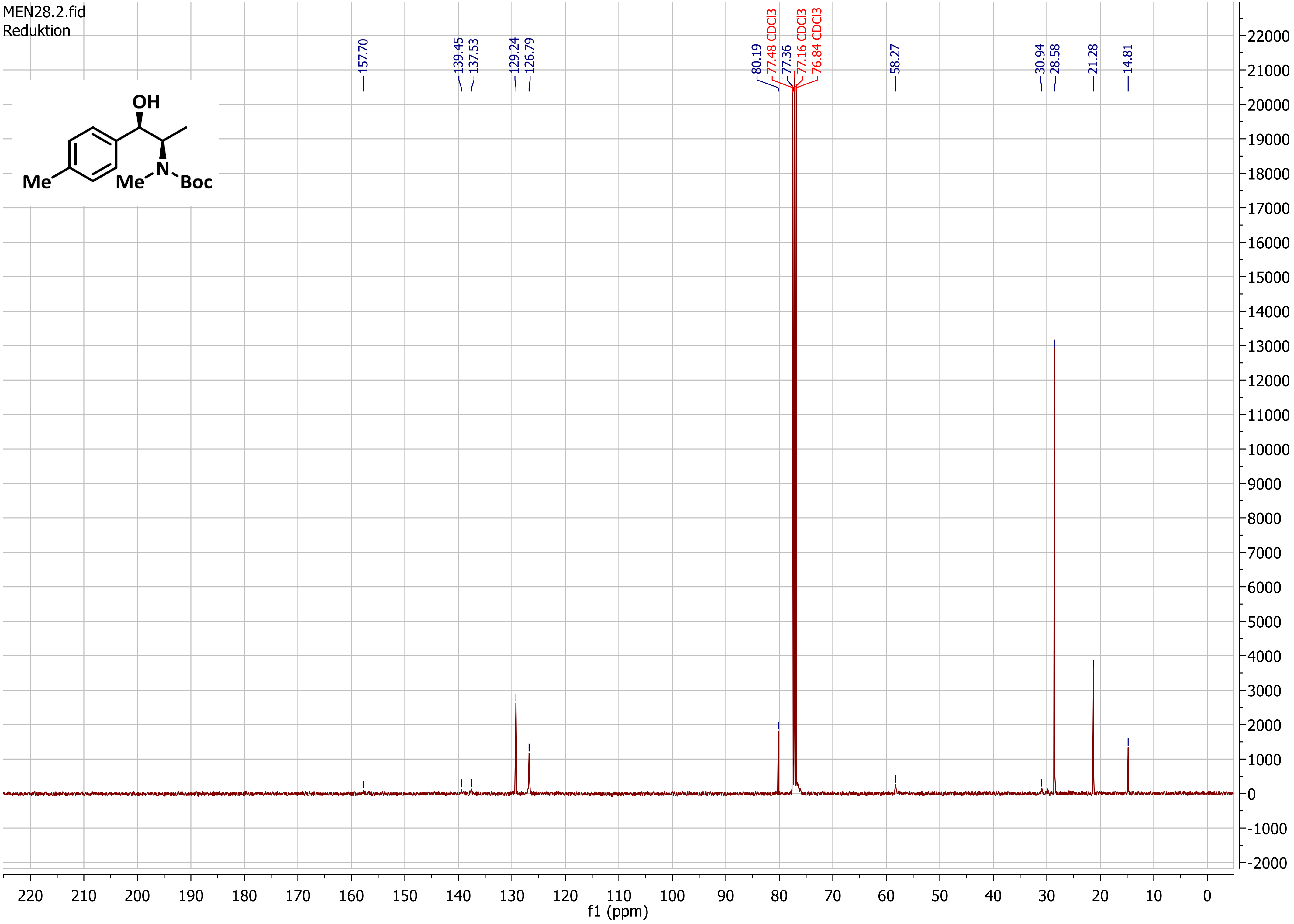

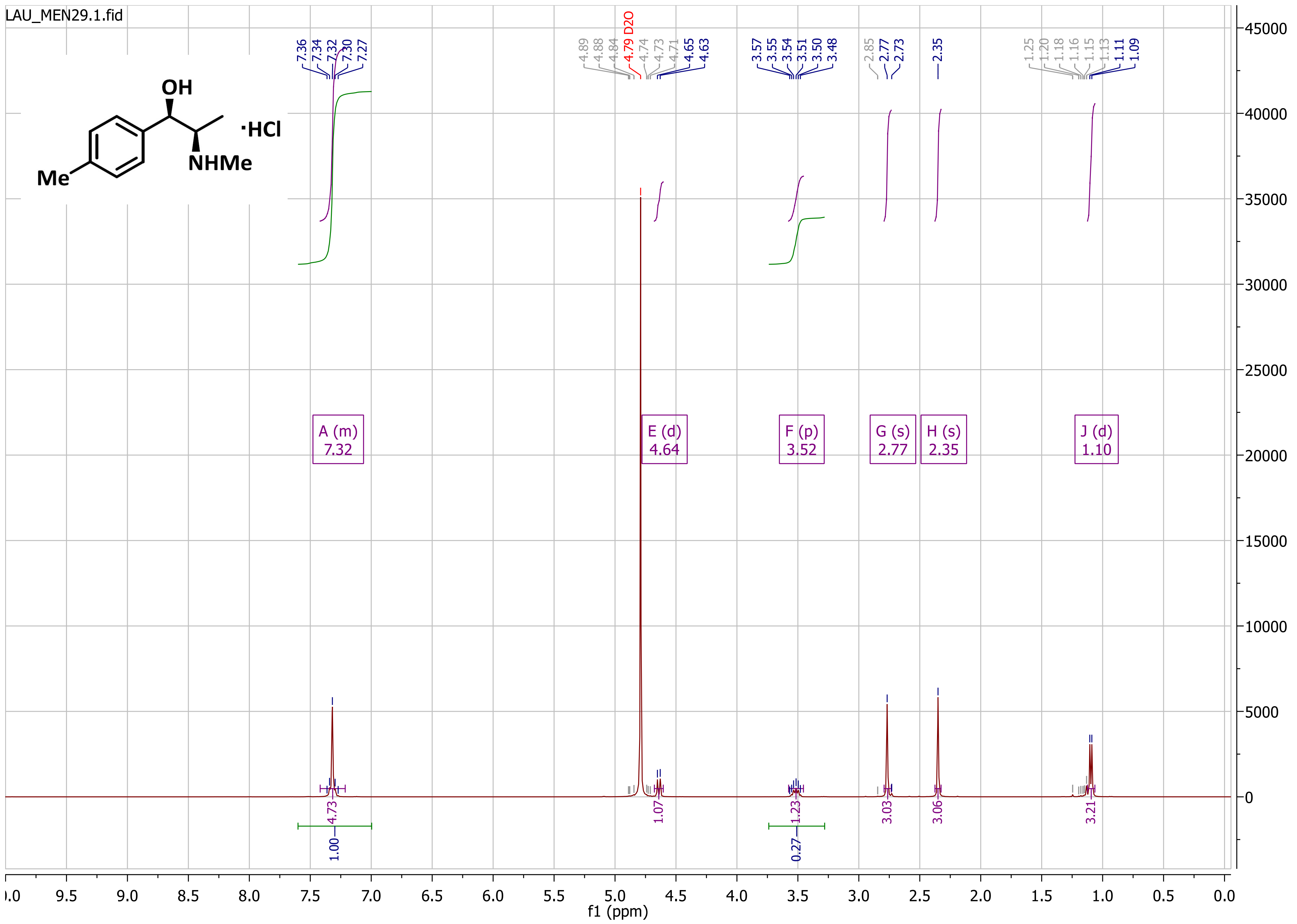
LAU_MEN29.1.fid
OH
·HCl
NHMe
Me
7.36
7.34
7.32
7.30
7.27
4.89
4.88
4.84
4.79 D2O
4.74
4.73
4.71
4.65
4.63
3.57
3.55
3.54
3.51
3.50
3.48
2.85
2.77
2.73
2.35
1.25
1.20
1.18
1.16
1.15
1.13
1.11
1.09
A (m) 7.32
E (d) 4.64
F (p) 3.52
G (s) 2.77
H (s) 2.35
J (d) 1.10
4.73
1.00
1.07
1.23
0.27
3.03
3.06
3.21
f1 (ppm)

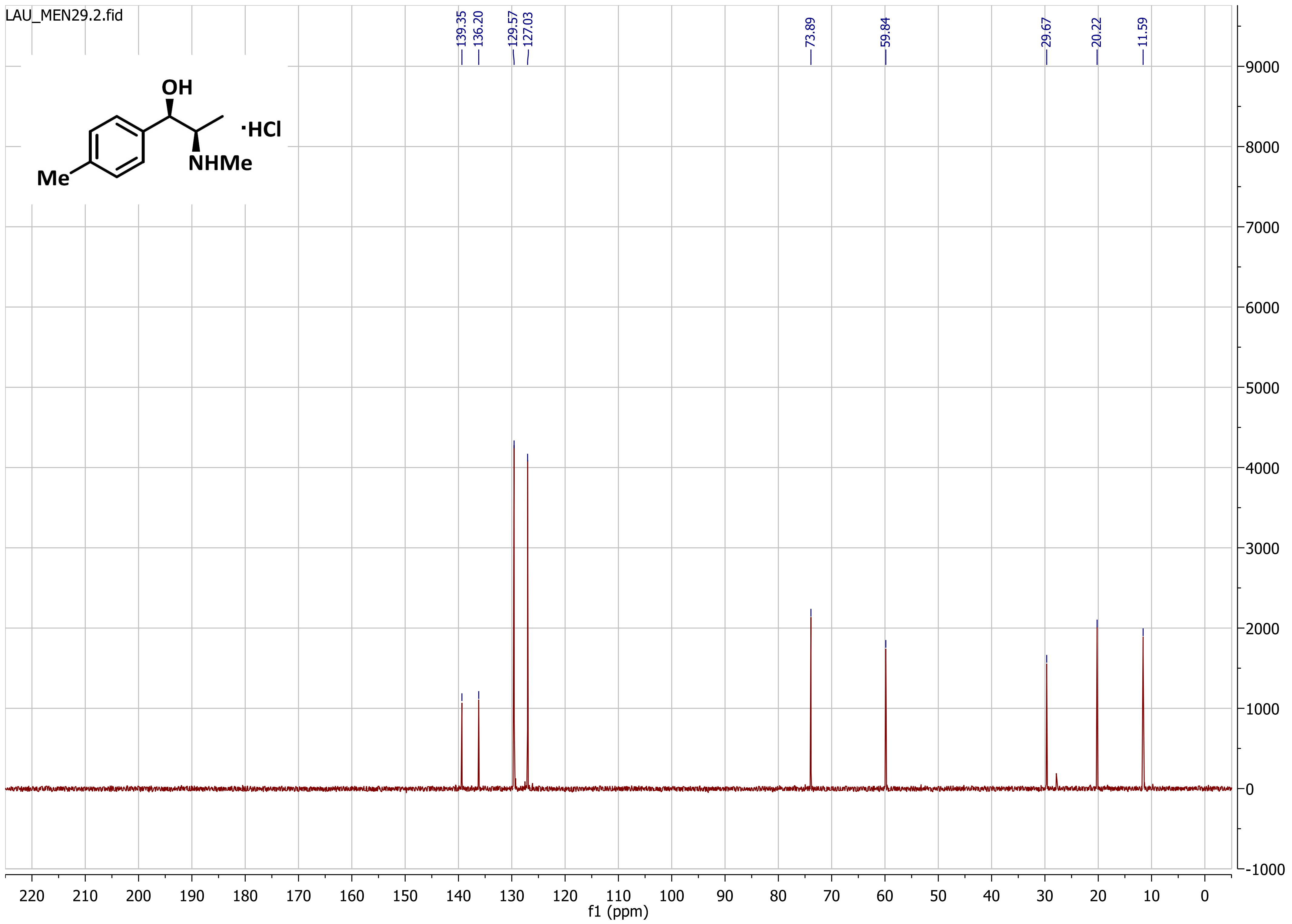
LAU_MEN29.2.fid
139.35
136.20
129.57
127.03
73.89
59.84
29.67
20.22
11.59
OH
·HCl
NHMe
Me
9000
8000
7000
6000
5000
4000
3000
2000
1000
0
-1000
220
210
200
190
180
170
160
150
140
130
120
110
100
90
80
70
60
50
40
30
20
10
0
f1 (ppm)

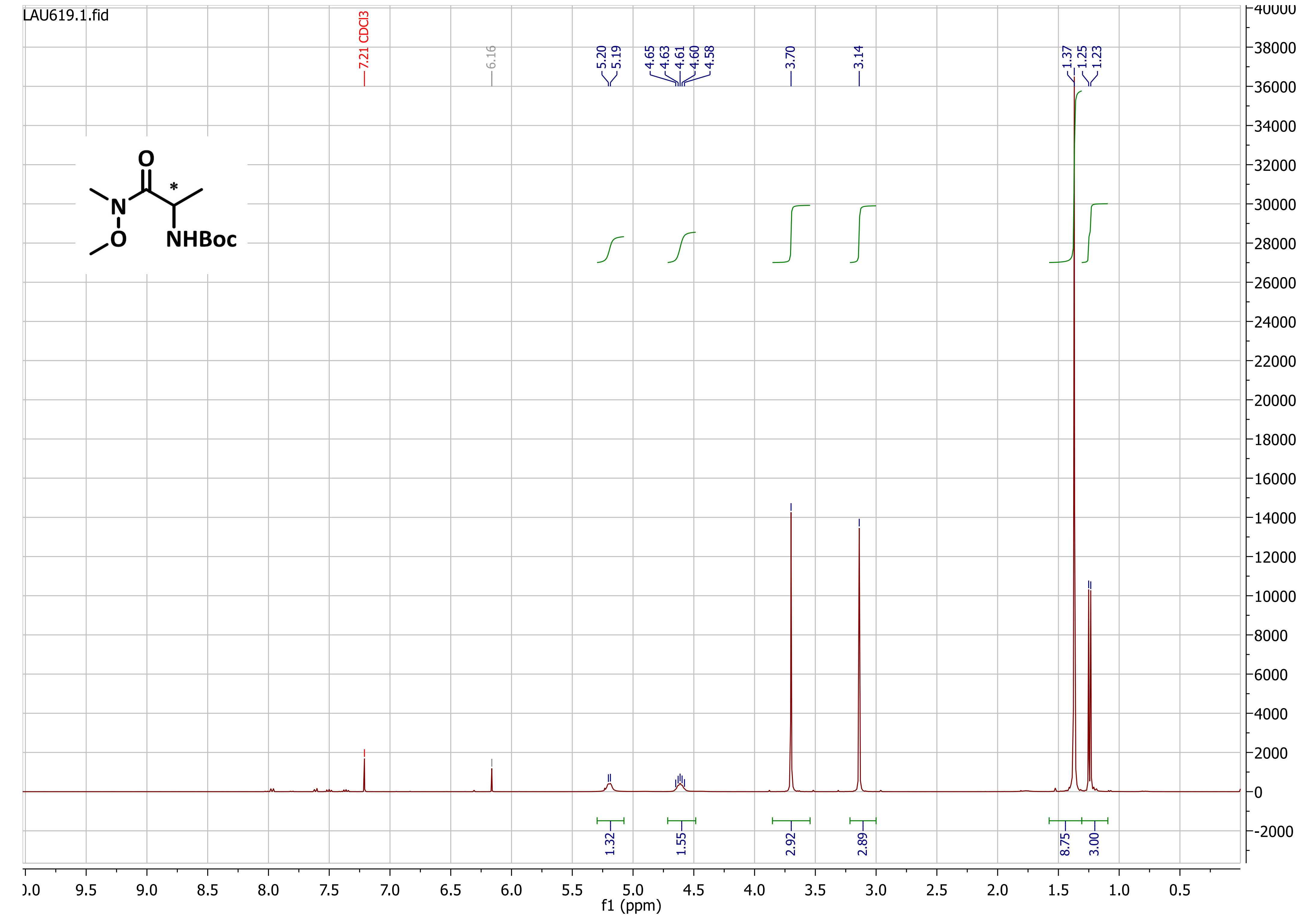
LAU619.1.fid
7.21 CDCl3
6.16
5.20
5.19
4.65
4.63
4.61
4.60
4.58
3.70
3.14
1.37
1.25
1.23
O
N
O
NHBoc
*
1.32
1.55
2.92
2.89
8.75
3.00
f1 (ppm)

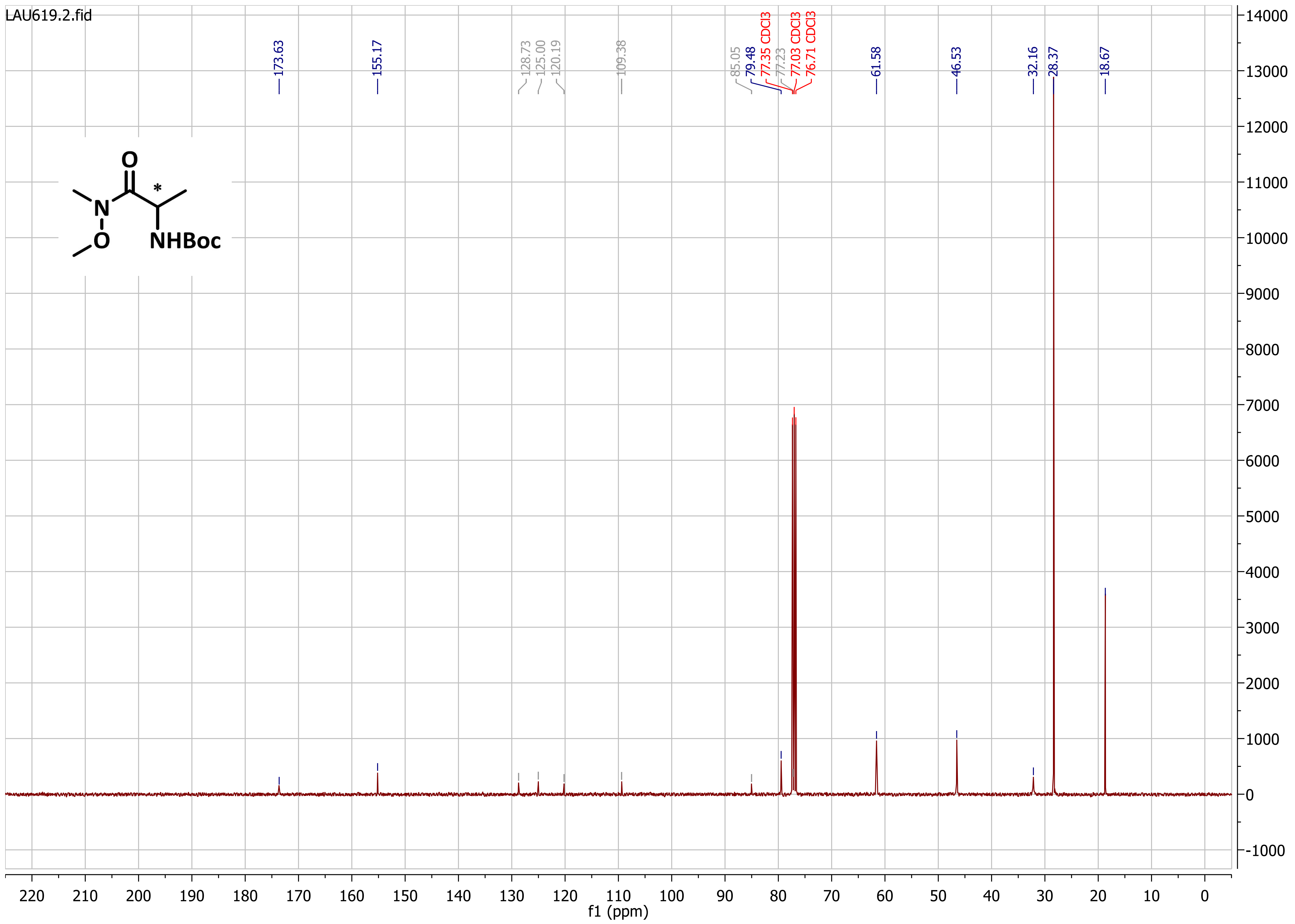
LAU619.2.fid
173.63
155.17
128.73
125.00
120.19
109.38
85.05
79.48
77.35 CDCl3
77.23
77.03 CDCl3
76.71 CDCl3
61.58
46.53
32.16
28.37
18.67
O
N
O
*
NHBoc
f1 (ppm)

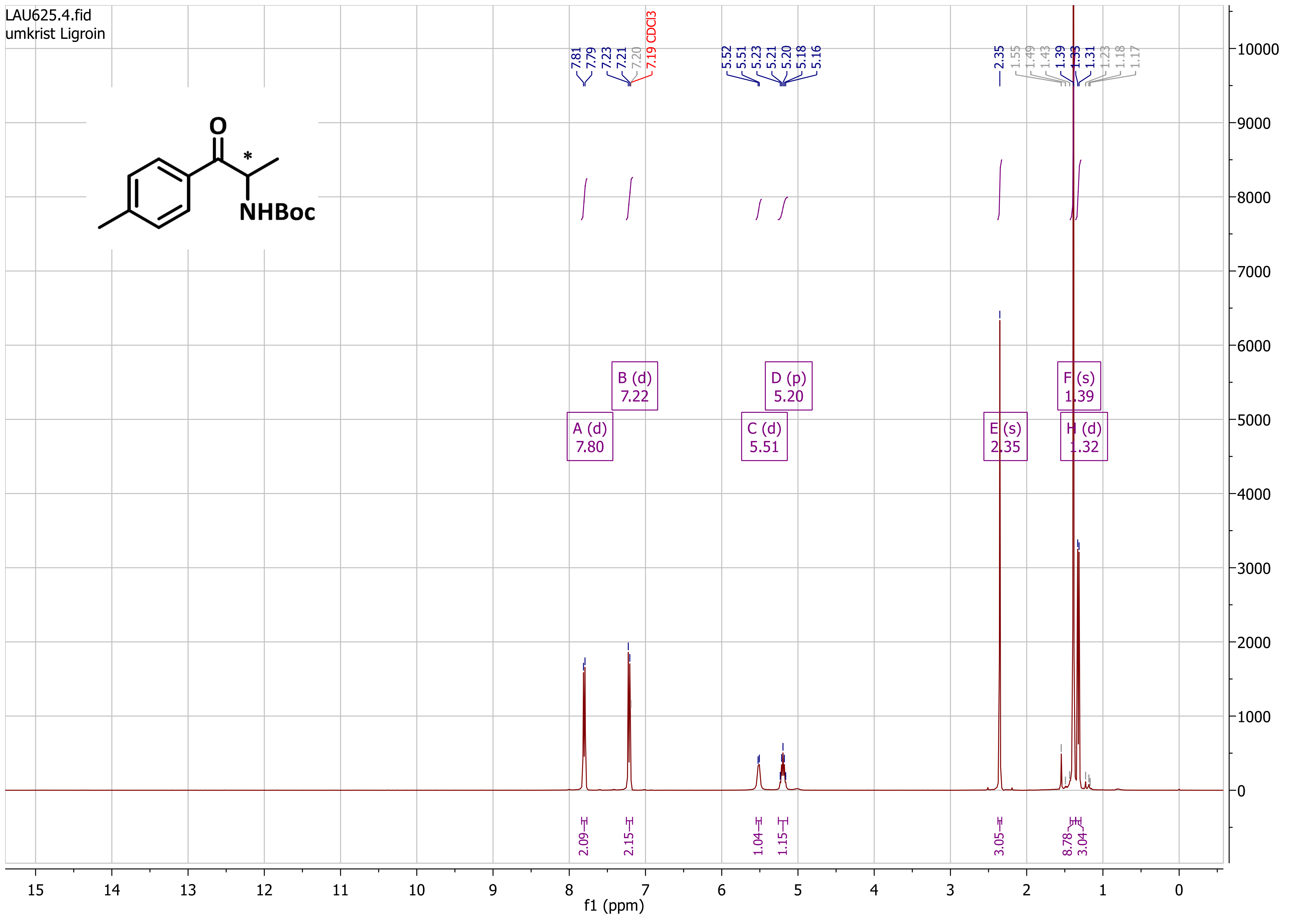

LAU625.4.fid
umkrist Ligroin
NHBoc
O
*
7.81
7.79
7.23
7.21
7.20
7.19 CDCl3
5.52
5.51
5.23
5.21
5.20
5.18
5.16
2.35
1.55
1.49
1.43
1.39
1.33
1.31
1.23
1.18
1.17
A (d) 7.80
B (d) 7.22
C (d) 5.51
D (p) 5.20
E (s) 2.35
F (s) 1.39
H (d) 1.32
2.09
2.15
1.04
1.15
3.05
8.78
3.04
f1 (ppm)

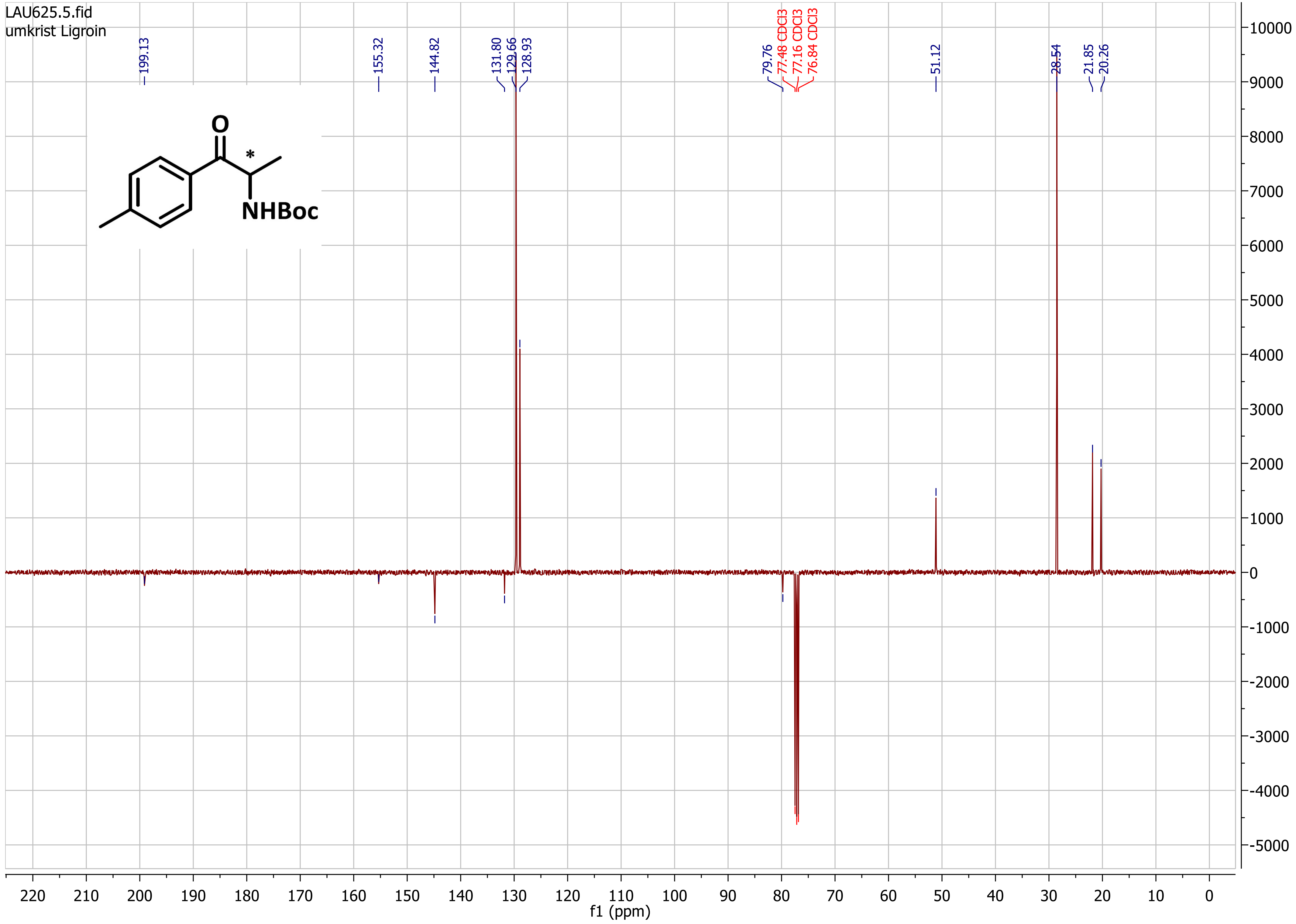
LAU625.5.fid
umkrist Ligroin
199.13
155.32
144.82
131.80
129.66
128.93
79.76
77.48 CDCl3
77.16 CDCl3
76.84 CDCl3
51.12
28.54
21.85
20.26
O
*
NHBoc
f1 (ppm)

LAU580.1.fid

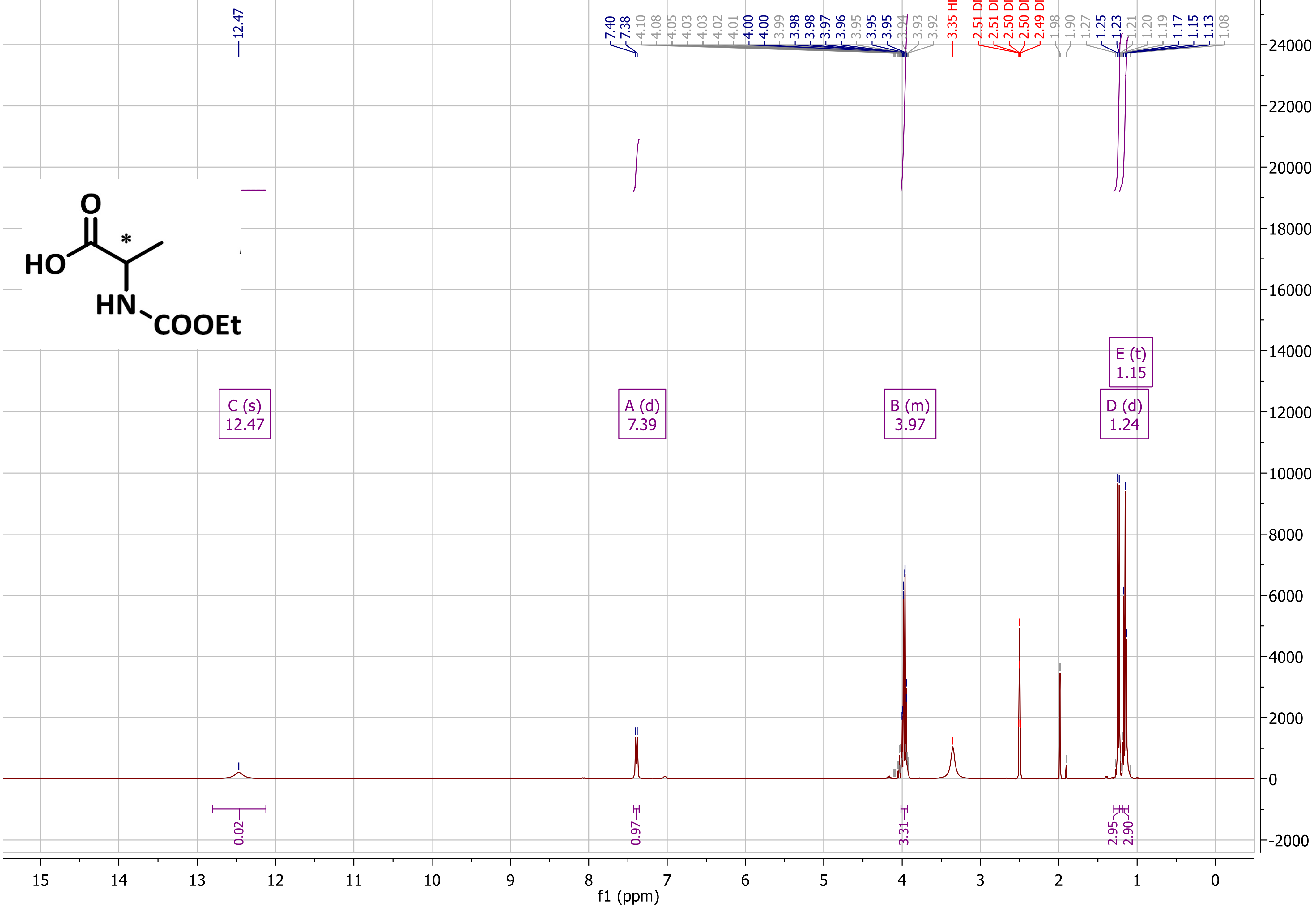

12.47
7.40
7.38
4.10
4.08
4.05
4.03
4.03
4.02
4.01
4.00
4.00
3.99
3.98
3.98
3.97
3.96
3.95
3.95
3.95
3.94
3.93
3.92
3.35 HDO
2.51 DMSO
2.51 DMSO
2.50 DMSO
2.50 DMSO
2.49 DMSO
1.98
1.90
1.27
1.25
1.23
1.21
1.20
1.19
1.17
1.15
1.13
1.08
HO
O
*
HN
COOEt
C (s)
12.47
A (d)
7.39
B (m)
3.97
D (d)
1.24
E (t)
1.15
0.02
0.97
3.31
2.95
2.90
f1 (ppm)

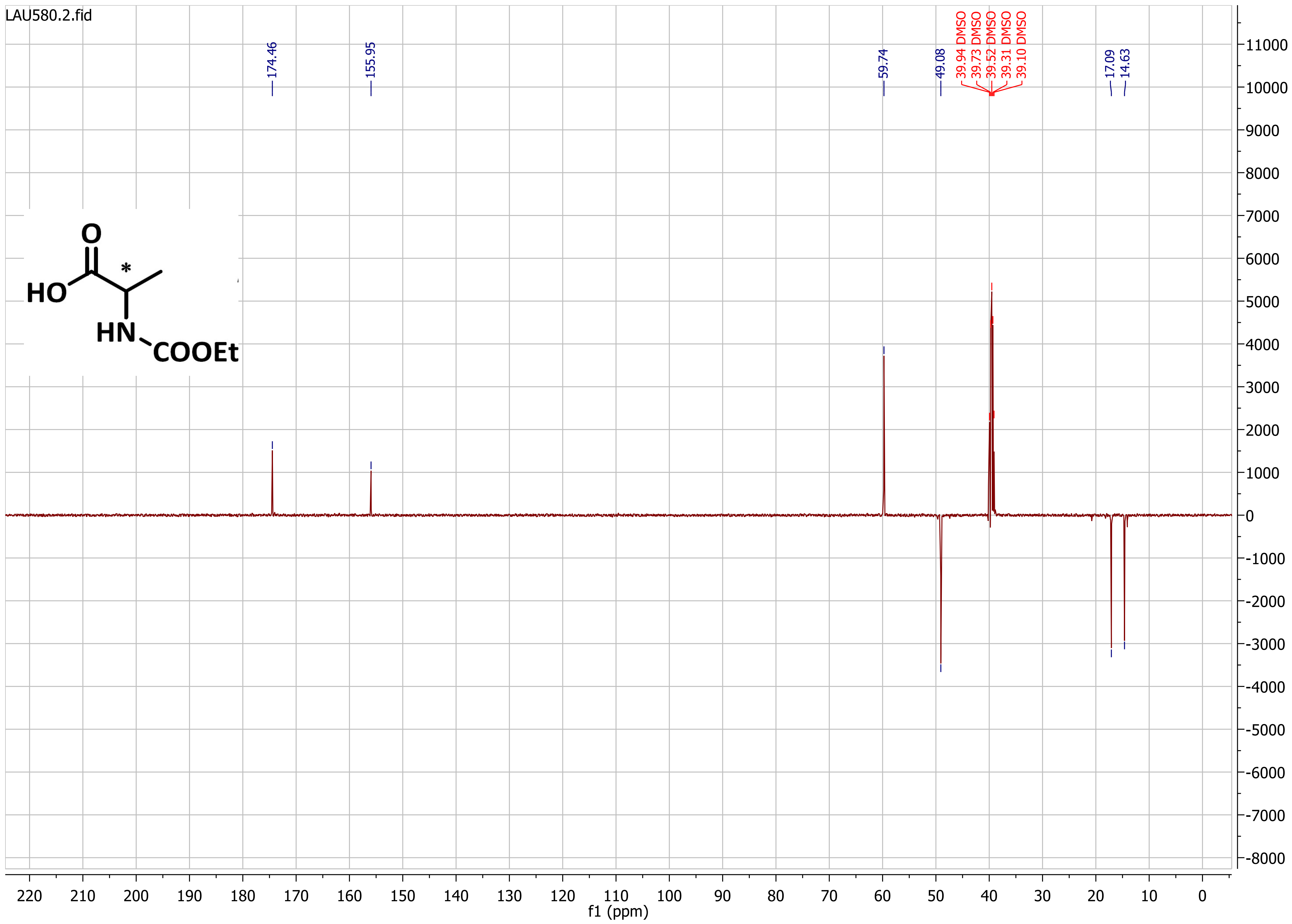
LAU580.2.fid
174.46
155.95
59.74
49.08
39.94 DMSO
39.73 DMSO
39.52 DMSO
39.31 DMSO
39.10 DMSO
17.09
14.63
HO
O
*
HN
COOEt
f1 (ppm)

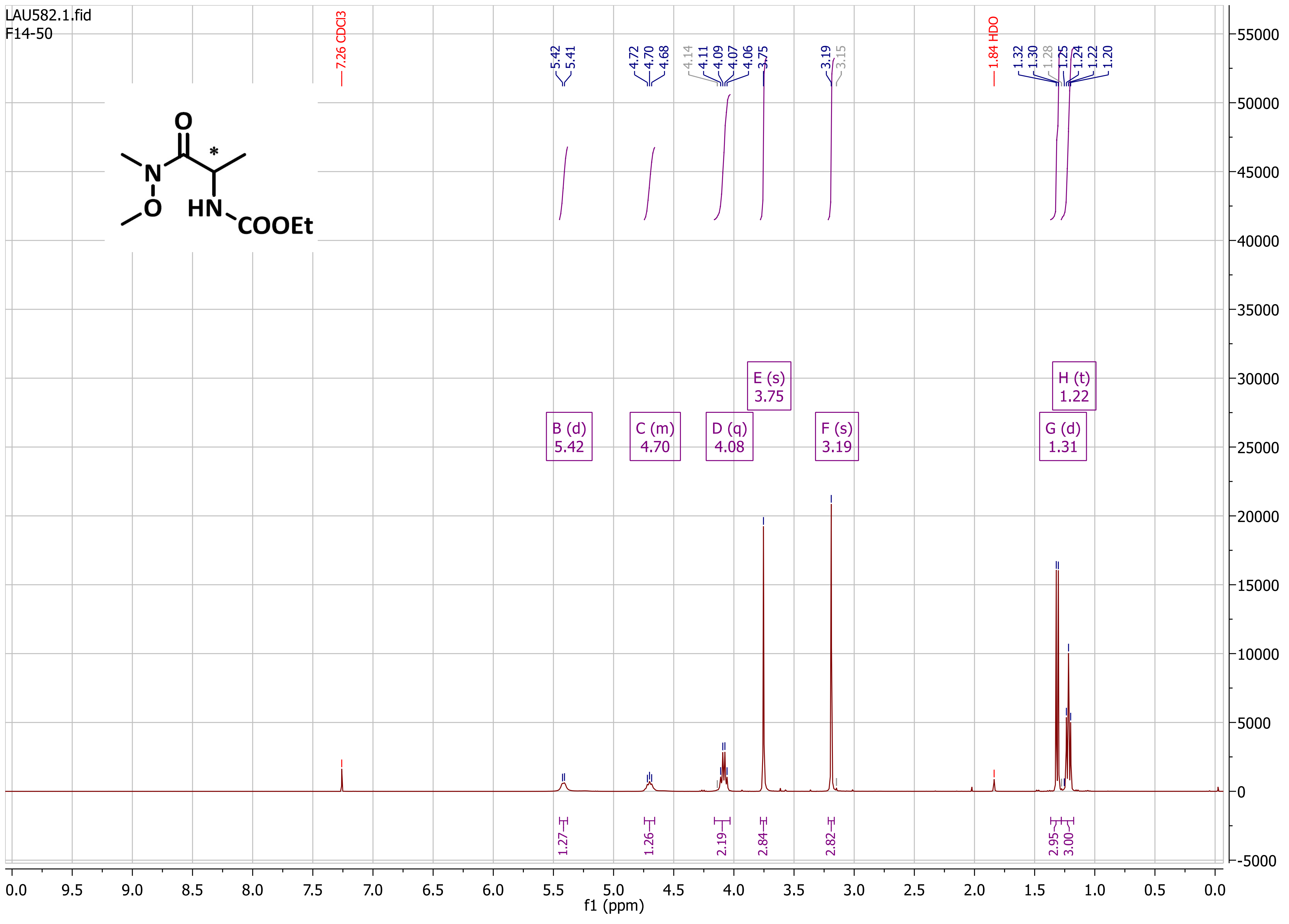

LAU582.1.fid
F14-50
7.26 CDCl3
5.42
5.41
4.72
4.70
4.68
4.14
4.11
4.09
4.07
4.06
3.75
3.19
3.15
1.84 HDO
1.32
1.30
1.28
1.25
1.24
1.22
1.20
O
N
O
*
HN
COOEt
B (d)
5.42
C (m)
4.70
D (q)
4.08
E (s)
3.75
F (s)
3.19
G (d)
1.31
H (t)
1.22
1.27
1.26
2.19
2.84
2.82
2.95
3.00
f1 (ppm)

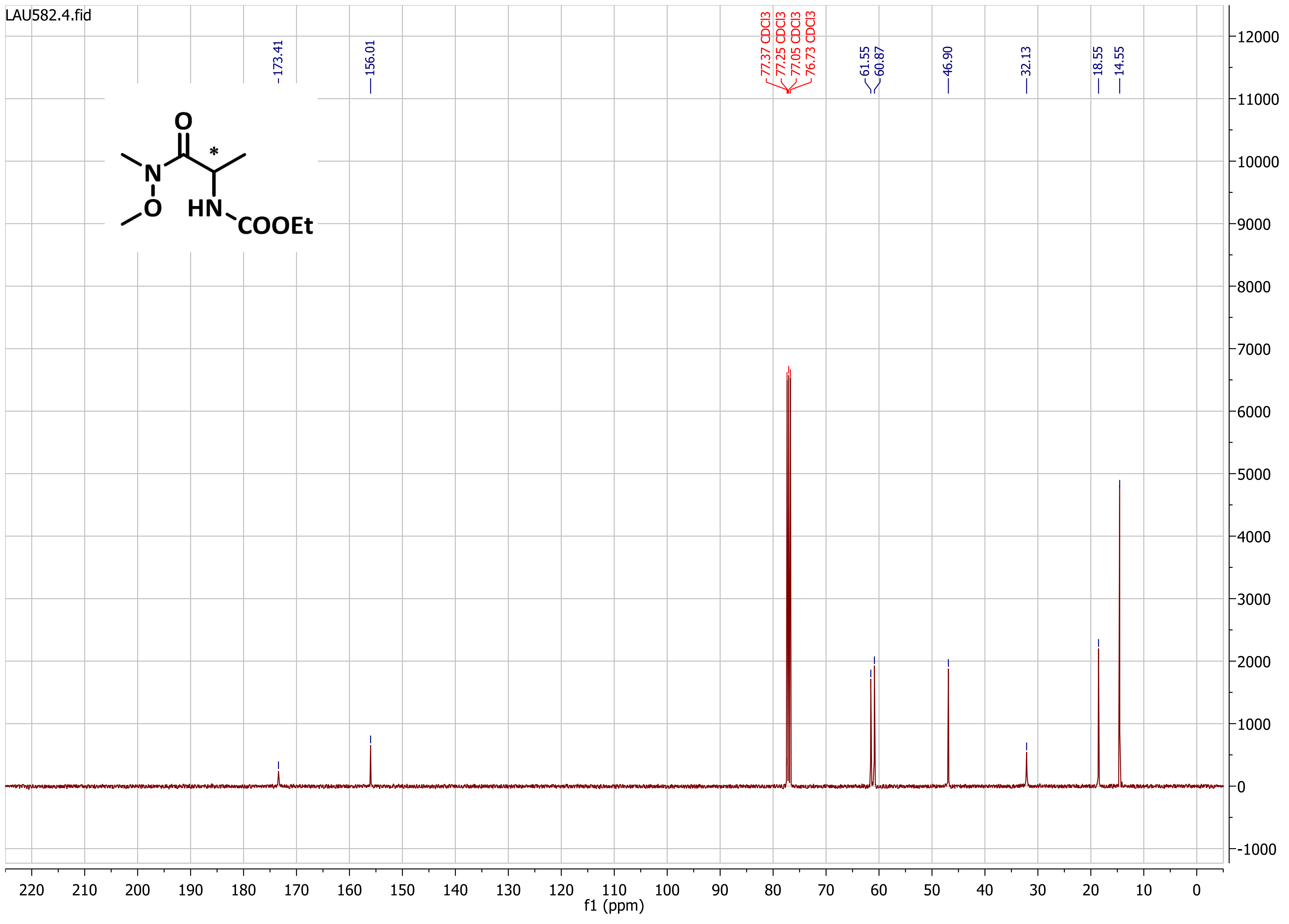
LAU582.4.fid
173.41
156.01
77.37 CDCl3
77.25 CDCl3
77.05 CDCl3
76.73 CDCl3
61.55
60.87
46.90
32.13
18.55
14.55
O
N
O
HN
COOEt
*
f1 (ppm)

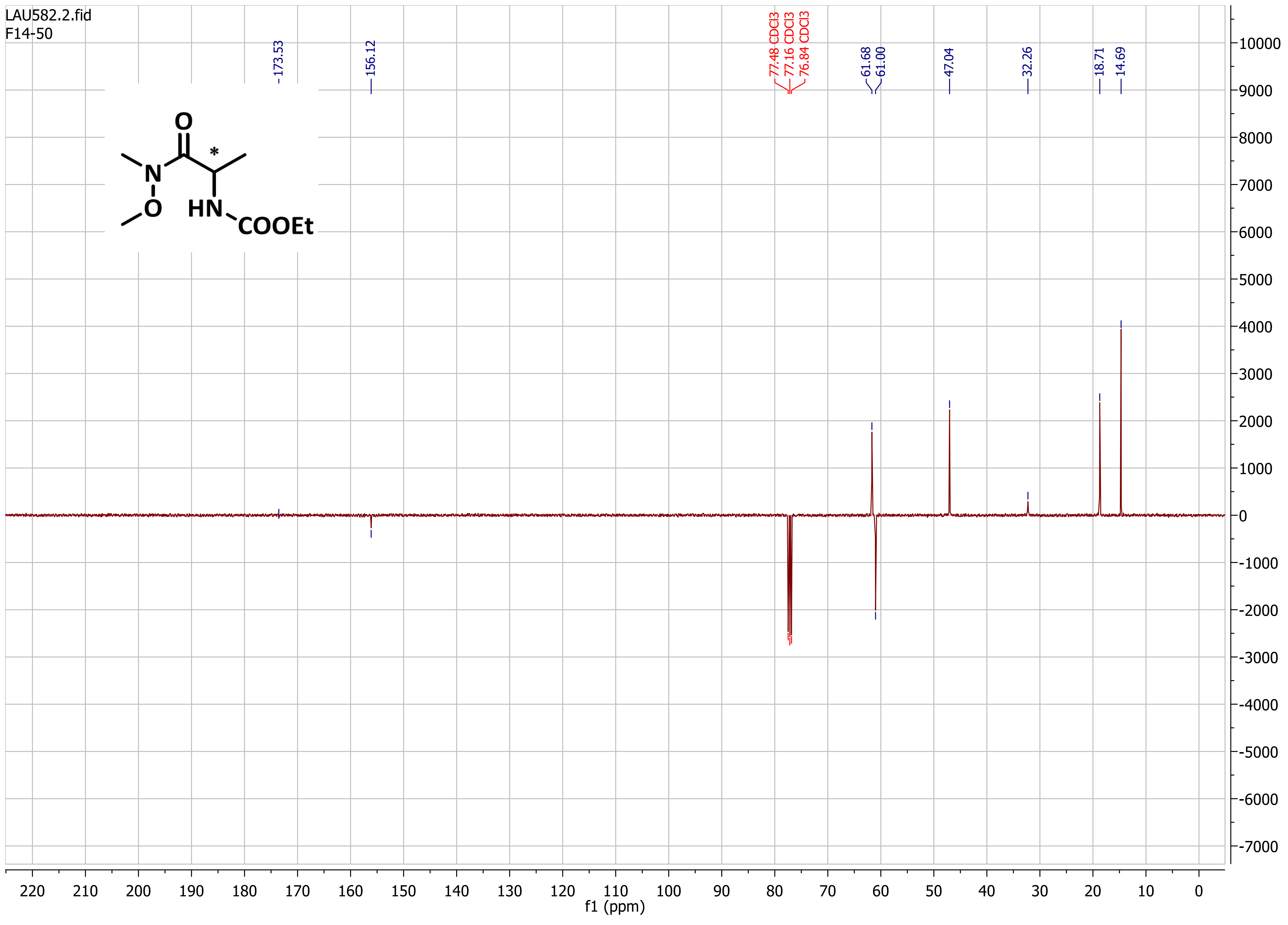

LAU582.2.fid
F14-50
173.53
156.12
77.48 CDCl3
77.16 CDCl3
76.84 CDCl3
61.68
61.00
47.04
32.26
18.71
14.69
O
N
O
HN
COOEt
*
f1 (ppm)

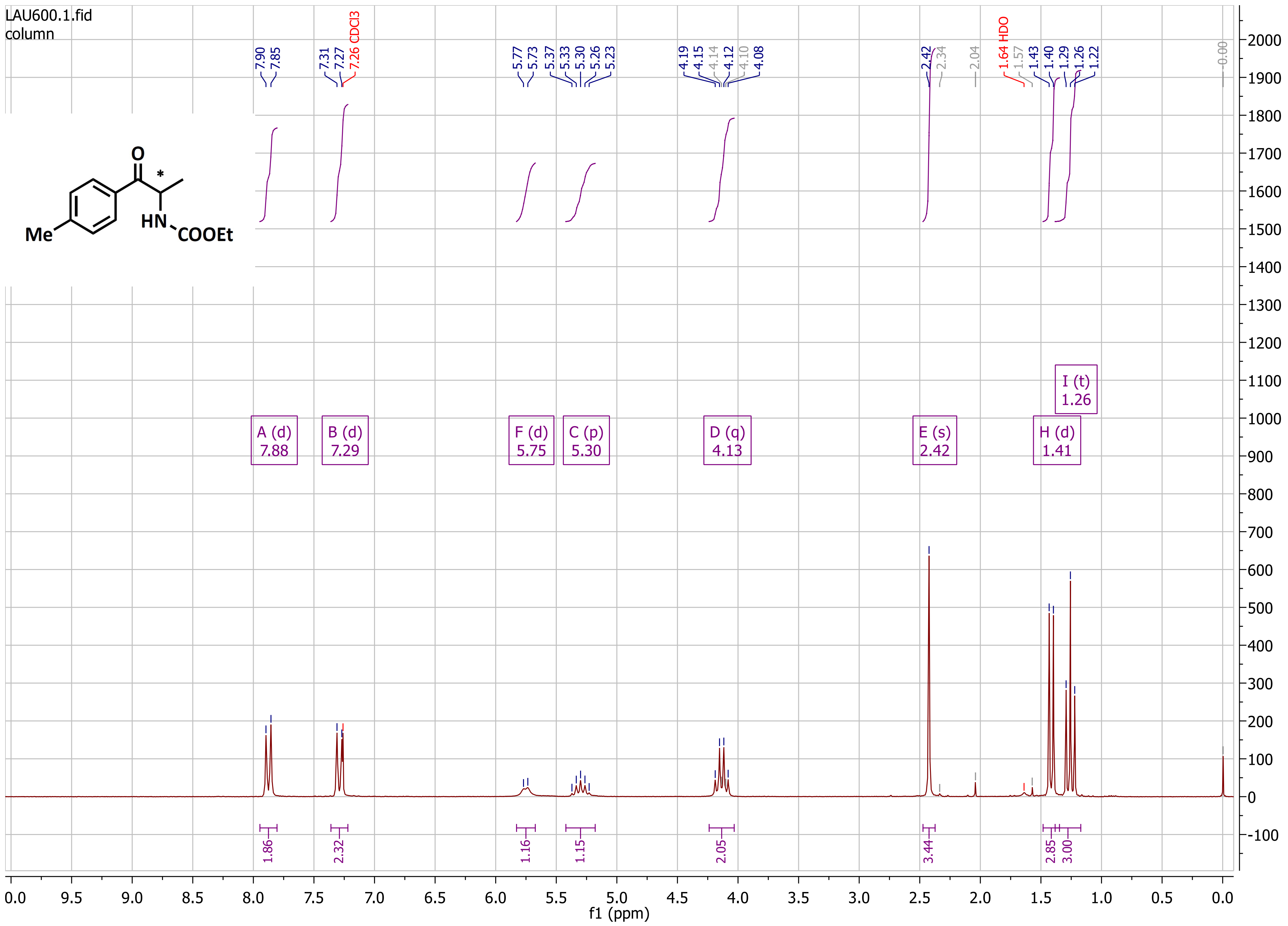

LAU600.1.fid
column
Me
O
*
HN
COOEt
7.90
7.85
7.31
7.27
7.26 CDCl3
5.77
5.73
5.37
5.33
5.30
5.26
5.23
4.19
4.15
4.14
4.12
4.10
4.08
2.42
2.34
2.04
1.64 HDO
1.57
1.43
1.40
1.29
1.26
1.22
-0.00
A (d) 7.88
B (d) 7.29
F (d) 5.75
C (p) 5.30
D (q) 4.13
E (s) 2.42
H (d) 1.41
I (t) 1.26
1.86
2.32
1.16
1.15
2.05
3.44
2.85
3.00
f1 (ppm)

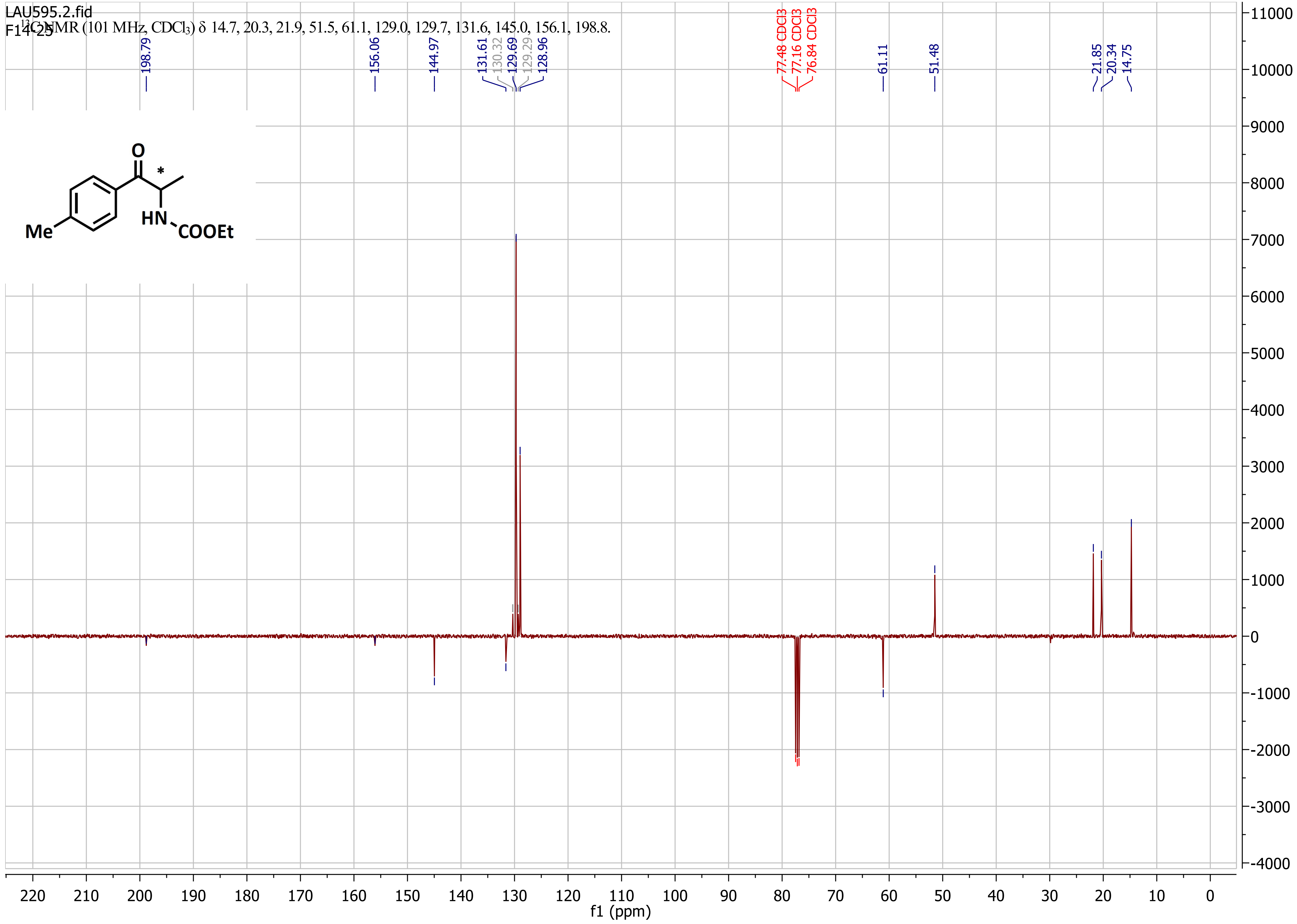
LAU595.2.fid
F14-25
13C NMR (101 MHz, CDCl3) δ 14.7, 20.3, 21.9, 51.5, 61.1, 129.0, 129.7, 131.6, 145.0, 156.1, 198.8.
198.79
156.06
144.97
131.61
130.32
129.69
129.29
128.96
77.48 CDCl3
77.16 CDCl3
76.84 CDCl3
61.11
51.48
21.85
20.34
14.75
O
*
HN
COOEt
Me
f1 (ppm)

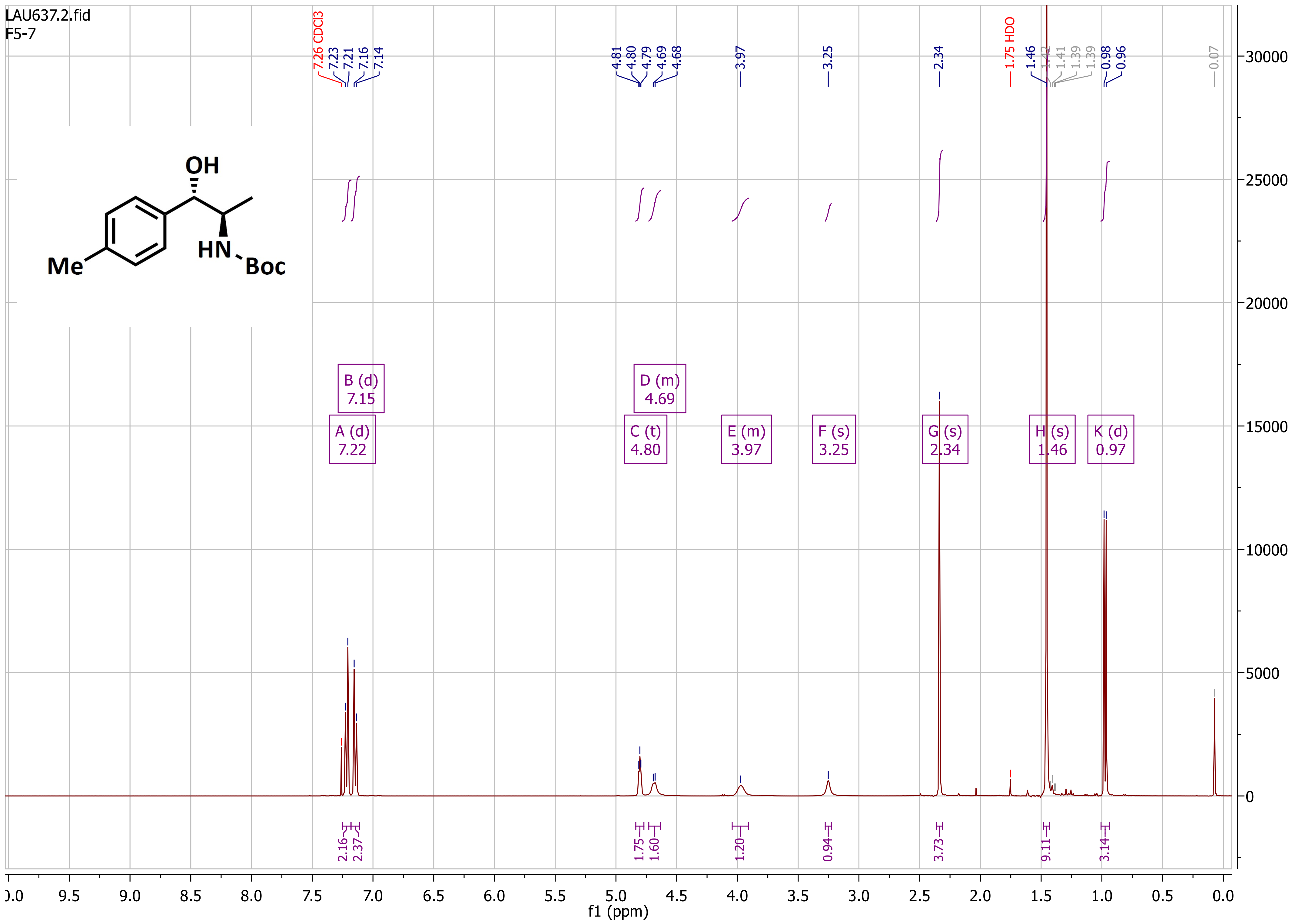
LAU637.2.fid
F5-7
OH
HN
Boc
Me
7.26 CDCl3
7.23
7.21
7.16
7.14
4.81
4.80
4.79
4.69
4.68
3.97
3.25
2.34
1.75 HDO
1.46
1.41
1.39
1.39
0.98
0.96
0.07
B (d)
7.15
A (d)
7.22
D (m)
4.69
C (t)
4.80
E (m)
3.97
F (s)
3.25
G (s)
2.34
H (s)
1.46
K (d)
0.97
2.16
2.37
1.75
1.60
1.20
0.94
3.73
9.11
3.14
f1 (ppm)

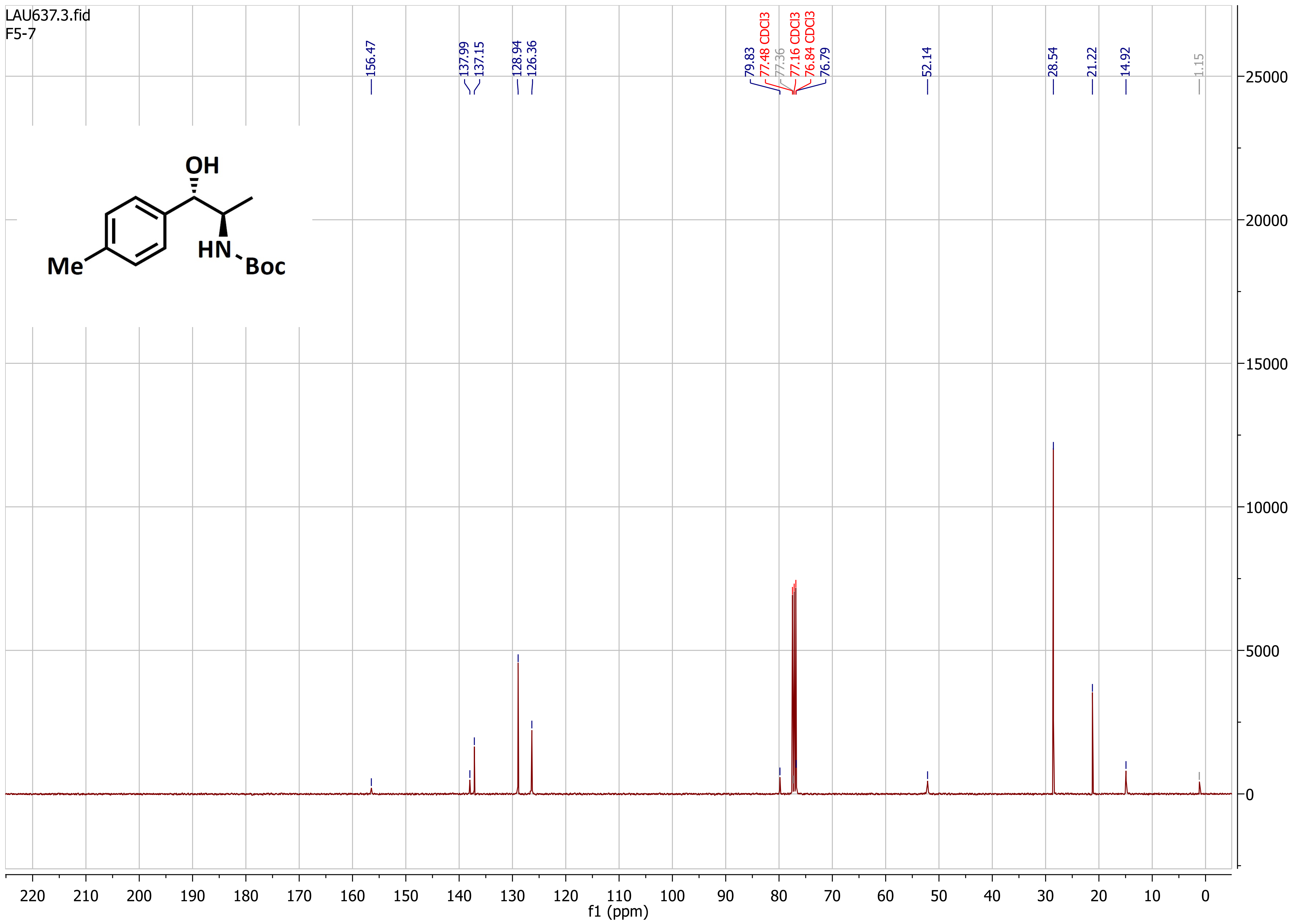

LAU637.3.fid
F5-7
156.47
137.99
137.15
128.94
126.36
79.83
77.48 CDCl3
77.36
77.16 CDCl3
76.84 CDCl3
76.79
52.14
28.54
21.22
14.92
1.15
OH
HN
Boc
Me
f1 (ppm)

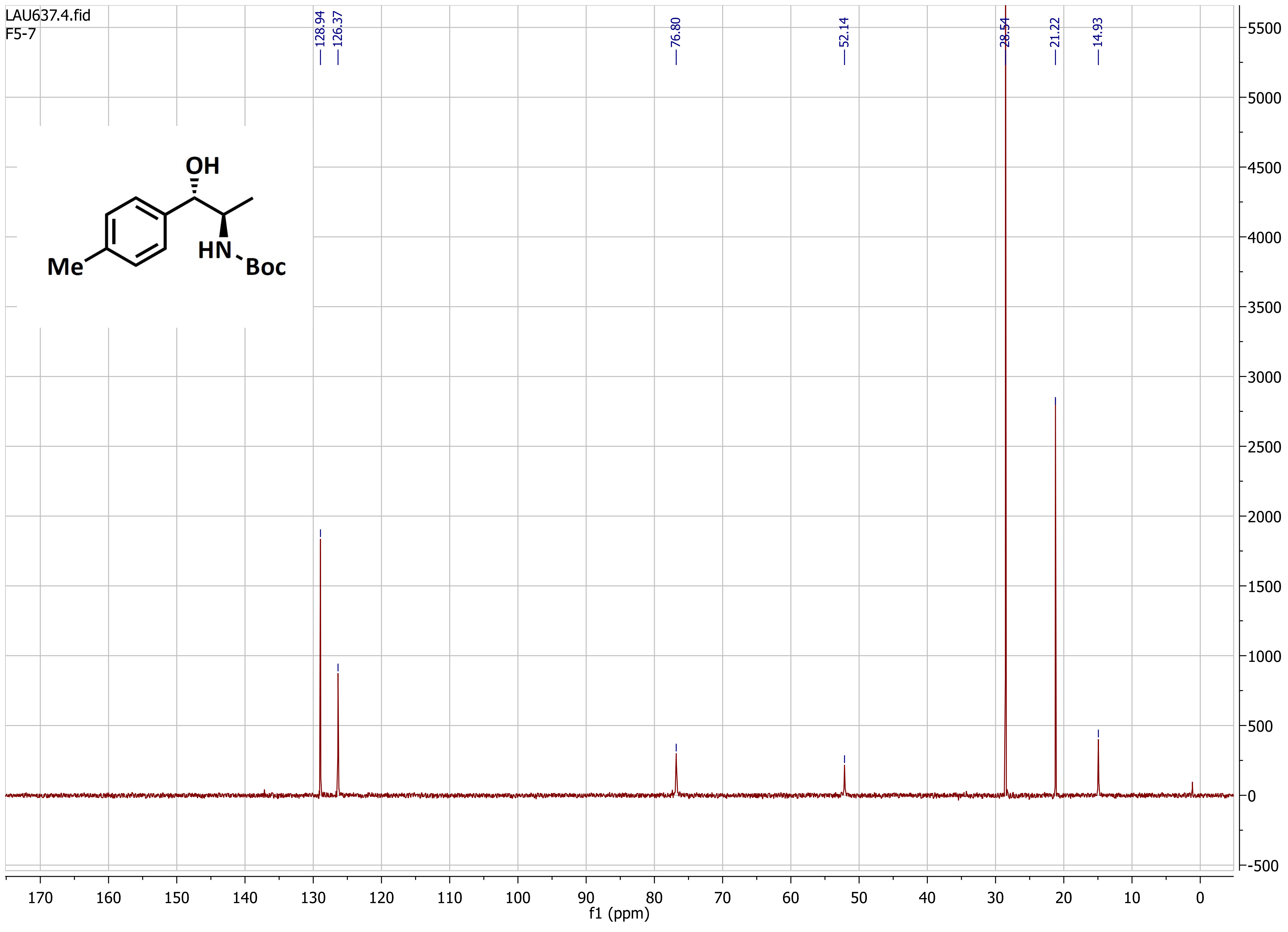
LAU637.4.fid
F5-7
128.94
126.37
76.80
52.14
28.54
21.22
14.93
OH
Me
HN
Boc
f1 (ppm)

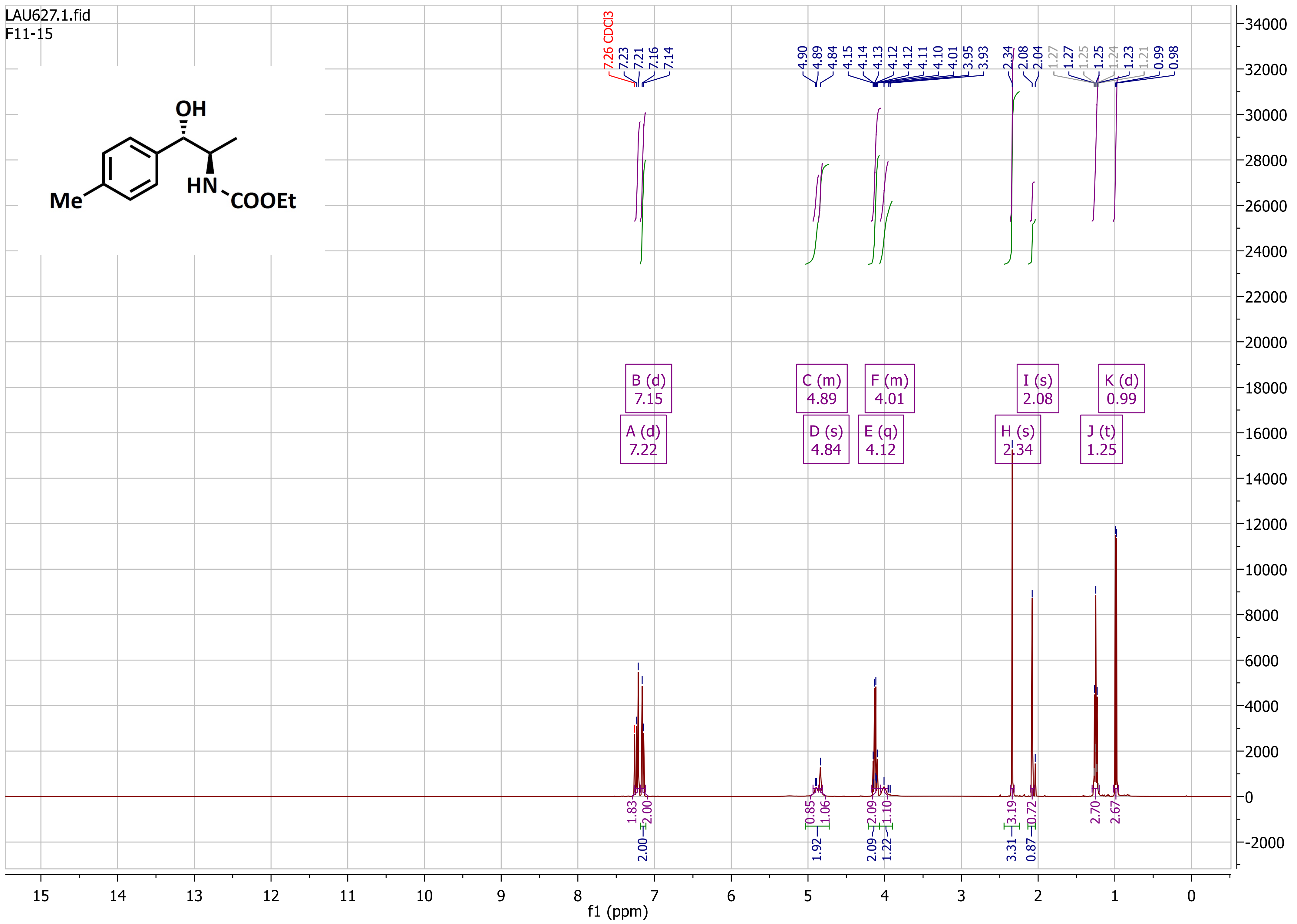

LAU627.1.fid
F11-15
OH
HN
COOEt
Me
7.26 CDCl3
7.23
7.21
7.16
7.14
4.90
4.89
4.84
4.15
4.14
4.13
4.12
4.12
4.11
4.10
4.01
3.95
3.93
2.34
2.08
2.04
1.27
1.27
1.25
1.25
1.24
1.23
1.21
0.99
0.98
B (d) 7.15
A (d) 7.22
C (m) 4.89
D (s) 4.84
F (m) 4.01
E (q) 4.12
I (s) 2.08
H (s) 2.34
K (d) 0.99
J (t) 1.25
1.83
2.00
2.00
0.85
1.06
1.92
2.09
1.10
2.09
1.22
3.19
0.72
3.31
0.87
2.70
2.67
f1 (ppm)

LAU627.5.fid

^{13}C NMR (101 MHz, $CDCl_3$) δ 14.5, 14.6, 21.1, 52.3, 61.0, 76.3, 126.1, 128.9, 137.2, 137.7, 156.8.

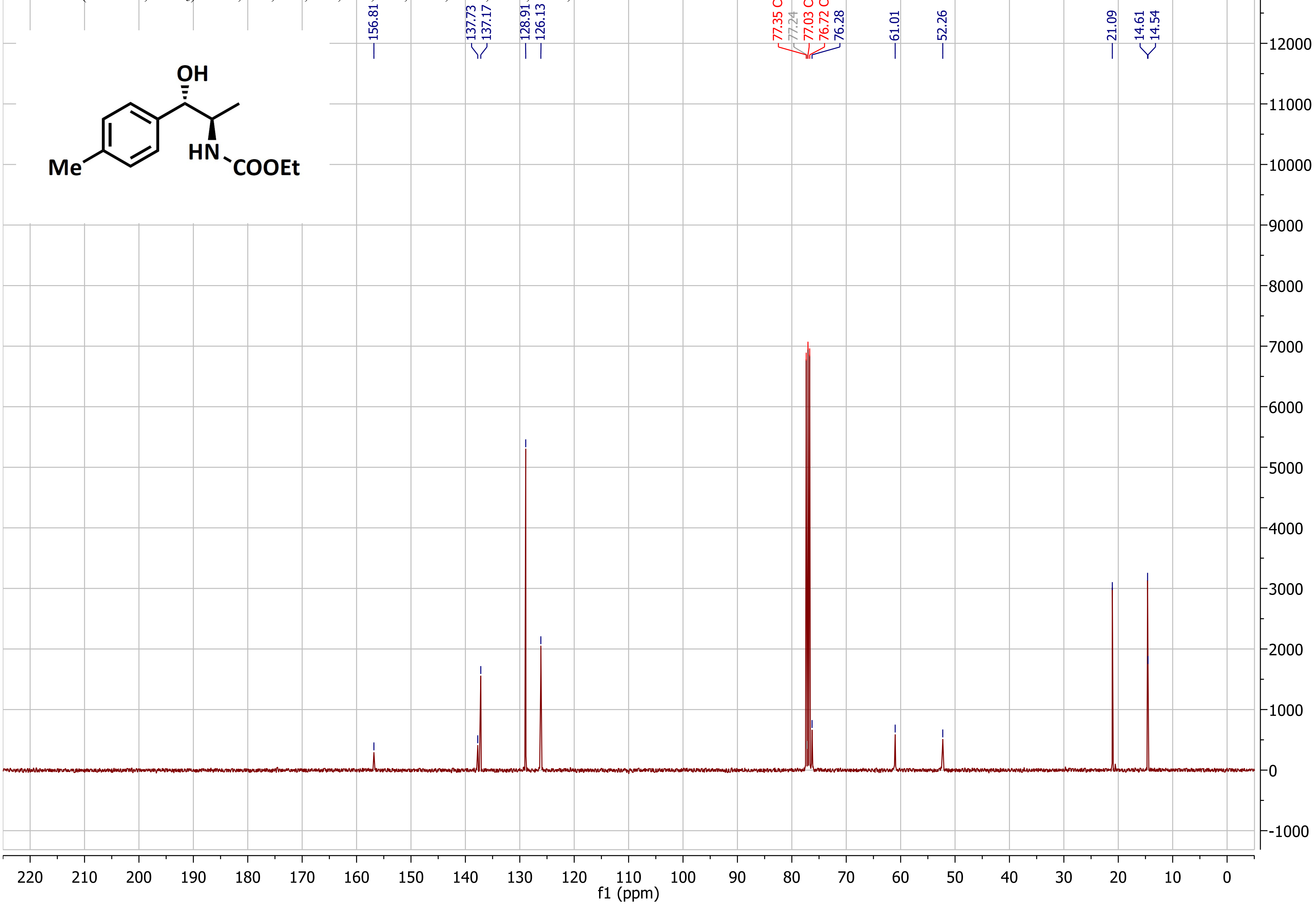

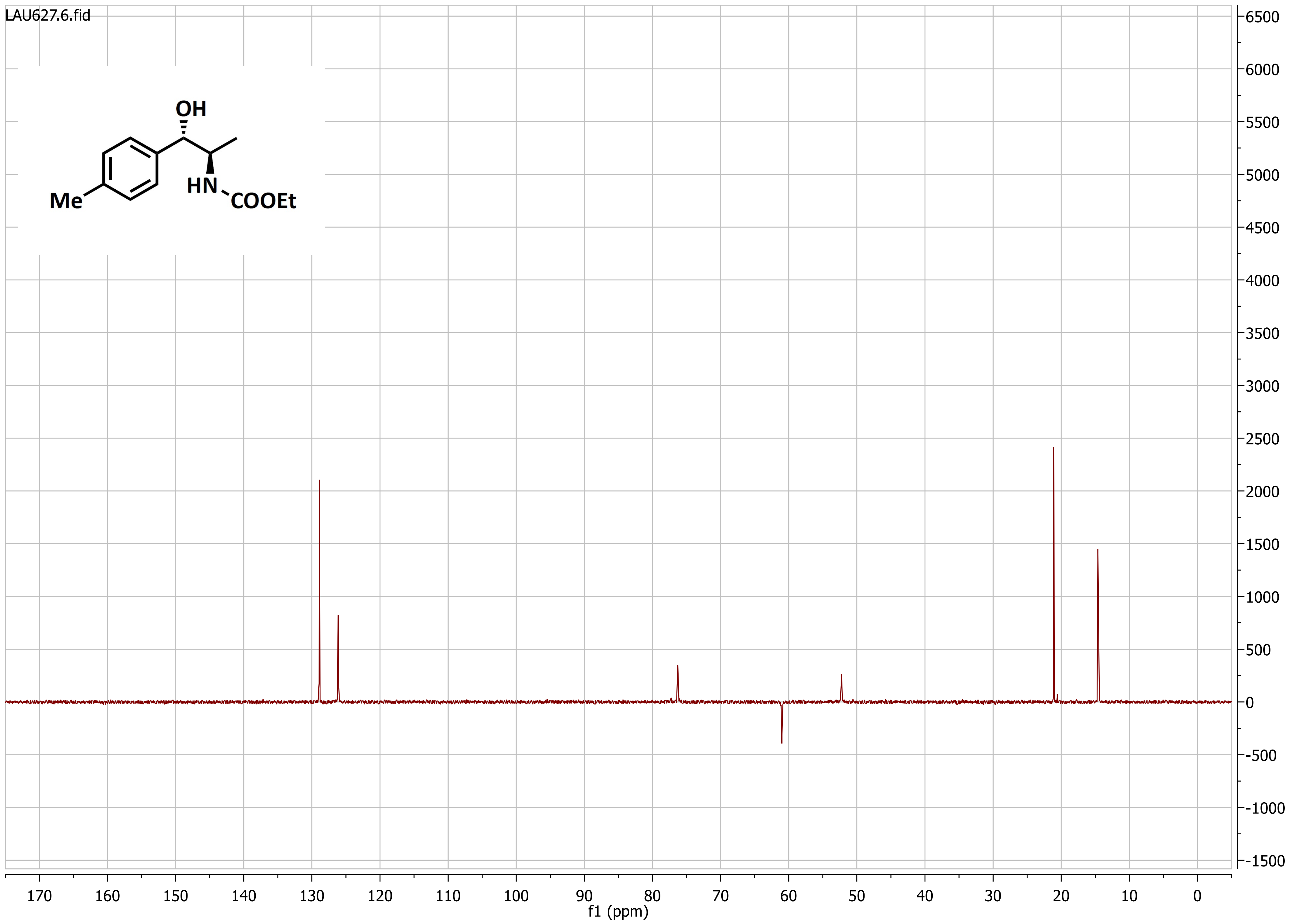

LAU627.6.fid
OH
Me
HN
COOEt
f1 (ppm)

LAU631.5.fid
Hydrochloride

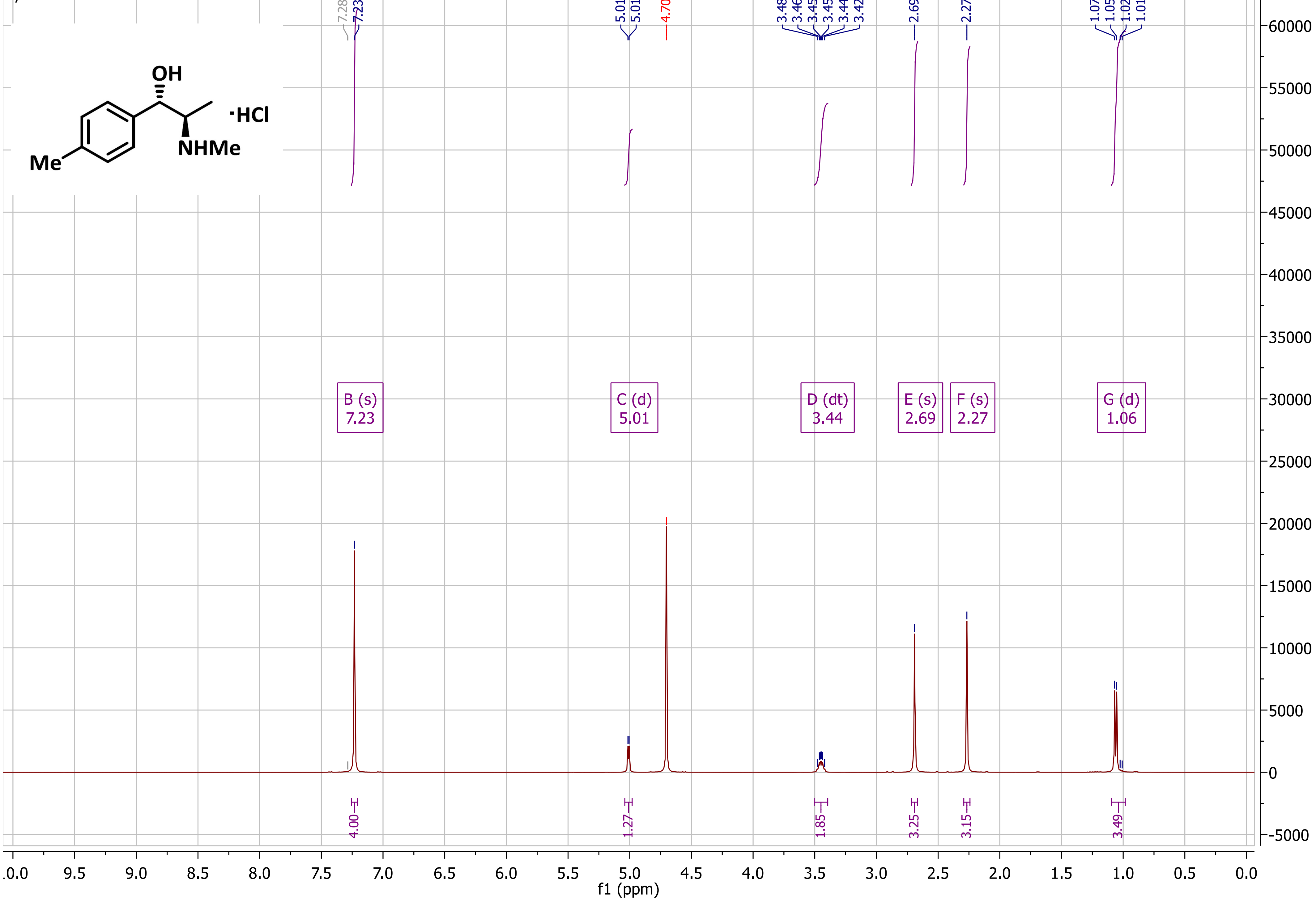

LAU631.6.fid
Hydrochloride

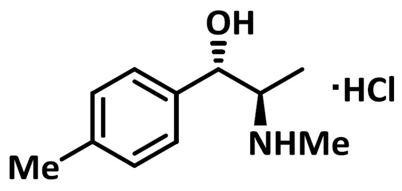

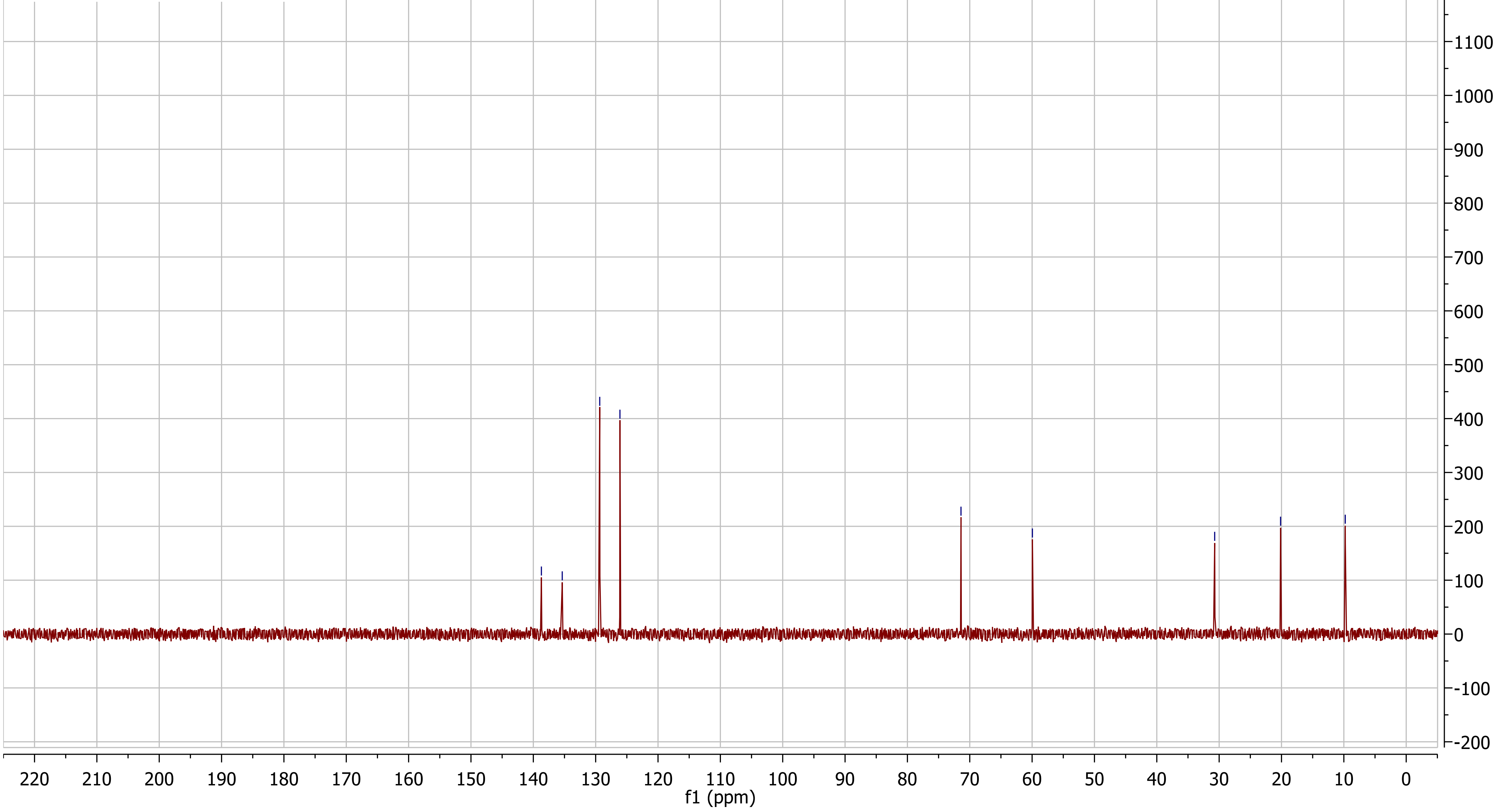

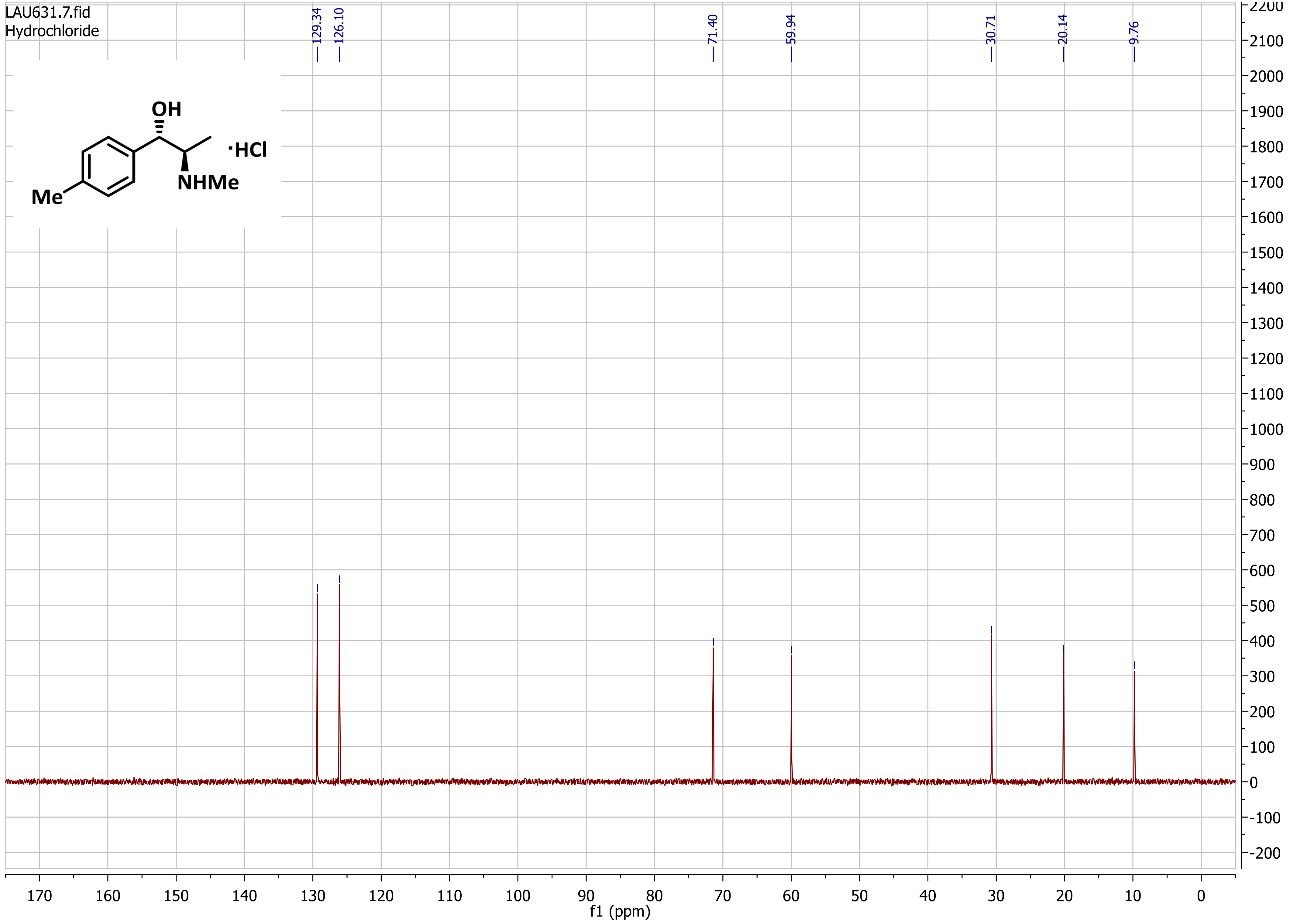
LAU631.7.fid
Hydrochloride
OH
·HCl
NHMe
Me
129.34
126.10
71.40
59.94
30.71
20.14
9.76
f1 (ppm)
